# Phylogenetic relationships and evolutionary patterns of the genus *Psammolestes* Bergroth, 1911 (Hemiptera: Reduviidae: Triatominae)

**DOI:** 10.1186/s12862-022-01987-x

**Published:** 2022-03-12

**Authors:** Carolina Hernández, Mateo Alvarado, Fabian C. Salgado-Roa, Nathalia Ballesteros, Nicol Rueda-M, Jader Oliveira, Kaio Cesar Chaboli Alevi, Joao Aristeu da Rosa, Plutarco Urbano, Camilo Salazar, Juan David Ramírez

**Affiliations:** 1 Centro de Investigaciones en Microbiología y Biotecnología-UR (CIMIBIUR), Facultad de Ciencias Naturales, Universidad del Rosario, Bogotá, Colombia; 2 Grupo de Genética Evolutiva y Filogeografía, Departamento de Biología, Facultad de Ciencias Naturales, Universidad del Rosario, Bogotá, Colombia; 3 School of BioSciences, The University of Melbourne, Parkville, VIC 3010 Australia; 4 Universidade Estadual Paulista (UNESP), Faculdade de Ciências Farmacêuticas, Araraquara, Sao Paulo 01000 Brazil; 5 Universidade de São Paulo (USP), Faculdade de Saúde Pública, São Paulo, SP Brazil; 6 Grupo de Investigaciones Biológicas de la Orinoquia, Universidad Internacional del Trópico Americano (Unitrópico), Yopal, Colombia

**Keywords:** *Psammolestes*, Niche divergence, Seasonal dry tropical forest, Triatominae, Rhodniini, Phylogenetic, Population genetics

## Abstract

**Background:**

The evolutionary history of biodiversity in South America has been poorly studied in the seasonal dry tropical forest (SDTF). Species diversification in this ecosystem may have a twofold explanation. First, intermittent connections in the middle and late Pleistocene promoted species dispersal and/or genetic connectivity between lineages isolated in disjunct patches of forest. Second, allopatric speciation proceeded immediately after the formation and colonization of the SDTF in the Neogene. Here we studied the diversification of *Psammolestes*, a genus endemic of the SDTF and naturally infected with *Trypanosoma cruzi* (agent of Chagas disease), using a combination of phylogenetic, population genetics and niche model methods, and evaluated the reliability of the three morphospecies currently recognized.

**Results:**

Our multilocus analyses recovered *P. coreodes* and *P. tertius* in a monophyletic clade sister to *P. arthuri*. Species delimitation tests recovered these lineages as different species despite the shared genetic variation observed between *P. coreodes* and *P. tertius* in five genes. Also, genetic variation of the genus clustered in three groups that were consistent with the three morphospecies. Our demographic model predicted a scenario of divergence in absence of gene flow, suggesting that mixed haplotypes may be the result of shared ancestral variation since the divergence of the subtropical-temperate species *P. coreodes* and *P. tertius*. In contrast, the tropical species *P. arthuri* was highly differentiated from the other two in all tests of genetic structure, and consistently, the Monmonier’s algorithm identified a clear geographical barrier that separates this species from *P. coreodes* and *P. tertius*.

**Conclusions:**

We found three genetically structured lineages within *Psammolestes* that diverged in absence of gene flow in the late Miocene. This result supports a scenario of species formation driven by geographical isolation rather than by divergence in the face of gene flow associated with climatic oscillations in the Pleistocene. Also, we identified the Amazon basin as a climatic barrier that separates tropical from subtropical-temperate species, thus promoting allopatric speciation after long range dispersion. Finally, each species of *Psammolestes* occupies different climatic niches suggesting that niche conservatism is not crucial for species differentiation. These findings influence the current vector surveillance programs of Chagas disease in the region.

**Supplementary Information:**

The online version contains supplementary material available at 10.1186/s12862-022-01987-x.

## Background

The Andes uplift and the formation of the Amazon Basin promoted species diversification via vicariance and/or dispersal which may be associated with climatic oscillations. Many examples from multiple organisms show the effect of such geological events in species differentiation [1–6], but only a handful show the role of geomorphology and climatic variations in the diversification of species from the seasonal dry tropical forest (SDTF) [7–9]. In tropical Americas, this ecosystem includes disjunct patches characterized by relatively low rainfall and high climatic seasonality [8, 10].

Species diversification in SDTF may be the result of these dry forest patches being intermittently connected during cold and dry periods in the middle and late Pleistocene, thus promoting species dispersal and/or genetic connectivity between isolated lineages ([8, 11]; the Pleistocene Arc hypothesis). Alternatively, such diversification may be due to genetic differentiation in allopatry, that could either be coupled or not with occasional long distance dispersal events [12–15]. For example, the diversification of geckos of the genus *Phyllopezus* was not influenced by Pleistocene climatic oscillations, but show a high phylogenetic structure associated with Miocene geomorphology [16]. In contrast, divergence in birds of the genus *Phacellodomus* and arthropods such as *Nephila* or *Drosophila gouveai* seems to be a consequence of Pleistocene climatic variation [11, 17, 18]. Also, studies in plants suggest that a combination of both climatic and geological changes were important for their diversification [9, 19, 20]. However, studies on the matter are scarce, and more evidence is needed to understand the evolutionary history of species inhabiting STDF [8].

The genus *Psammolestes* belongs to the subfamily Triatominae that excels between the subfamilies of Reduviidae due to their hematophagous behavior, but specially for being vectors of *Trypanosoma cruzi* [21] (Kinetoplastida, Trypanosomatidae), which causes the Chagas disease [22]. As Chagas disease has no effective treatment (e.g. vaccine), vector control strategies arise as alternatives to prevent and control the spread of not only the Chagas disease, but other tropical diseases as well [23–25]. The establishing of successful vector control strategies could benefit from a deep understanding of the vector’s biology, ecology, and evolution [26–28].

The genus *Psammolestes* (Reduviidae: Triatominae: Rhodniini) occurs in SDTF in apparent association with nests of Furnariidae birds [29–33]. This genus comprises three species, *P. arthuri* (Pinto, 1926), *P. tertius* (Lent & Jurberg, 1965) and *P. coreodes* (Bergroth, 1911), whose ecology and behavior remain largely unknown [31]. *Psammolestes arthuri* occurs across the eastern plains of Colombian and Venezuela, *P. tertius* is found in coastal regions near the Cerrado, Caatinga and the Mata Atlantica in Brazil, and *P. coreodes* distributes across the Chaco in Argentina, Paraguay, Bolivia, and Brazil [26, 34]. These species do not differ in karyotype [35–37], but are recognized based on morphological traits [31]. For example, *P. arthuri* is the most easily recognizable species based on a smooth and shiny cuticle in the thorax and the head, lack of cervical constriction, long hairs restricted to the apex of the second and third segments of the stylet, an anterolateral pronotal margin distinctly extended, and male genitalia with basal plate struts completely fused [31]. In addition, *P. tertius* and *P. coreodes* are recognized based on male genitalia morphology, anteocular distance, and post-ocular distance. Specifically, *P. tertius* has basal plate struts broadly S-shaped, while those of *P. coreodes* are hook shaped. Also, the anteocular distance in *P. tertius* is at least 2× higher than its post-ocular distance, while that of *P. coreodes* is always less than 2× [31, 38]. Additionally, recent evidence reported the existence of hybrid inviability in controlled crosses between *P. tertius* and *P. coreodes* [38].

Species of *Psammolestes* were initially grouped into the tribe Psammolestini and separated from Rhodniini [26, 39], but later they were placed back within Rhodniini because they occur in arboreal habitats and have protuberances behind the eyes [31]. Nonetheless, *Psammolestes* and *Rhodnius* were kept as separate genera as the femur and head of *Psammolestes* are wider and shorter than those of *Rhodnius* [31]. These taxonomic classifications have been tested at the molecular level, and it is well known that *Rhodnius* is paraphyletic compared to *Psammolestes* [28, 40–44]. However, only one molecular study on the phylogenetic relationships in the Triatominae subfamily included the three species of *Psammolestes*, and found *P. arthuri* sister to *P. tertius* and this clade sister to *P. coreodes* [45]*.* These findings suggest that *Psammolestes* is a monophyletic clade within the *prolixus* group [45].

Additionally, multiple studies have revealed a major role of niche conservatism in the diversification of the subfamily Triatominae [46–49]. For example, at the macroevolutionary scale, Ceccarelli et al. [47] found that tropical species of Triatominae share the same niche despite their phylogenetic differences, while niche conservatism in temperate species is due to shared evolutionary history. Nevertheless, the effect of niche conservatism in the diversification of species of *Psammolestes* remains to be tested. This is especially relevant as *P. arthuri* is a tropical species but *P. tertius* and *P. coreodes* have temperate distributions.

In this study, we used phylogenetic, population genetics analyses, and niche modeling to test the existence of discrete lineages within *Psammolestes* and investigate the role of the niche in maintaining these species. Our hypothesis was that the Amazon basin acts as a dispersion barrier that separates tropical and subtropical-temperate species thus suggesting a major role of geomorphology events in the divergence of *Psammolestes*. This scenario predicts that: (i) *P. coreodes* and *P. tertius* are most closely related to each other than they are to *P. arthuri,* and (ii) species differentiation proceeds despite niche conservatism. The understanding of the biotic and abiotic processes that shape vector species diversity of tropical diseases, as well as, the factors involved in their speciation process are essential for the settlement of adequate strategies for disease transmission control [23].

## Results

### Molecular phylogenetics

The resulting ML gene topologies were not concordant. The CYTB and PJH topologies (see Additional files 1 and 2) recovered *P. coreodes* and *P. tertius* as sister monophyletic clades, while 28S, CISP, LSM, TRNA and UPCA topologies (see Additional files 3, 4, 5, 6 and 7) did not recover them as reciprocally monophyletic. However, all the seven gene topologies showed *P. arthuri* as a well-supported monophyletic clade. Topological discordance is likely due to differences in coalescence times between loci, where the process of lineage sorting occurred faster in genes with small population size [50]. Alternatively, gene flow could explain allele sharing (see below: “Assessment of different demographic models”).

**Fig. 1:**
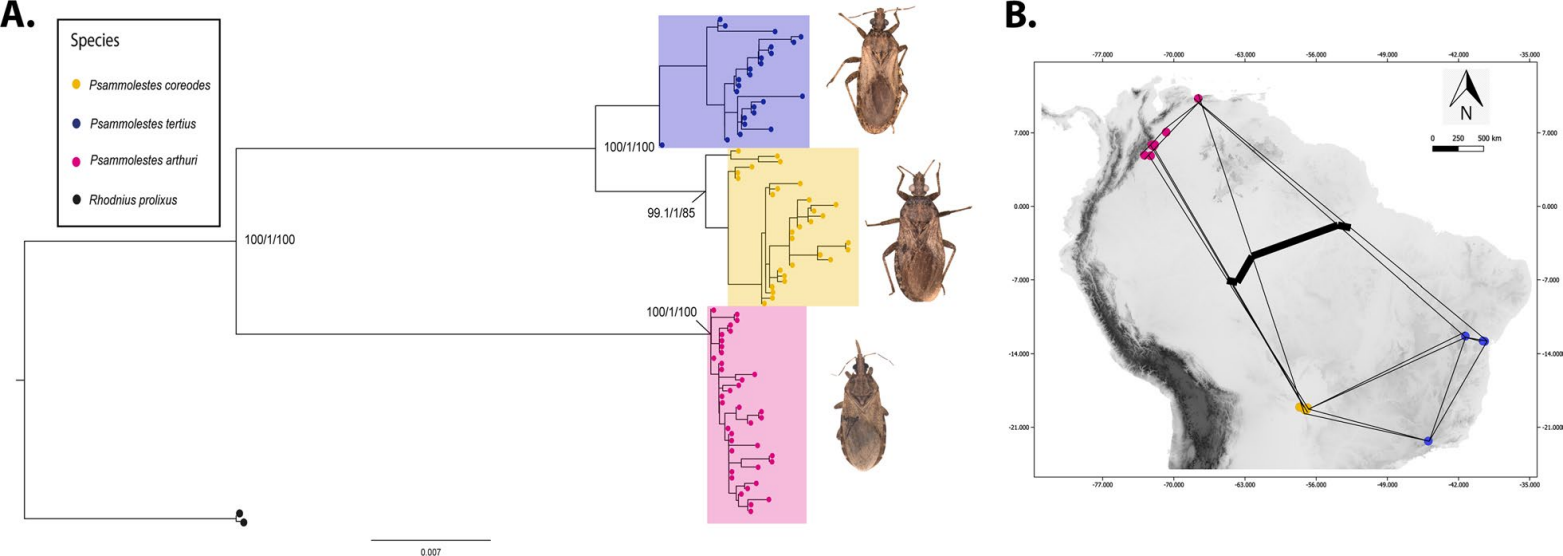
Maximum Likelihood phylogenetic reconstruction and geographical barrier test based on the seven molecular loci used in this study. **A** Phylogenetic reconstruction with the ML algorithm based on the seven molecular loci used in this study. Bootstrap values on the internal nodes are shown in the following order: SH-aLRT/aBayes/ultrafast bootstrap support. Only nodes with bootstrap values higher than 60 are shown. **B** Geographical barrier test (Monmonier’s algorithm) with the thick black line representing the main geographical barrier, and thin lines being the Voronoi tessellation and Delaunay triangulation

Our concatenated ML phylogenetic reconstruction recovered *P. coreodes* and *P. tertius* as sister species, and this clade was sister to *P. arthuri*. Overall, the three *Psammolestes* species were monophyletic with strong node supports (Fig. 1). Also, a multilocus Bayesian species coalescent (MSC) analysis revealed a species tree with the same topology than the ML tree with posterior probabilities > 0.96 (Additional file 8).

Finally, the mtDNA tree estimated by Bayesian inference also recovered the same relationships between the *Psammolestes* species with high posterior probabilities (see Additional file 9). Our dated phylogeny suggests that *P. arturi* diverged from the ancestor of *P. coreodes* and *P. tertius* 4.84 Mya (95% HPD interval = 1.32–10.38 Mya; see Additional file 9). We also found that the subtropical tempered species diverged 3.75 Mya (95% HPD interval = 0.92–8.15 Mya; see Additional file 9).

### Species delimitation tests

Three out of four models tested in BPP with nDNA loci recovered the known *Psammolestes* species. The only exception was the model ‘deep divergence and large population size’, which delimited two species: (i) *P. arthuri*, and (ii) *P. coreodes* + *P. tertius* (Table 1). Also, mtDNA delimited three species in the four models tested (Table 1). Consistently, mPTP strongly supported (ASV = 0.87) the same three independent lineages (see Additional file 10).

**Table 1:** Species delimitation by Bayesian phylogenetics and phylogeography program

Model	nDNA loci			mtDNA loci		
	Posterior	Species	Species delimited	Posterior	Species	Species delimited
Deep large	0.9950	2	*P. arthuri* *P. terius*/*P. coreodes*	1	3	*P. arthuri* *P. terius* *P. coreodes*
Deep small	1	3	*P. arthuri* *P. terius* *P. coreodes*	1	3	
Shallow large	1	3		0.8043	3	
Shallow small	1	3		1	3	

### Population genetics analyses

**Fig. 2:**
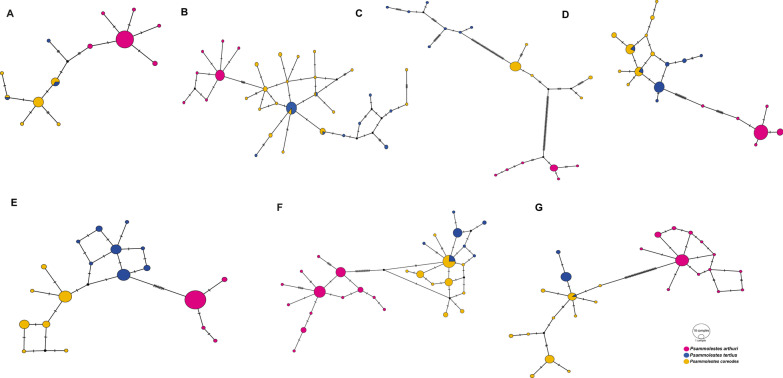
Haplotype networks obtained from the molecular data of the markers. **A** 28S, **B** CISP, **C** CYTB, **D** LSM, **E** PJH, **F** TRNA, **G** UPCA

**Fig. 3:**
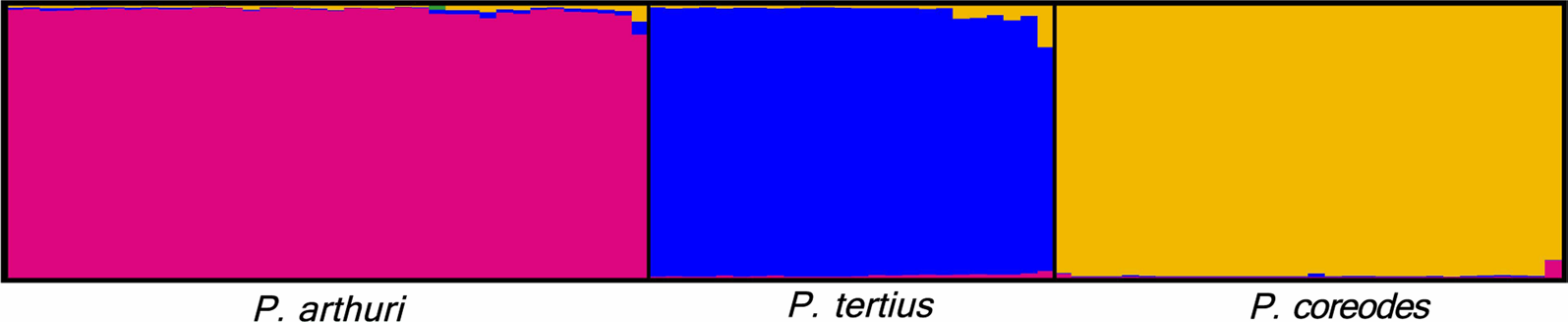
Population structure analysis plot values using STRUCTURE. Graphical output from the *distruct* software plotted using K = 3 and the matrix of aligned Q values from populations and individuals obtained from CLUMPP. Input sequences were organized from left to right in the following order*: P. arthuri*, *P. tertius*, and *P. coreodes*. Each bar represents an individual, and the color of the bar represents the likelihood of that individual of belonging to a population. Pink color represents the likelihood of belonging to *P. arthuri*, blue to *P. tertius*, and yellow to *P. coreodes*

Population substitution rate (θ) and nucleotide diversity (π) values were similar among the three *Psammolestes* species in each of the seven loci (Table 2). The three species showed signatures of population expansion in some loci, but this pattern was stronger and more consistent in *P. arthuri*. Consistently, haplotype networks displayed the typical star-like pattern where central haplotypes are coupled with multiple haplotypes with singletons (Fig. 2). In agreement with the haplotype networks, we detected stronger genetic differentiation between *P. arthuri* and both *P. coreodes* and *P. tertius* (see Additional files 11, 12, 13, 14, 15, 16 and 17), whereas genetic differences were weaker between *P. coreodes* and *P. tertius.* The structure algorithm recovered three clusters that were concordant with the three *Psammolestes* species (Fig. 3, see Additional files 10 and 18), although some *P. tertius* individuals showed shared ancestry with *P. coreodes*. Additionally, we found that isolation by distance contributed to the genetic structure observed in our data (Fig. 1B, Additional files 19, 20). This is mainly due to the geographical distance of *P. arthuri* compared to the other two species. Consequently, Monmonier’s algorithm [51] supports a geographical break that coincides with the Amazon basin (see Additional files 1, 2, 3, 4, 5, 6 and 7) splitting tropical species (*P. arthuri*) from temperate species (*P. coreodes* and *P. tertius*). This geographic break was recovered in all genes, suggesting that the tropical *P. arthuri* diverged from the other two temperate species in allopatry (Additional files 1, 2, 3, 4, 5, 6 and 7).

**Table 2:** Population genetics summary statistics for each species per locus

Statics	28S			CISP			CYTB			LSM			PJH			TRNA			UPCA		
	*P.art*	*P.cor*	*Pter*	*P.art*	*P.cor*	*Pter*	*P.art*	*P.cor*	*Pter*	*P.art*	*P.cor*	*Pter*	*P.art*	*P.cor*	*Pter*	*P.art*	*P.cor*	*Pter*	*P.art*	*P.cor*	*Pter*
n	35	16	4	20	28	25	13	21	7	36	26	23	37	28	30	50	39	22	40	29	16
h	7	6	3	7	19	9	6	6	7	8	9	7	5	8	8	15	10	7	14	11	3
S	8	6	5	6	19	11	11	27	27	29	6	7	14	17	5	38	9	7	43	12	2
θ	0.0035	0.0032	0.0049	0.0027	0.0080	0.0048	0.0070	0.0128	0.0222	0.0099	0.0022	0.0027	0.0051	0.0067	0.0019	0.0132	0.0033	0.0030	0.0167	0.0050	0.0010
π	0.0012	0.0020	0.0045	0.0012	0.0058	0.0045	0.0057	0.0082	0.0206	0.0051	0.0020	0.0018	0.0013	0.0034	0.0018	0.0051	0.0024	0.0020	0.0052	0.0056	0.0005
*D*_*T*_	− 1.92*	− 1.27*	− 0.79*	− 1.71*	− 0.97*	− 0.21	− 0.76	− 1.39	− 0.40	− 1.68*	− 0.31	− 0.96	− 2.38*	− 1.71*	− 0.15	− 2.06*	− 0.74	− 0.97	− 2.42*	0.36	− 1.03
R2	0.07	0.10	0.25	0.07*	0.08*	0.12	0.12	0.08*	0.17	0.05*	0.11	0.09	0.12	0.12	0.11	0.04*	0.08	0.09	0.13	0.13	0.13
Fu and Li’s F	− 2.82*	− 1.53	− 0.75	− 1.85	− 1.10	0.71	− 0.59	− 0.67	− 0.66	− 0.05	− 0.33	− 0.86	− 3.93^	− 3.00*	0.16	− 1.78	− 0.68	− 1.38	− 4.74^	0.19	− 0.73
Fu and Li’s D	− 2.63*	− 1.36	− 0.79	− 1.55	− 0.93	0.96	− 0.43	− 0.23	− 0.65	0.78	− 0.27	− 0.66	− 3.79^	− 2.94*	0.26	− 1.17	− 0.50	− 1.27	− 4.82^	0.07	− 0.50

n: number of samples, h: number of haplotypes, S: number of segregating sites, θ: population mutation rate, π: average pairwise distance, *D*_*T*_: Tajima’s D. R2: Ramos-Onsins & Rozas’ R2, Fu and Li’s F, and Fu and Li’s D

“*” symbolizes p < 0.05 and “^” symbolizes p < 0.02

### Assessment of different demographic models

Our results suggest that the demographic model of ‘divergence without gene flow’ fitted our data better than other models with unidirectional or bidirectional gene flow. However, this scenario shows some uncertainty (wAIC = 0.30) as AIC values were not considerable different between models (see Additional files 18 and 21).

### Environmental niche modeling

**Fig. 4:**
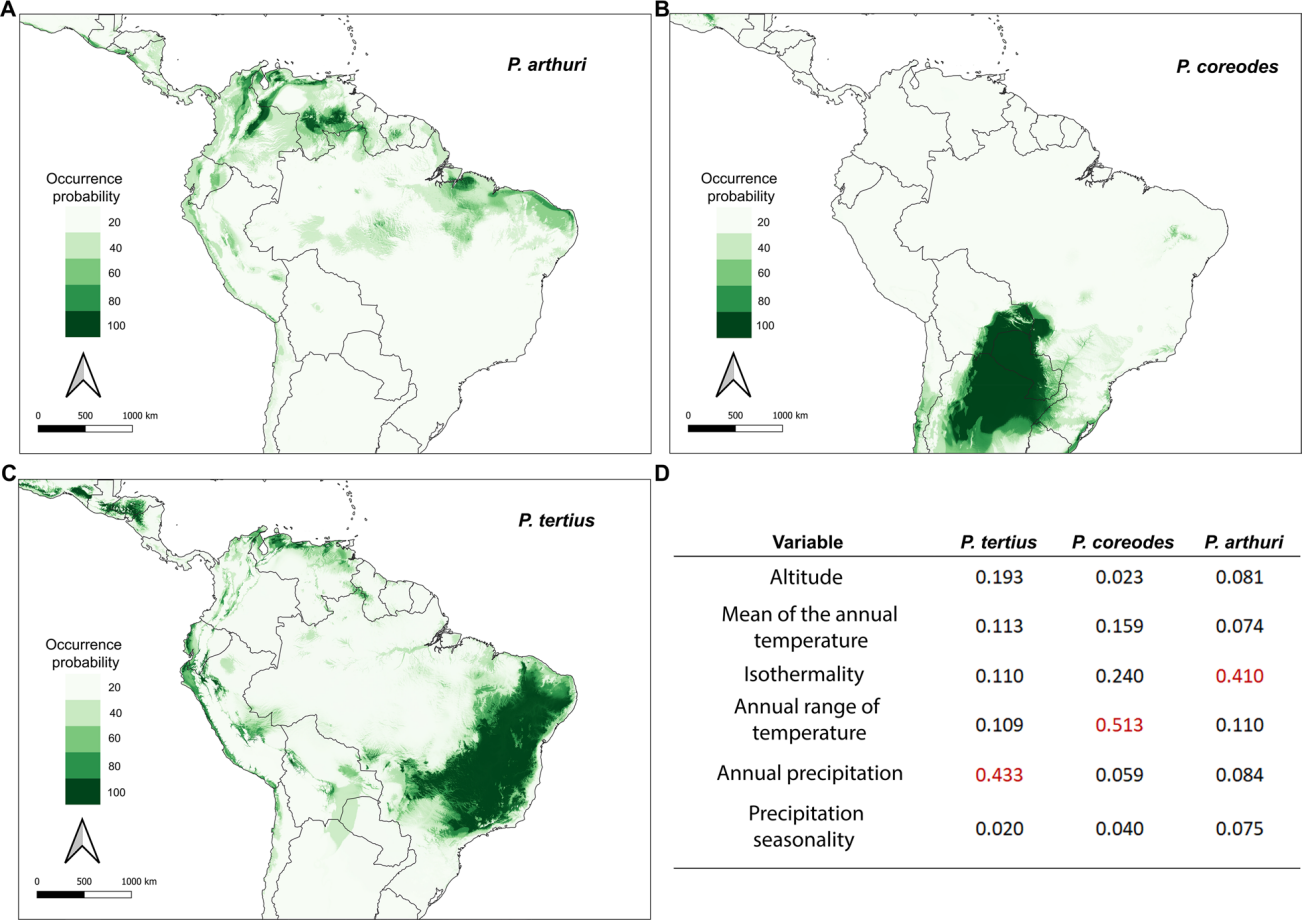
Environmental niche modeling test. **A** *P. arthuri* **B** *P. coreodes* **C** *P. tertius.* Highlighted areas in dark green correspond to the areas where it is more probable to find individuals of each one of the three species. **D** Variables measured on the models. Highlighted in red there is the variable that influences the most of the distribution of each one of the species

We found that the ensemble model fitted better than each independent algorithm (ROC > 0.95). This model showed different non-overlapping suitable areas for each species of *Psammolestes* (Fig. 4). Overall, areas with higher occurrence probability for the three species were restricted to dry environments such as tropical savannas and the amazon basin showed the lowest suitability values. Moreover, we discovered that the distribution of each *Psammolestes* species was determined by different environmental variables: annual precipitation for *P. tertius,* annual range of temperature for *P. coreodes*, and isothermality for *P. arthuri* (Fig. 4)*.* Consistently, the niche equivalence tests indicate that climatic niches of these species have diverged (Table 3).

**Table 3:** Niche overlap test (NOT) and Niche Divergence test (NDT) results for each one of the combinations between *Psammolestes* species

Species 1	Species 2	Niche overlap test (NOT)				Niche divergence test (NDT)				Interpretation
		Equivalency test		Background test		Equivalency test		Background test		
		d	p value	d	p value	d	p value	d	p value	
*P. arthuri*	*P. tertius*	0.03309	0.00099	0.16666	0.375	0.03309	0.00099	0.14285	0.1666	Strong evidence niches have diverged
*P. arthuri*	*P. coreodes*	0.00189	0.00099	0.1578	0.768	0.00189	0.00099	0.456	0.9809	Strong evidence niches have diverged
*P. coreodes*	*P. tertius*	0.00351	0.00099	0.2	0.05882	0.00351	0.00099	0.00099	0.0625	Strong evidence niches have diverged

## Discussion

We recovered three well supported lineages that are concordant with the previously described morphospecies and experimental crosses: *P. coreodes, P. tertius* and *P. arthuri* [38]. Both phylogenetic and population genetics analyses indicate that *P. coreodes* and *P. tertius* are genetically more similar than they are to *P. arthuri*. The species distribution analyses suggest that these species are restricted to tropical savannas and have a low probability of occurrence in humid areas. These findings support a role for the Amazon basin as an absolute barrier for the dispersal of species of *Psammolestes*.

Our phylogenetic reconstruction contrasts with a previous study where *P. tertius* and *P. arthuri* were recovered as sister species (bootstrap support = 66%), and this clade sister to *P. coreodes* (bootstrap support = 87%) [45]. However, here we obtained higher support values in our ML tree (Fig. 1A) and the species tree (Additional file 9A) for the monophyly of the clade composed by *P. tertius* and *P. coreodes,* sister to *P. arthuri*. This result is consistent both with the geographic and genetic distance between these taxa. Despite of the contentious systematics of the genus [28, 40–44], all our analyses validate the existence of three lineages of *Psammolestes* thus supporting the original species description based on morphological traits [31, 35, 38].

Our mtDNA divergence times estimation, the strong genetic structure we observed, and the absence of gene flow between species suggest that the diversification of *Psammolestes* is not explained by recent dispersal events across corridors in the forested Amazon basin nor by the Pleistocene arc hypothesis [7]. In contrast, our results agree with a scenario of allopatric differentiation via long distance dispersal event(s) across the Amazon in the late Miocene, followed by recent local geographic expansion as suggested by the Tajimas’ D value [52–55]. However, we cannot rule out that the current disjunct distribution of the different species of *Psammolestes* is the result of extinction in the Amazon basin. Interestingly, the diversification times of *Psammolestes* do not mirror those of *Phacellodomus rufifrons* (Furnariidae), a bird whose nests are commonly invaded by these kissing bugs [11] and whose diversification occurred in the presence of gene flow in the Pleistocene [11]. Therefore, the historical dispersion patterns of Furnariidae birds do not explain the diversification of *Psammolestes*. Nevertheless, future studies are needed to understand the evolutionary importance of this peculiar association with Furnariidae birds, which seems to be exclusive to these Triatominae species.

Our niche modeling results suggest that, although all species of *Psammolestes* occur in the SDTF, they have divergent niches shaped by different climatic predictors, indicating that niche conservatism does not play a role in the diversification of these triatomines. This finding agrees with previous studies that documented nonoverlapping niches for *P. coreodes* and *P. tertius* [30, 32]. Such an scenario of niche divergence agrees with the absence of gene flow between the three species and the inviability reported in experimental crosses between *P. tertius* and *P. coreodes* [38]. However, the relevance of other factors in species divergence, such as biotic interactions need to be investigated.

In summary, *Psammolestes* has three genetically structured species that also differ in their climate niches and morphology. They diverged in allopatry without gene flow, and their differentiation involved long distance dispersal event(s) across the Amazon basin (which is a current barrier for their dispersal). Further investigation is needed to elucidate the behavior and ecology of each species as well as the reproductive barriers maintaining their integrity. These findings are relevant in terms of understanding the transmission dynamics of Chagas disease and future improvement of vector control strategies in endemic countries.

## Materials and methods

### Sampling

**Fig. 5:**
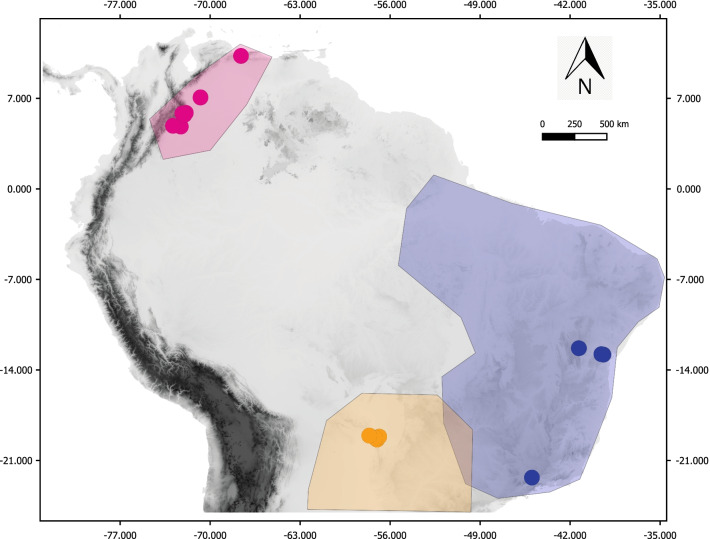
Distribution of the three *Psammolestes* species sampled in this study. Dots represent the sampling sites of this work while polygons symbolize previously reported sampling sites by Ceccarelli et al. [34], where the *Psammolestes* species have been found. *P. arthuri* is represented by the pink color, *P. coreodes* with yellow, and *P. tertius* with blue

We collected a total of 92 individuals of the three *Psammolestes* species, from 12 localities in Venezuela, Colombia, and Brazil (Fig. 5; Additional file 22). We also sampled *Rhodnius prolixus* to use as an outgroup in our phylogenetic inferences (see below). Outgroup selection was based on previous phylogenetic reconstructions, where *Psammolestes* was shown to be sister taxa to some of the *prolixus* group species (*Rhodnius* seems to be paraphyletic with respect to *Psammolestes*), including *R. prolixus* [45]. The samples obtained were preserved in absolute ethanol and stored at − 20 °C until needed. All collections were done under the permit 63257-2014 awarded to Universidad del Rosario by the ANLA (Autoridad Nacional de Licencias ambientales).

### Ethical statement

This study was submitted and approved by the ethics committee of Universidad del Rosario entitled “*Genómica, evolución y biogeografía de especies del género Rhodnius: vectores de la enfermedad de Chagas”* act number 007/2016.

### Extraction, amplification, and alignment of DNA data

We extracted DNA from leg tissue, using the DNeasy^®^ Blood & Tissue kit, with modifications in the original protocol suggested by the manufacturer for extractions in insects [56]. We amplified and sequenced seven loci to explore phylogenetic relations among *Psammolestes*: Four new nuclear loci, tRNA Guanine (37) -N (1) methyltransferase (TRNA), Putative juvenile hormone inducible protein (PJH), Probable cytosolic iron sulfur protein assembly protein Ciao 1 (CISP), Lipoyl synthase, mitochondrial (LSM), along with the previously reported Uncharacterized Protein for Cell Adhesion (UPCA) [57, 58] and two loci previously used in Rhodniini tribe phylogenetic analyses, 28S rRNA (28S) [43] and Cytochrome b (CYTB) [28, 59] (see Additional file 23). Amplicons were visualized on a 1.5% agarose gel and the products amplified were purified using the PCR kit ExoSAP-IT Product Cleanup (Affymetrix, Santa Clara, CA, USA) and bidirectionally sequenced by the Sanger method. Contigs were assembled, checked, and edited in CLC Main Workbench 20.0 (https://digitalinsights.qiagen.com). Sequence alignment per locus was performed using MAFFT [60] and the results were visually inspected and manually corrected if necessary, using Mesquite [61]. We ran PHASE algorithm with 1000 iterations per simulation implemented in DnaSP v6.12.03 [62] to resolve alignment ambiguities. Finally, we generated a concatenated alignment with the seven loci in Mesquite (nuclear and mitochondrial: 4.342 bp) [61]. Sequences from this study were submitted in GenBank and numbers can be visualized in Additional file 24.

### Molecular phylogenetic analysis

We reconstructed phylogenetic relationships among the three *Psammolestes* species for each locus and the concatenated alignment (one partition per locus) using maximum likelihood (ML) inference in IQ-Tree 2 [63]. We selected the best substitution model for each case using the IQ-Tree 2 tool ModelFinder [64] based on the Bayesian Information Criterion (BIC; Schwarz, 1978). The substitution model selected for each locus was: HKY + F for 28S rRNA (28S) and RNA Guanine (37) -N (1) methyltransferase (TRNA), F81 + F + I for Lipoyl synthase, mitochondrial (LSM), F81 + F for Probable cytosolic iron sulfur protein assembly protein Ciao 1 (CISP), HKY + F + I for Putative juvenile hormone inducible protein (PJH), K2P for Uncharacterized Protein for Cell Adhesion (UPCA), and HKY + F + G4 for Cytochrome b (CYTB). Node support was assessed with UltraFast Bootstrap [66], aBayes [67] and SH-aLRT [68] with 10,000 pseudoreplicates in all cases. For the partitioned analysis, node supports were calculated by resampling both the partitions and the sites within the resampled partitions [69].

We also estimated the *Psammolestes* species tree using multilocus coalescence species approach in BEAST2 v.2.6.6 with genes included in this study [70, 71]. We executed three independent runs of 50 million generations, sampling every 1000 generations with burn-in of 15.000 chains. We determined the appropriate molecular clock in MEGA 10.0 [72] and used relaxed uncorrelated lognormal clocks for all partitions. We selected Yule model for speciation process and used the best models of substitutions estimated in IQ-tree [63]. The LogCombiner v.1.10.4 [73] tool was used to combine independent log files and species trees files obtained in each run (three). Trees were visualized in DensiTree v.2.1. The convergence of the chains in the model was examined by confirming the trace files in Tracer v.1.7.1 [74], obtaining an effective sample size of > 200 for all parameters. Lastly, maximum credibility tree was produced in Tree Annotator with burn in of 10% and visualized in Figtree [74].

Finally, we estimated divergence times using the mitochondrial locus CYTB in BEASTv.2.6.6 [70]. We only used this locus, because is the only one with a reported substitution rate, which is 0.012–0.018 substitution/site/million years, and has been used for node dating in previous works [75, 76]. We used a Yule model with two independent runs of 80 million generations, sampled every 1000 generations. We examined the convergence of the chains in Tracer [74] to confirm that the effective sample sizes of the parameters were > 200. We combined the independent runs in Logcombiner [73, 77] and selected the maximum credibility tree in tree annotator, discarding the 10% of the trees as burn-in .

### Species delimitation tests

We established the number of *Psammolestes* lineages with two delimitation methods: The Bayesian Phylogenetics and Phylogeography method (BPP; [78]) and the multi-rate Poisson Tree Processes method (mPTP; [79]). For the BPP analysis, we analysed the mtDNA and nDNA independently as recommended elsewhere [78]. We performed a species tree estimation and joint species delimitation for both datasets, assigning individuals to a “species” based on the results of the phylogenetic trees previously constructed [80]. We implemented four combinations of priors, for divergence times (t) and population size parameters (q), allowing to test different evolutionary scenarios: large population sizes (*θ* = *G* (1, 10)), shallow population sizes (*θ* = *G* (2, 2000)), deep divergence times (t = G (1, 10)) and shallow divergence times (t = G(2, 2000)). Each analysis used 100,000 iterations per run, sampling every 2 iterations, and using 10% of the iterations in the chain as burn-in.

We used the best ML concatenated tree for the mPTP method. The first step on this method is to calculate the minimum branch length of the tree, correcting the potential error when similar sequences are present. Then, we ran 10 MCMC replicates of 100,000,000 steps, sampling every 1000 steps, of which 10% were used as burn-in. Lineage congruence between both methods were considered as putative species following Carstens et al. [81].

### Population genetics analyses

We calculated the haplotype diversity (*h*), number of segregating sites (*S*), population substitution rate (θ), and nucleotide diversity (π) to characterize the genetic variability of each *Psammolestes* species in DNASP v6.12.03 [62]. Moreover, we determined the genetic structure among the three species of *Psammolestes* with a relative measure (F_ST_) and two absolute ones (D_a_, D_xy_). To evaluate deviations from panmixia, we implemented the Hudson permutation test [82] with 1000 replicates. We also computed three neutrality tests: Ramos-Onsins and Rozas R_2_ (R_2_; Ramos-Onsins and Rozas [83]), Tajima’s D (D; Tajima [84]) and Fu & Li’s F and D statistics (FF, FD; Fu and Li [85]), in order to examine possible signatures of population expansion or contraction. We constructed TCS haplotype networks [86] for each locus using PopArt v1.7 [87].

We explored the geographical diversification of *Psammolestes* testing for isolation by distance implementing a Mantel test in the R package *vegan* [88] and a linear regression between the genetic distance (1/1 − F_ST_) and the geographical distances calculated in the package *geosphere* [89]. Additionally, we employed the Monmonier’s algorithm [90] in the R package *adegenet* [91] using a Delaunay triangulation to detect possible boundaries associated with geographic barriers.

Lastly, STRUCTURE v2.3.4 [92] was implemented to determine the number of genetic clusters (K) present in our data. We ran the analysis with the admixture model with uncorrelated alleles using 100,000 MCMC iterations, sampling K values from 1 to 10, and 5 iterations per K, along with a burn-in length of 100,000. The best K value was selected following Evanno et al. [93] and plotting the mean likelihood L(k) and variance per K using the STRUCTURE HARVESTER (Earl and vonHoldt [94]; http://taylor0.biology.ucla.edu/structureHarvester/; Evanno et al. [95]). The results of the best identified values of k were summarized in clump [95] and plotted using *distruct* [96]*.*

### Environmental niche modelling

#### Species distribution modelling

Models were constructed using BIOMOD2 package [97] for each *Psammolestes* species using four algorithms: Artificial Neural Networks (ANN; [98]), Generalized Linear Models (GLM; [99], Generalized Boosting Models (GBM; [100]), and Maximum Entropy Models (MAXENT [101]). We obtained *Psammolestes* species occurrence records from DataTri [34]. As we do not have an absence record for these species, we generated a pseudoabsences database limited to areas in south America where: (i) other Triatominae species were recorded, but *Psammolestes* was absent, and (ii) environmental conditions are not suitable for these taxa [102, 103]. An equal weighting for presences and pseudo-absences (prevalence weights = 0.5) was applied for modeling as recommended [104]. Five environmental variables were used (annual mean temperature, isothermality, annual range of temperature, annual precipitation and precipitation seasonality) at spatial resolution of 1 km. These variables were chosen from the 19 CHELSA layers [105] because they exhibited correlation values < 0.5 among them. Additionally, we used a topographic variable (altitude) obtained from Reuter, Nelson and Jarvis [106]. Algorithms were calibrated using 80% of the occurrence points and evaluated the accuracy of the models with the remaining 20%. This procedure (cross-validation) was repeated three times. Three different ensemble models were generated for the three *Psammolestes* species based on the combination of the four models produced by the previously mentioned algorithms. Two metrics were used to choose the model that best predicts the distribution of the taxa: The True Skill Statistic (TSS) and the area under the curve (AUC) of the receiver-operating characteristic (ROC) [107]. Variable importance to the model was calculated based on the Pearson correlation coefficient between the model with all variables and model where each variable was omitted in turn, using BIOMOD2 package [97].

### Environmental niche of the parental species

We estimated the environmental niche equivalence between all pairs of *Psammolestes* species using R package *humboldt* [108]. To do this, the overlap *Schoener’s D* statistic was calculated. This statistic goes from 0 to 1, meaning no overlap and full overlap respectively [109]. D statistical significance was obtained comparing the realized niche overlap against a null distribution of 1000 randomly generated overlaps from the reshuffled occurrence dataset and tested whether niche background and niche equivalency were different from the expectations by chance at α = 0.05 [108]. This was done using the entire species distribution under comparison (niche overlap test = NOT) and using only the area where they overlap (niche divergence test = NDT) [110]. We interpreted the NOT and NDT results following Table 2 from Brown and Carnaval [110].

## Supplementary Information

**Additional file 1.** CYTB Phylogenetic reconstruction and Barrier test. (A) Phylogenetic reconstruction with the ML algorithm based on the mitochondrial marker CYTB (B) Barrier test algorithm based on molecular and geographical arrays. Bootstrap values on the internal nodes are shown in the following order: SH-aLRT/aBayes/ultrafast bootstrap support. Only nodes with bootstrap values higher than 60 are shown.

**Additional file 2. **PJH Phylogenetic reconstruction and Barrier test (A) Phylogenetic reconstruction with the ML algorithm based on the nuclear marker PJH (B) Barrier test algorithm based on molecular and geographical arrays. Bootstrap values on the internal nodes are shown in the following order: SH-aLRT/aBayes/ultrafast bootstrap support. Only nodes with bootstrap values higher than 60 are shown.

**Additional file 3.** 28S Phylogenetic reconstruction and Barrier test (A) Phylogenetic reconstruction with the ML algorithm based on the nuclear marker 28S (B) Barrier test algorithm based on molecular and geographical arrays (B). Bootstrap values on the internal nodes are shown in the following order: SH-aLRT/aBayes/ultrafast bootstrap support. Only nodes with bootstrap values higher than 60 are shown.

**Additional file 4.** CISP Phylogenetic reconstruction and Barrier test (A) Phylogenetic reconstruction with the ML algorithm based on the nuclear marker CISP (B) Barrier test algorithm based on molecular and geographical arrays. Bootstrap values on the internal nodes are shown in the following order: SH-aLRT/aBayes/ultrafast bootstrap support. Only nodes with bootstrap values higher than 60 are shown.

**Additional file 5.** LSM Phylogenetic reconstruction and Barrier test. (A) Phylogenetic reconstruction wit the ML algorithm based on the nuclear marker LSM (B) Barrier test algorithm based on molecular and geographical arrays. Bootstrap values on the internal nodes are shown in the following order: SH-aLRT/aBayes/ultrafast bootstrap support. Only nodes with bootstrap values higher than 60 are shown.

**Additional file 6.** TRNA Phylogenetic reconstruction and Barrier test. (A) Phylogenetic reconstruction with the ML algorithm based on the nuclear marker TRNA (B) Barrier test algorithm based on molecular and geographical arrays. Bootstrap values on the internal nodes are shown in the following order: SH-aLRT/aBayes/ultrafast bootstrap support. Only nodes with bootstrap values higher than 60 are shown.

**Additional file 7.** UPCA Phylogenetic reconstruction and Barrier test. (A) Phylogenetic reconstruction with the ML algorithm based on the nuclear marker UPCA (B) Barrier test algorithm based on molecular and geographical arrays. Bootstrap values on the internal nodes are shown in the following order: SH-aLRT/aBayes/ultrafast bootstrap support. Only nodes with bootstrap values higher than 60 are shown.

**Additional file 8.** Bayesian inference of species tree based on multilocus data (A) Maximum clade credibility tree based on Bayesian inference of the seven genes used in this study. The values observed represent posterior probabilities. (B) Bayesian species tree from multilocus data.

**Additional file 9.** Bayesian inference phylogenetics tree for the locus CYTB obtained in *BEAST. Horizontal purple bars illustrate the 95% HPD for the nodes’ divergence time. Branch with a posterior probability above 0.95 are show

**Additional file 10. **Posterior probabilities on nodes, calculated by the mPTP algorithm.

**Additional file 11. **Heatmaps calculated for three different statistics: A) Fst, B) Dxy and C) Da for three species based on the molecular data obtained from the nuclear marker 28S.

**Additional file 12. **Heatmaps calculated for three different statistics: A) Fst, B) Dxy and C) Da for three species based on the molecular data obtained from the nuclear marker CISP.

**Additional file 13. **Heatmaps calculated for three different statistics: A) Fst, B) Dxy and C) Da for three species based on the molecular data obtained from the mitochondrial marker CYTB.

**Additional file 14. **Heatmaps calculated for three different statistics: A) Fst, B) Dxy and C) Da for three species based on the molecular data obtained from the nuclear marker LSM.

**Additional file 15. **Heatmaps calculated for three different statistics: A) Fst, B) Dxy and C) Da for three species based on the molecular data obtained from the nuclear marker PJH.

**Additional file 16. **Heatmaps calculated for three different statistics: A) Fst, B) Dxy and C) Da for three species based on the molecular data obtained from the nuclear marker TRNA.

**Additional file 17. **Heatmaps calculated for three different statistics: A) Fst, B) Dxy and C) Da for three species based on the molecular data obtained from the nuclear marker UPCA.

**Additional file 18. **Demographic models created with Phylogeographic Inference Using Approximate Likelihoods (PHRAPL) to test the evolution of *Psammolestes*. (A) Divergence with no migration (B) Divergence with bidirectional migration between *P. coreodes* and *P. tertius*. (C) Divergence with bidirectional migration between *P. tertius* and *P. arthuri*. (D) divergence with bidirectional migration between *P. tertius* with *P. coreodes*, and *P. tertius* with *P. arthuri*. (E) Divergence with bidirectional migration between *P. coreodes* and *P. arthuri*. (F) Divergence with bidirectional migration between *P. tertius* with *P. coreodes*, and *P. coreodes* with *P. arthuri*. (G) Divergence with bidirectional migration between *P. tertius* with *P. arthuri*, and *P. arthuri* with *P. coreodes* (H) Divergence with bidirectional migration between the three *Psammolestes* species. Starting from this point, all of the demographic models include bidirectional migration between *P. arthuri* and the MRCA (most recent common ancestor) of *P. tertius* and *P. coreodes*. (I) Divergence with bidirectional migration between *P. arthuri* and the MRCA of *P. tertius* and *P. coreodes*. (J) Divergence with bidirectional migration between *P. coreodes* and *P. tertius*. (K) Divergence with bidirectional migration between *P. tertius* and *P. arthuri*. (L) divergence with bidirectional migration between *P. tertius* with *P. coreodes* and *P. arthuri*. (M) Divergence with bidirectional migration between *P. coreodes* and *P. arthuri*. (N) divergence with bidirectional migration between *P. coreodes* with *P. tertius* and *P. arthuri*. (O) Divergence with bidirectional migration between *P. arthuri* with *P. coreodes* and *P. tertius*. (P) Divergence with bidirectional migration between the three *Psammolestes* species. Support values for the demographic scenarios are shown under each figure.

**Additional file 19. **Linear correlations of Isolation by distance (IBD) test. (A) 28S (B) CISP (C) CYTB (D) LSM (E) TRNA (F) UPCA (G) PJH.

**Additional file 20. **Mantel’s test for isolation by distance (IBD) and linear correlation results. The result of the Mantel’s test is shown in the two first columns of the table, and the results of the Pearson’s correlation test correspond to the third column. The last two columns show the results of the linear correlation tested between geographical and genetic distances.

**Additional file 21. **Fit of the demographic models tested in.

**Additional file 22. **Individuals of *Psammolestes *species collected in this study.

**Additional file 23. **Genes included in this study, the primers used to obtain their corresponding sequence, and the length of each one of them. “*” symbolizes a new marker used for the delimitation of the Psammolestes species.

**Additional file 24. **GenBank accession numbers of seven loci analyzed in this study and samples origin included in this study.

## Data Availability

All data generated or analyzed during this study are included in this published article [and its additional information files]. The sequences obtained in this study are available under the GenBank accession numbers OM256834-OM256940, OM256942-OM257062.
