# Supplementary figures and images for: Phylogenetic relationships and evolutionary patterns of the genus Psammolestes Bergroth, 1911 (Hemiptera: Reduviidae: Triatominae)
Source: BMC Ecol Evol. 2022 Mar 12;22:30. doi: 10.1186/s12862-022-01987-x (PMC8918316; doi:10.1186/s12862-022-01987-x)

A

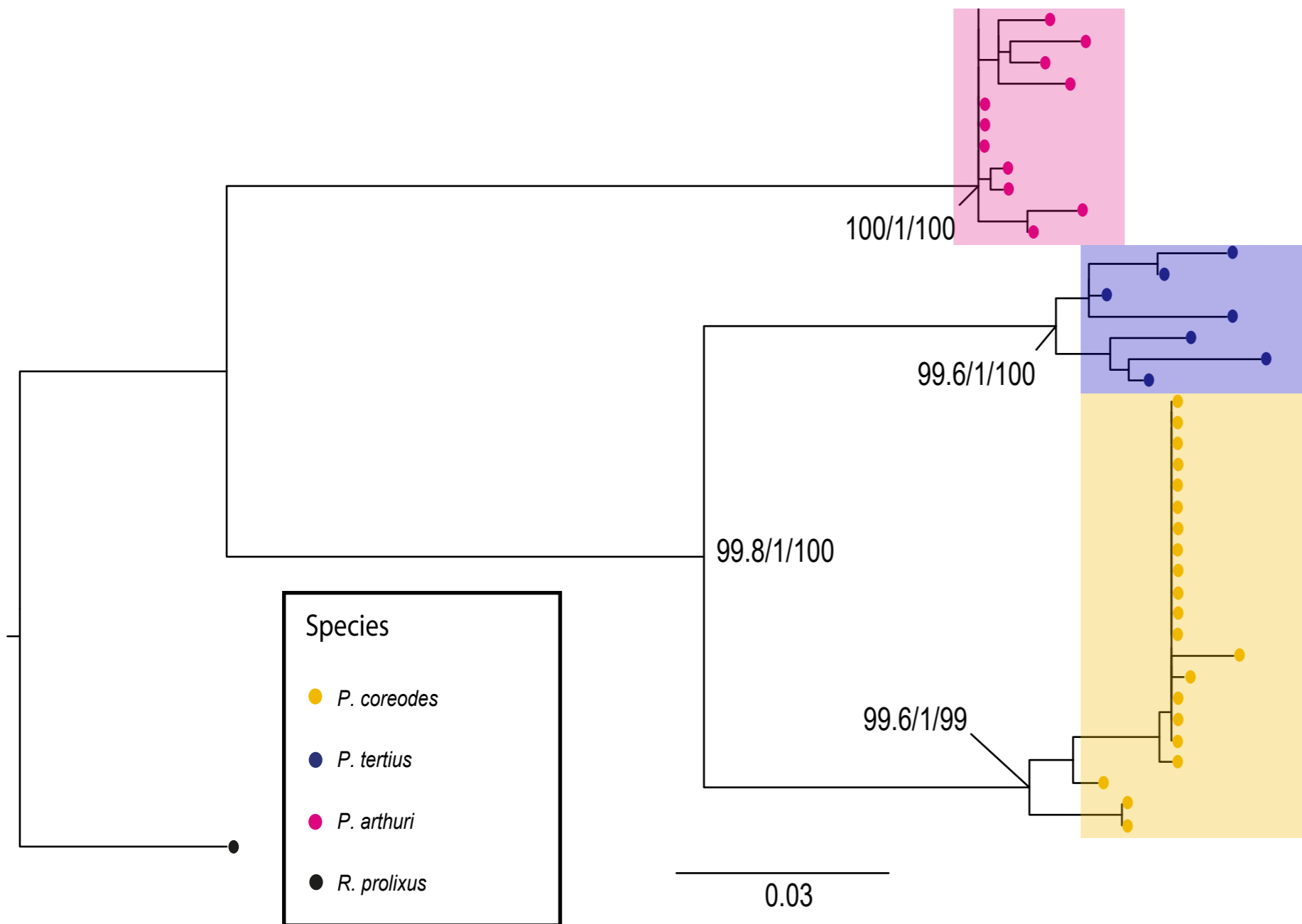

B

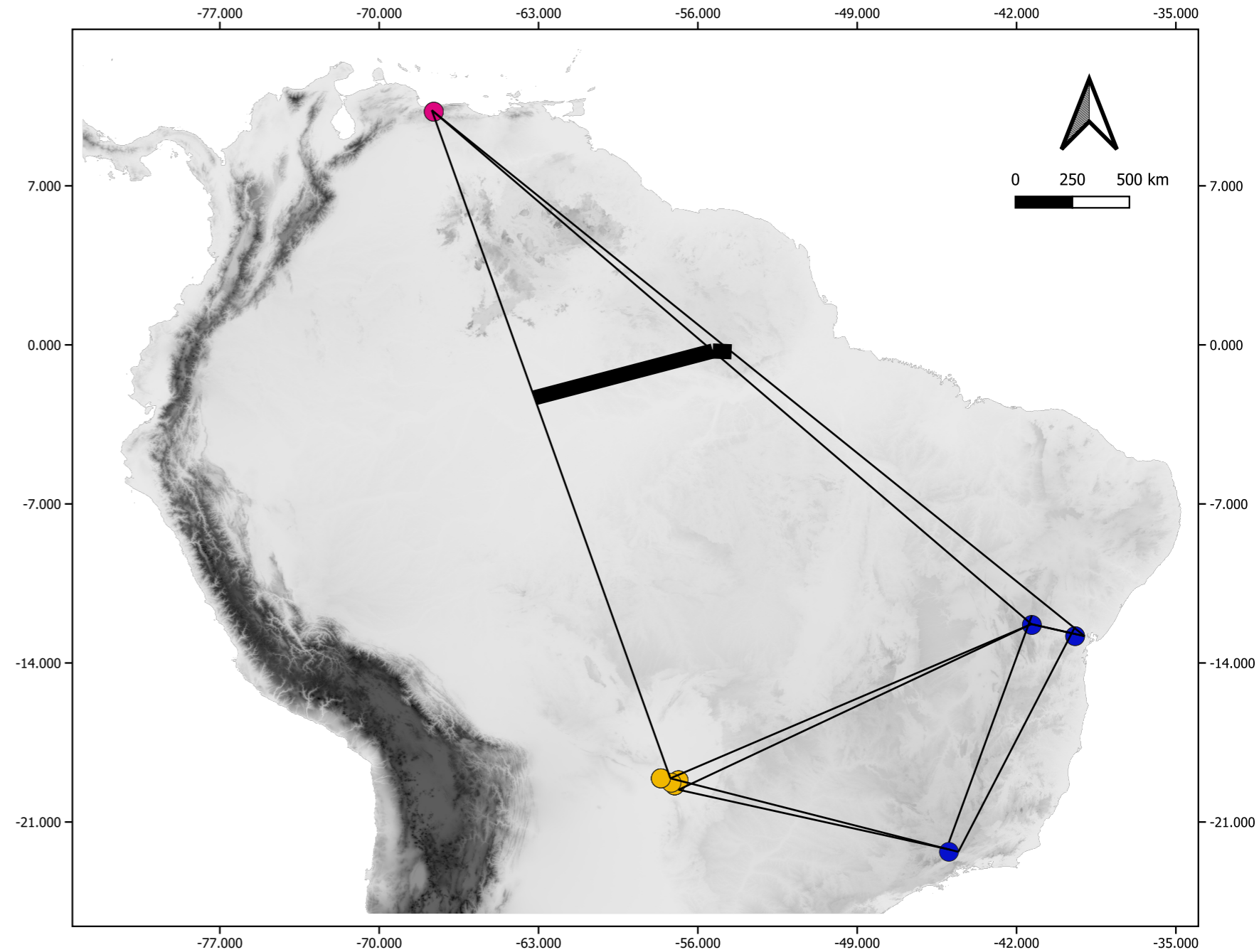

Supplement: Supplementary file 1 — Additional file 1. CYTB Phylogenetic reconstruction and Barrier test. (A) Phylogenetic reconstruction with the ML algorithm based on the mitochondrial marker CYTB (B) Barrier test algorithm based on molecular and geographical arrays. Bootstrap values on the internal nodes are shown in the following order: SH-aLRT/aBayes/ultrafast bootstrap support. Only nodes with bootstrap values higher than 60 are shown. [file 12862_2022_1987_MOESM1_ESM.pdf]

A

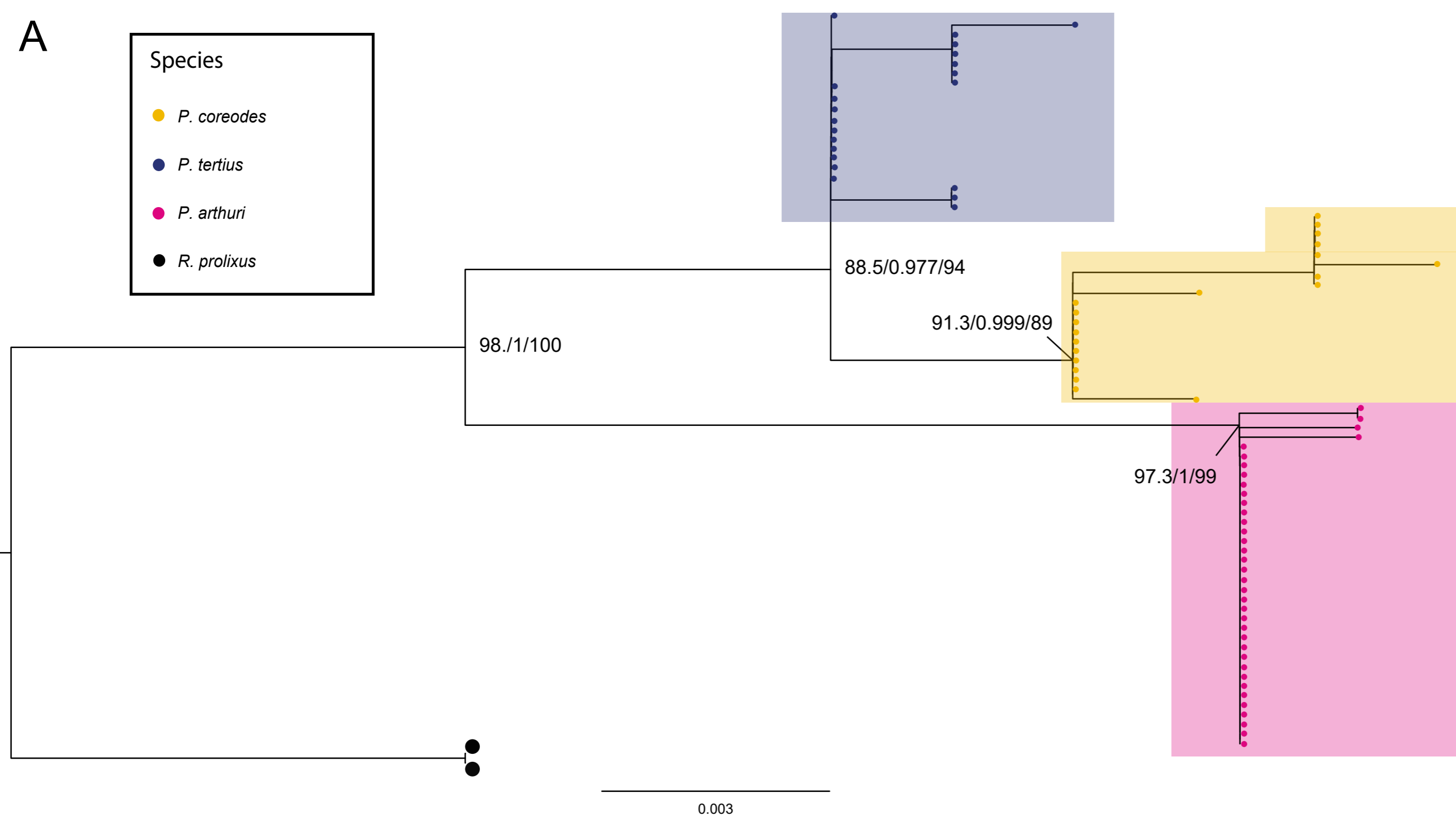

B

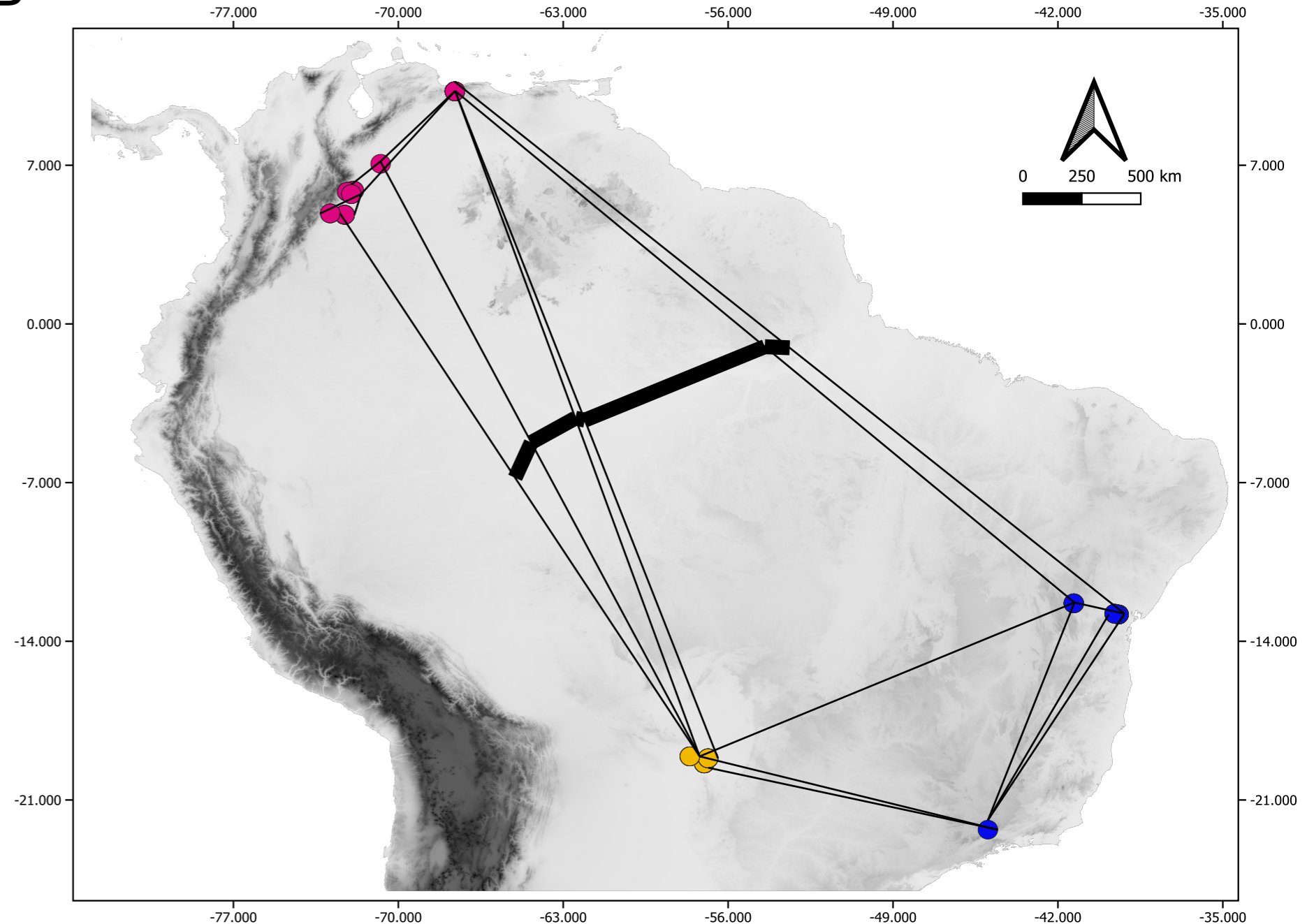

Supplement: Supplementary file 2 — Additional file 2. PJH Phylogenetic reconstruction and Barrier test (A) Phylogenetic reconstruction with the ML algorithm based on the nuclear marker PJH (B) Barrier test algorithm based on molecular and geographical arrays. Bootstrap values on the internal nodes are shown in the following order: SH-aLRT/aBayes/ultrafast bootstrap support. Only nodes with bootstrap values higher than 60 are shown. [file 12862_2022_1987_MOESM2_ESM.pdf]

A

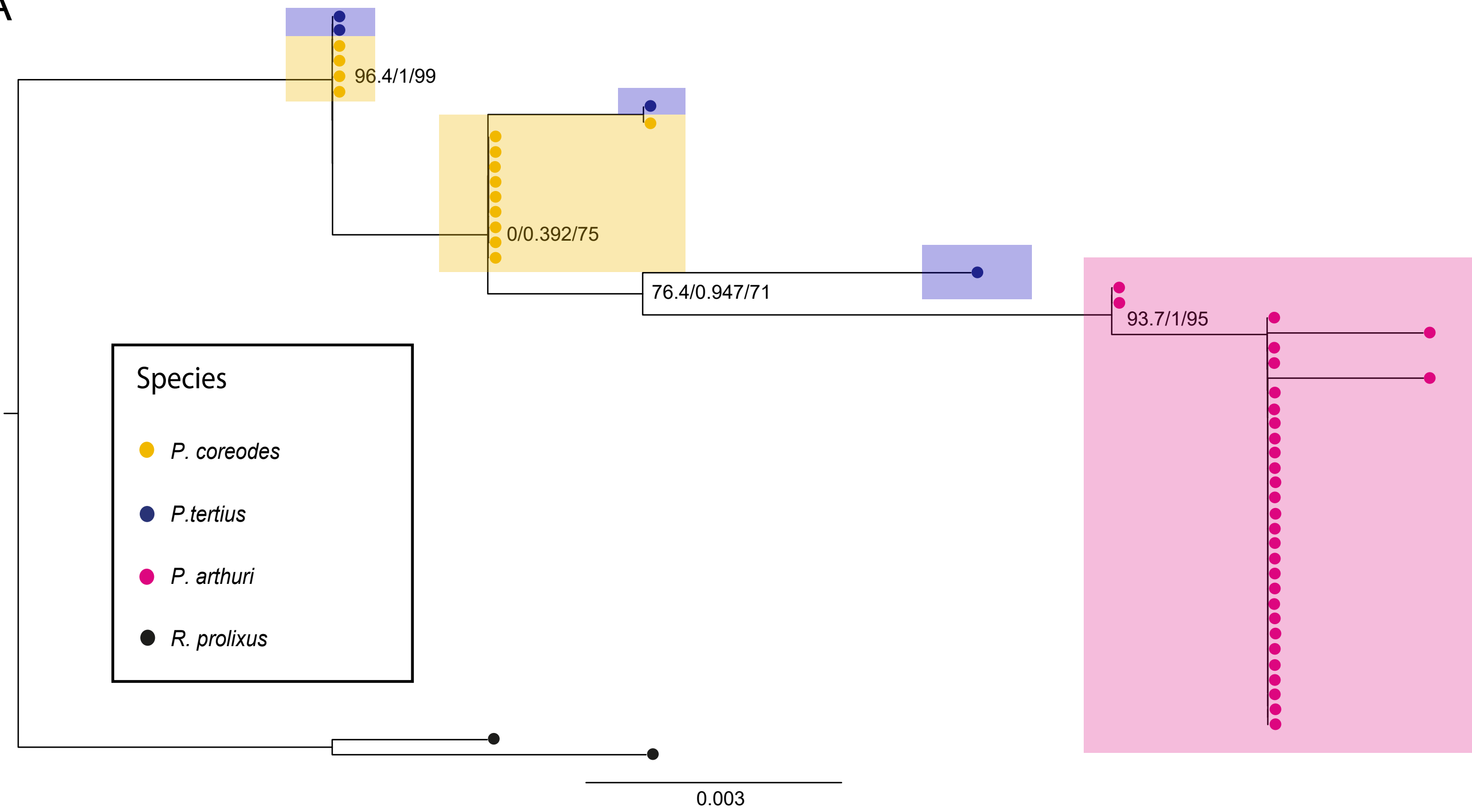

B

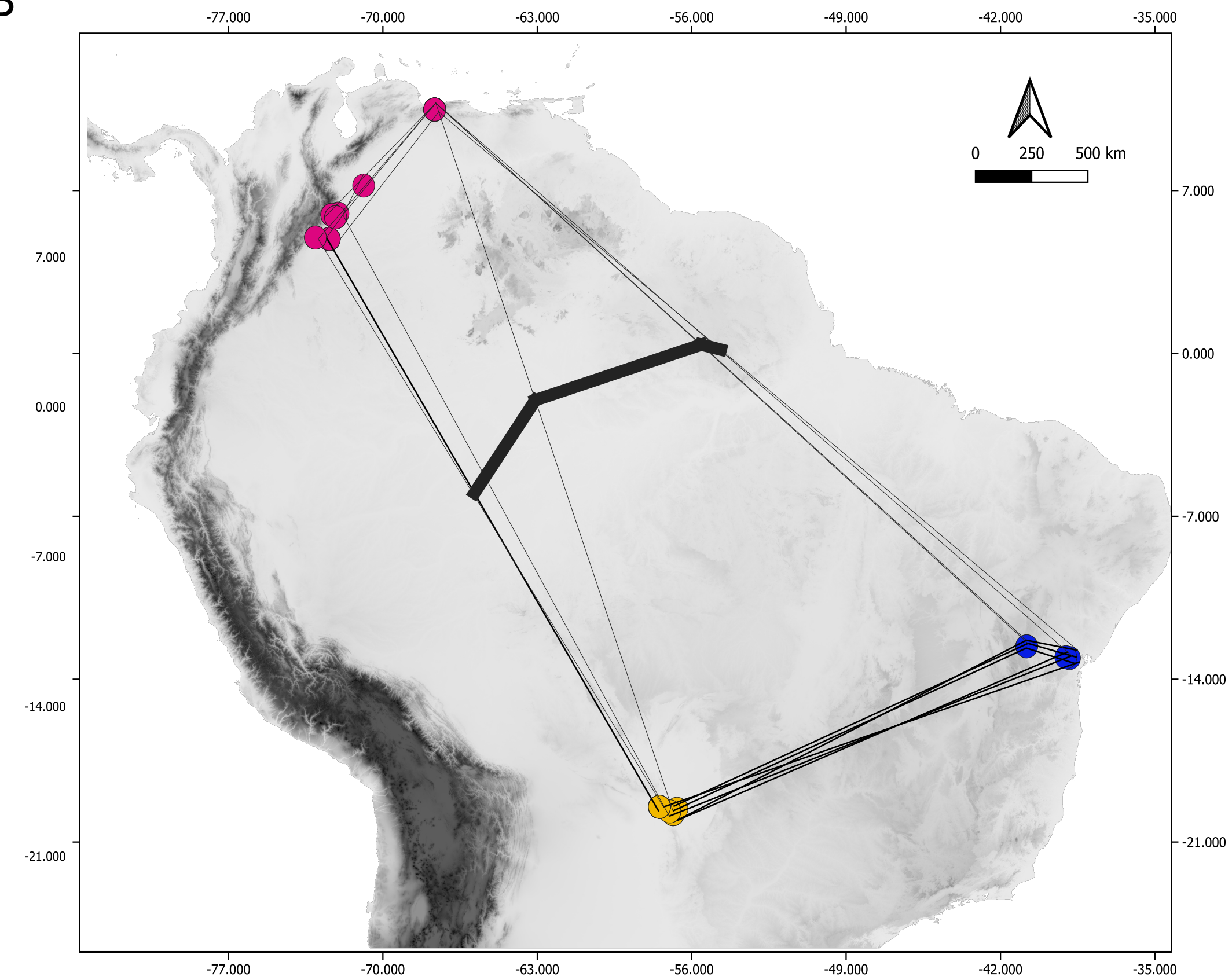

Supplement: Supplementary file 3 — Additional file 3. 28S Phylogenetic reconstruction and Barrier test (A) Phylogenetic reconstruction with the ML algorithm based on the nuclear marker 28S (B) Barrier test algorithm based on molecular and geographical arrays (B). Bootstrap values on the internal nodes are shown in the following order: SH-aLRT/aBayes/ultrafast bootstrap support. Only nodes with bootstrap values higher than 60 are shown. [file 12862_2022_1987_MOESM3_ESM.pdf]

A

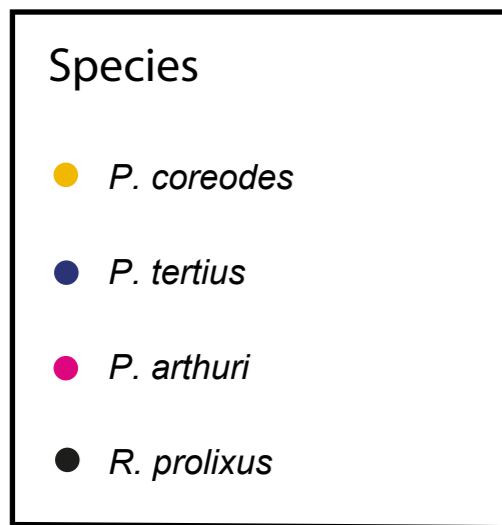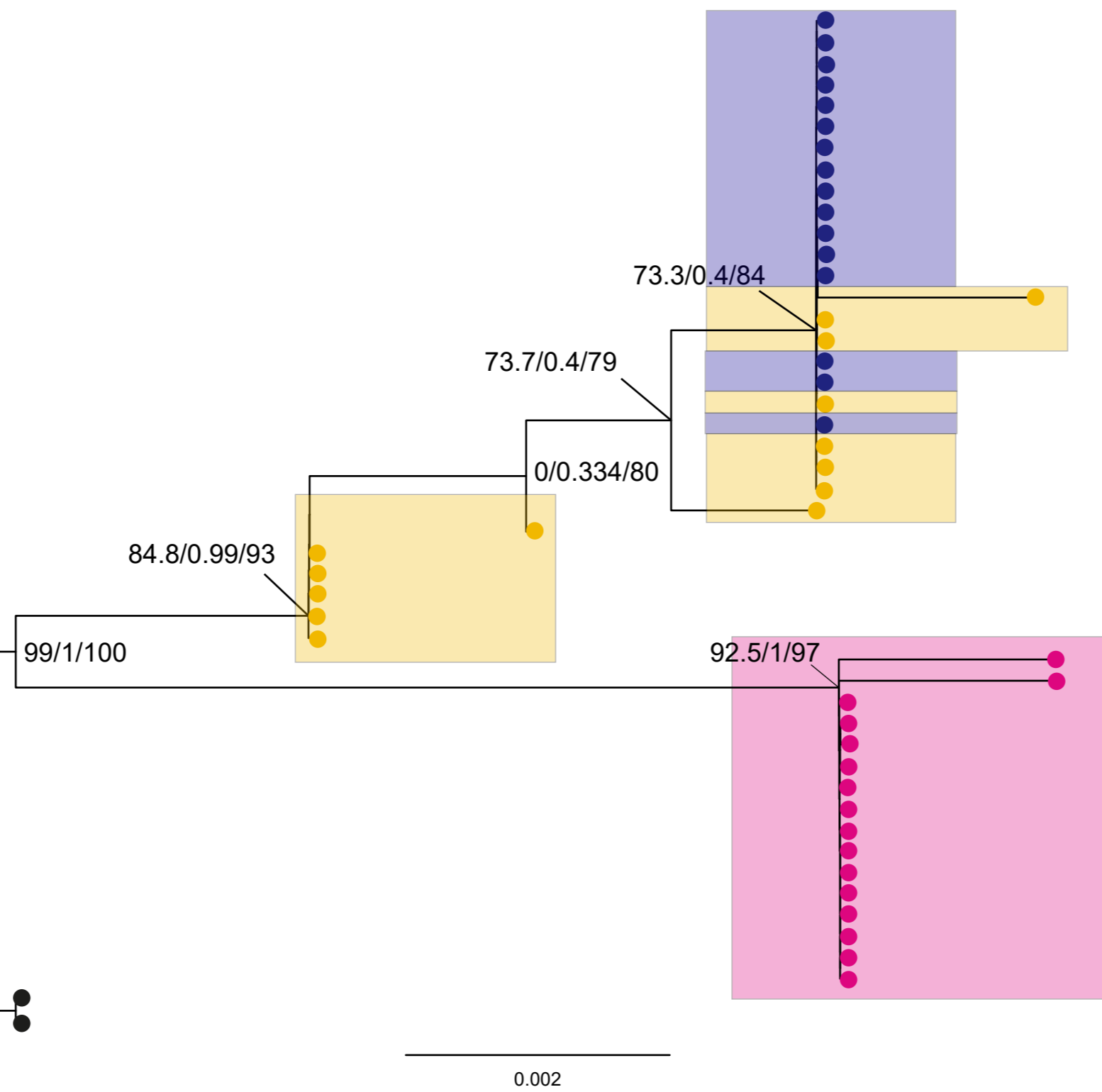

B

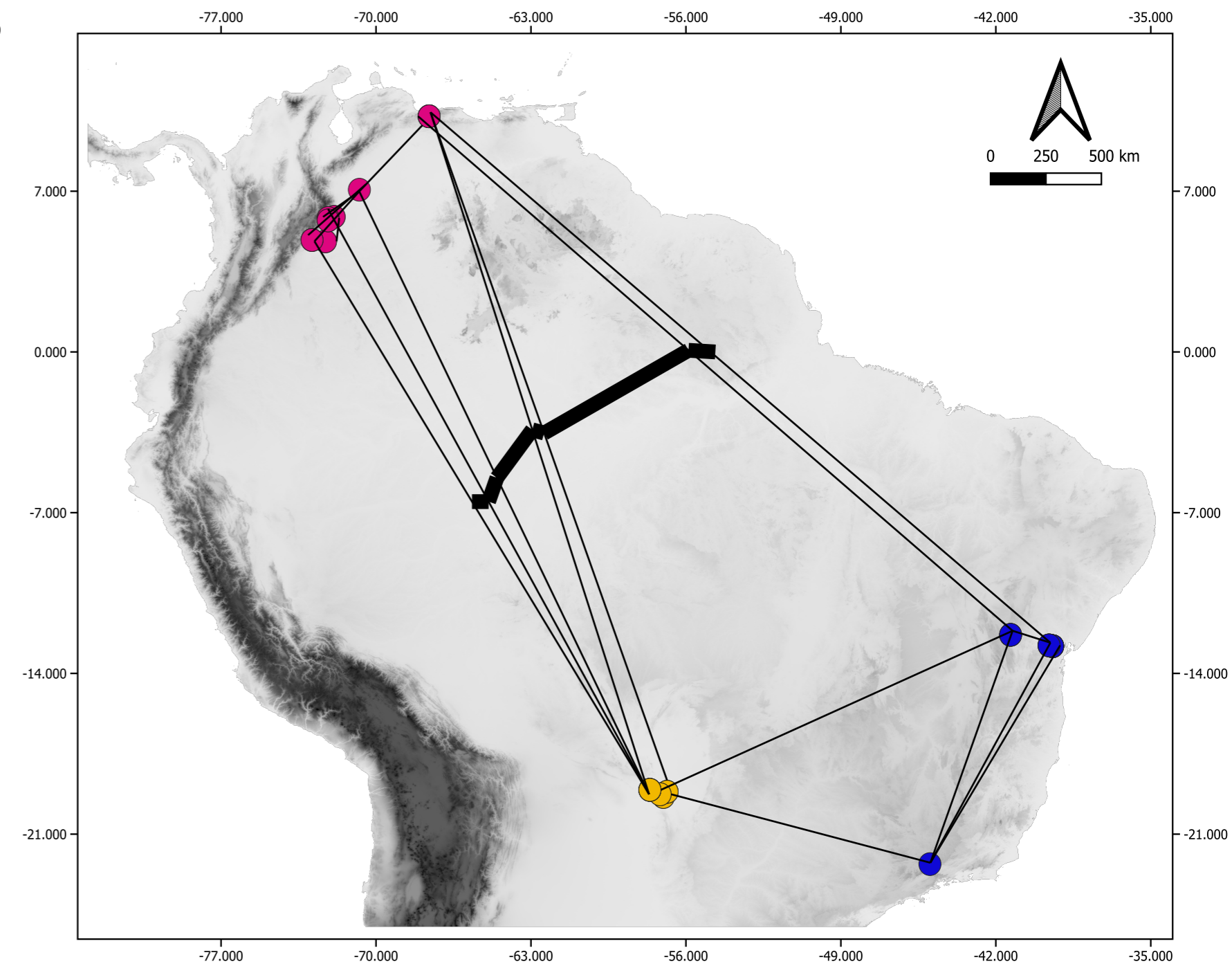

Supplement: Supplementary file 4 — Additional file 4. CISP Phylogenetic reconstruction and Barrier test (A) Phylogenetic reconstruction with the ML algorithm based on the nuclear marker CISP (B) Barrier test algorithm based on molecular and geographical arrays. Bootstrap values on the internal nodes are shown in the following order: SH-aLRT/aBayes/ultrafast bootstrap support. Only nodes with bootstrap values higher than 60 are shown. [file 12862_2022_1987_MOESM4_ESM.pdf]

A

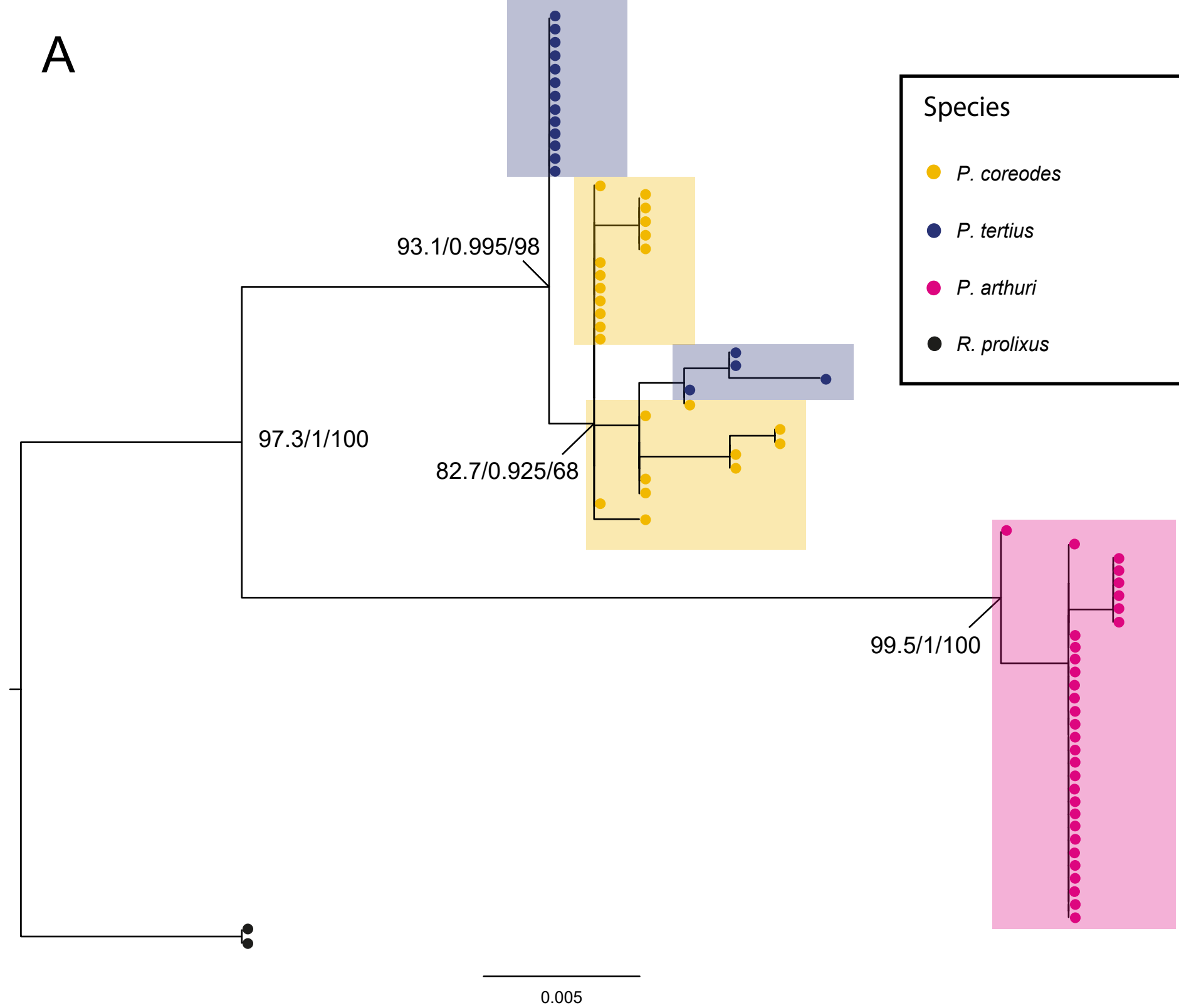

B

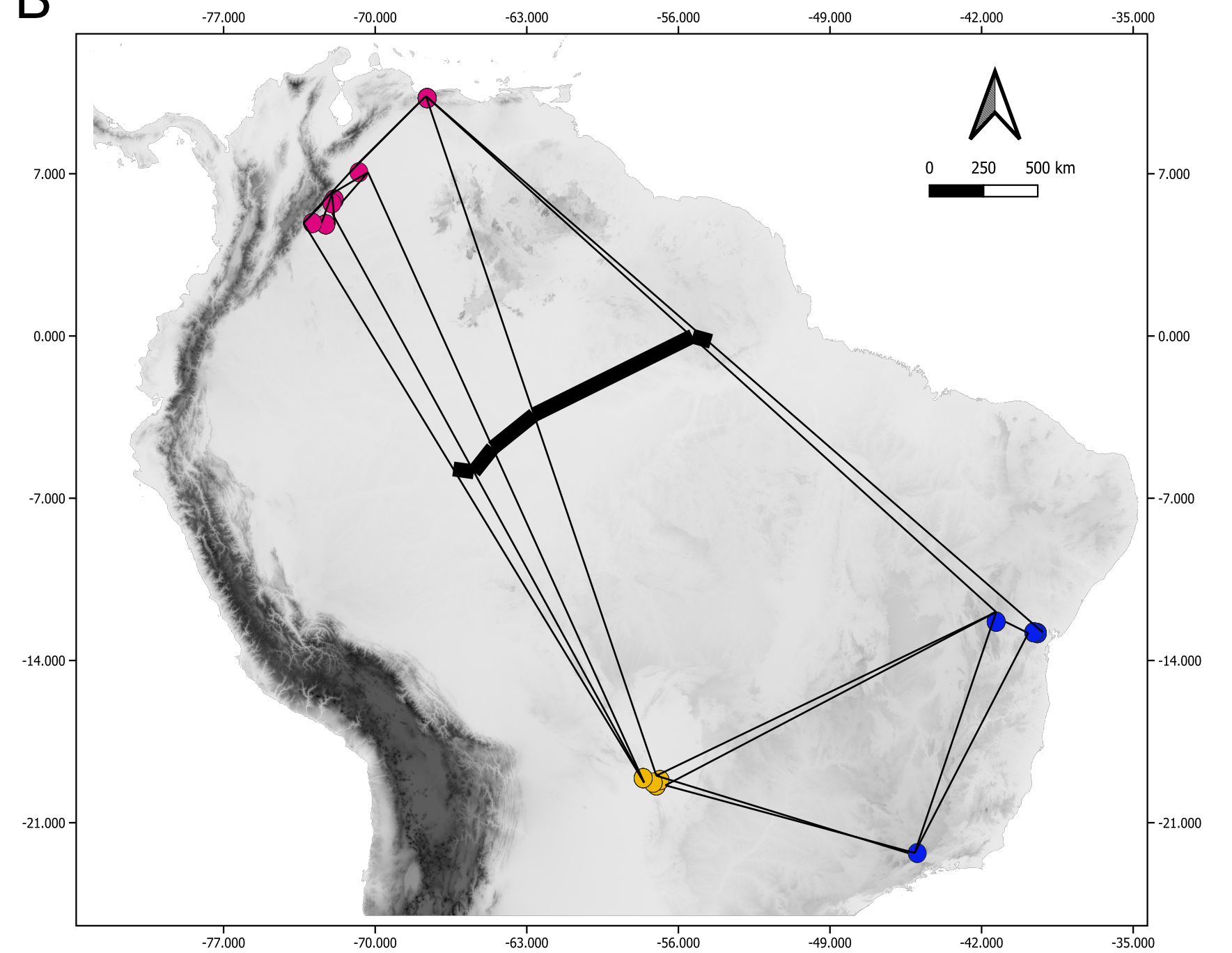

Supplement: Supplementary file 5 — Additional file 5. LSM Phylogenetic reconstruction and Barrier test. (A) Phylogenetic reconstruction wit the ML algorithm based on the nuclear marker LSM (B) Barrier test algorithm based on molecular and geographical arrays. Bootstrap values on the internal nodes are shown in the following order: SH-aLRT/aBayes/ultrafast bootstrap support. Only nodes with bootstrap values higher than 60 are shown. [file 12862_2022_1987_MOESM5_ESM.pdf]

A

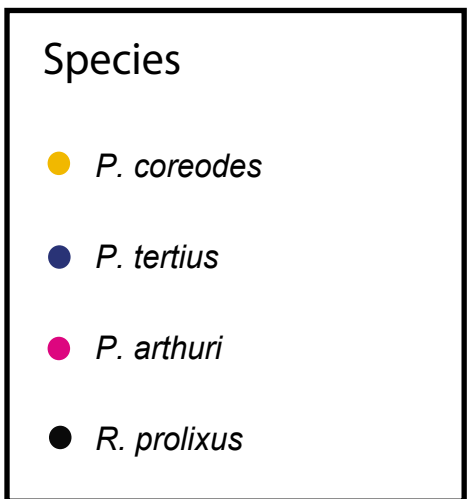

92.2/1/98

100/1/100

99.6/1/100

0.006

B

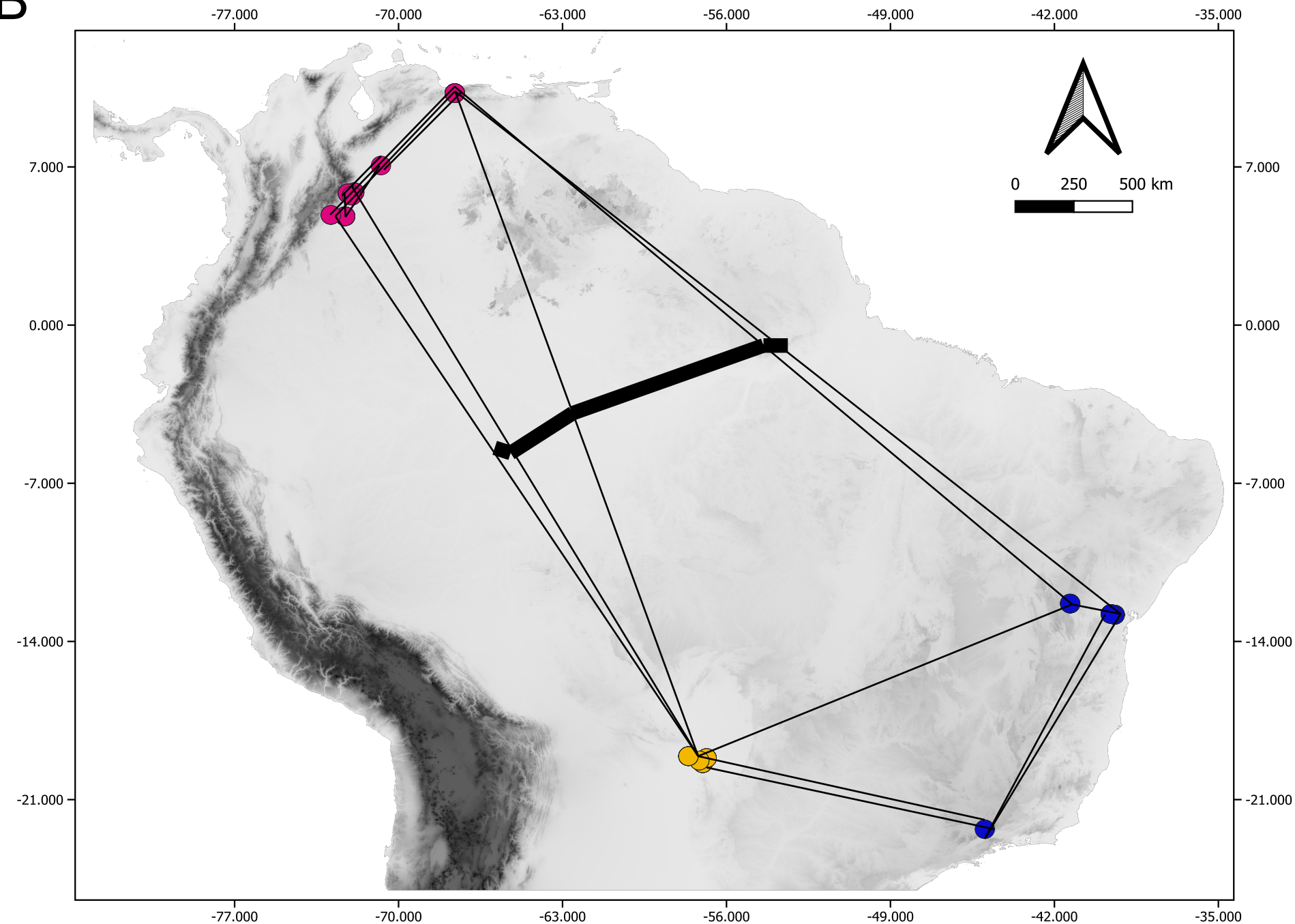

Supplement: Supplementary file 6 — Additional file 6. TRNA Phylogenetic reconstruction and Barrier test. (A) Phylogenetic reconstruction with the ML algorithm based on the nuclear marker TRNA (B) Barrier test algorithm based on molecular and geographical arrays. Bootstrap values on the internal nodes are shown in the following order: SH-aLRT/aBayes/ultrafast bootstrap support. Only nodes with bootstrap values higher than 60 are shown. [file 12862_2022_1987_MOESM6_ESM.pdf]

A

Species

● *P. coreodes*

● *P. tertius*

● *P. arthuri*

● *R. prolixus*

99.9/1/100

99/1/100

0.007

75.6/0.753/78

100/1/100

87.4/0.992/78

85.3/0.997/83

B

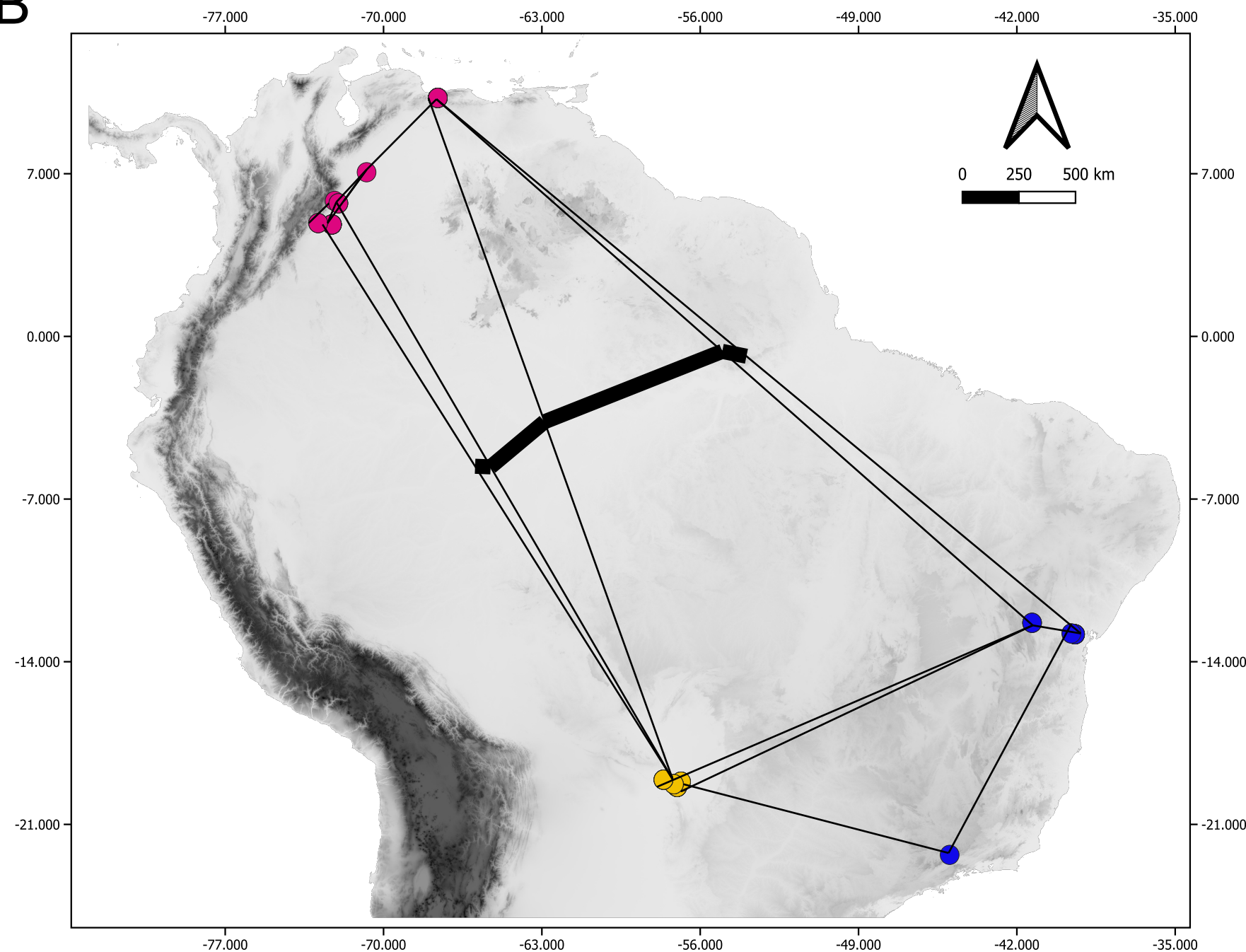

Supplement: Supplementary file 7 — Additional file 7. UPCA Phylogenetic reconstruction and Barrier test. (A) Phylogenetic reconstruction with the ML algorithm based on the nuclear marker UPCA (B) Barrier test algorithm based on molecular and geographical arrays. Bootstrap values on the internal nodes are shown in the following order: SH-aLRT/aBayes/ultrafast bootstrap support. Only nodes with bootstrap values higher than 60 are shown. [file 12862_2022_1987_MOESM7_ESM.pdf]

A.

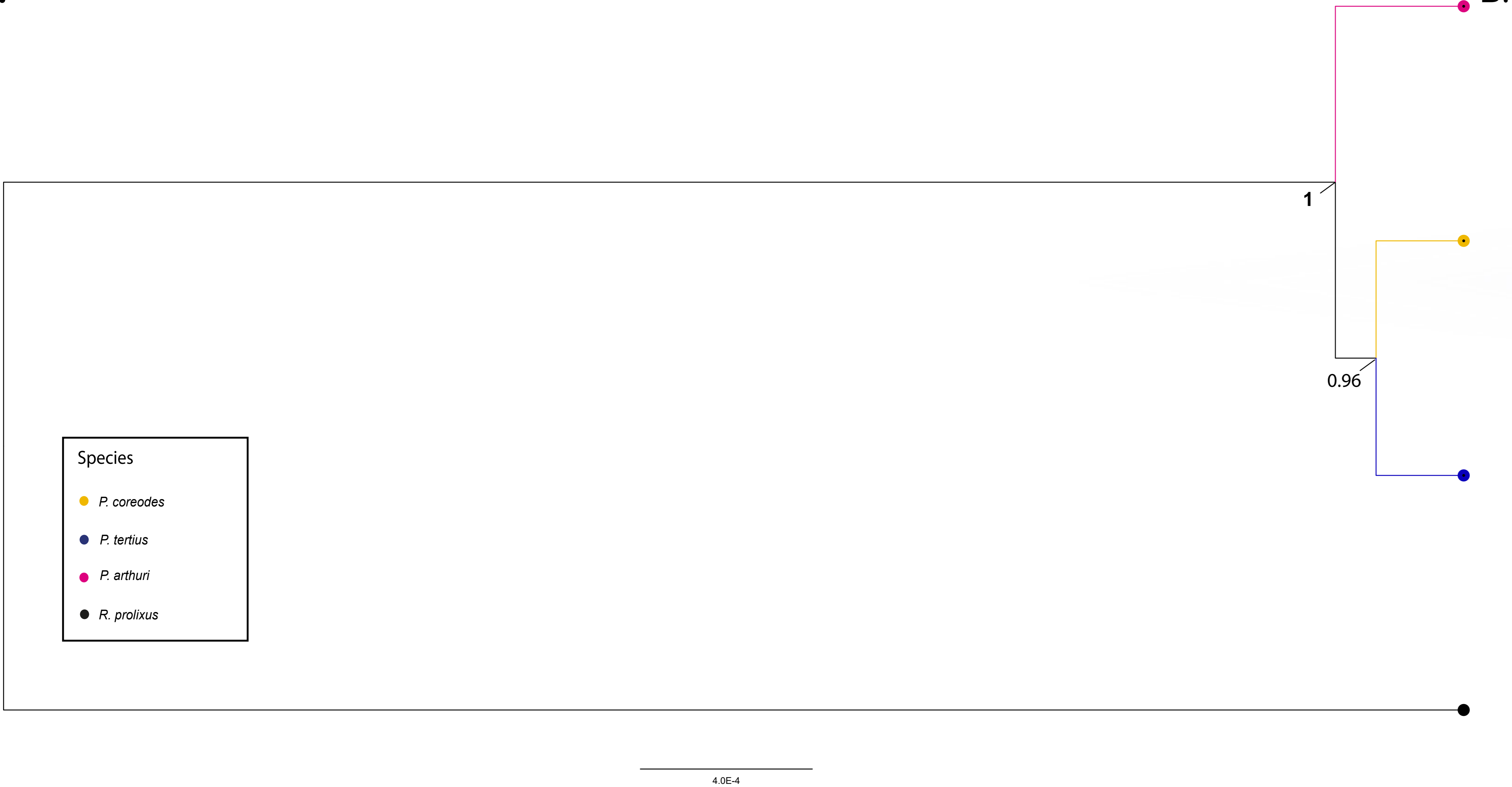

B.

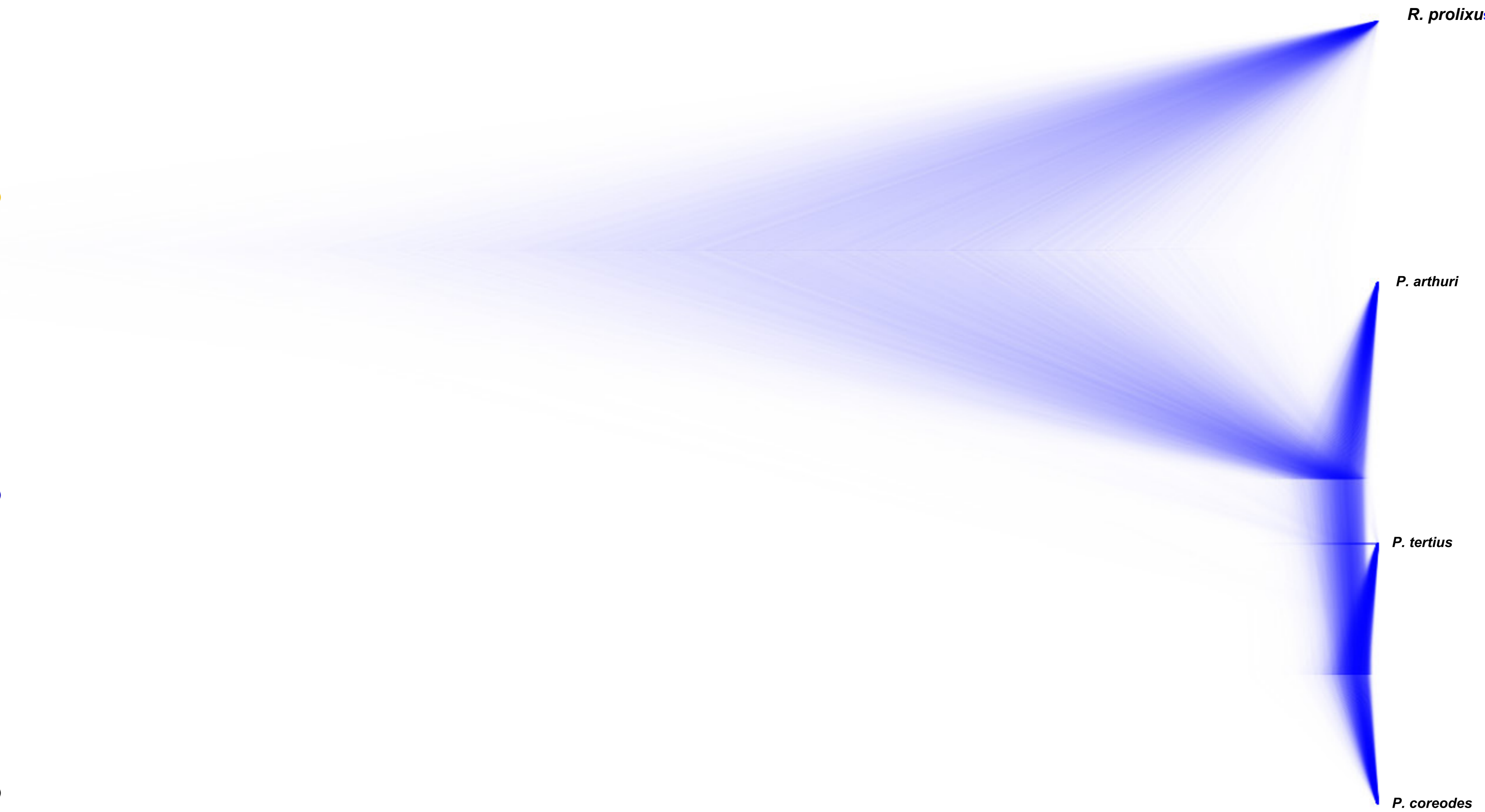

Supplement: Supplementary file 8 — Additional file 8. Bayesian inference of species tree based on multilocus data (A) Maximum clade credibility tree based on Bayesian inference of the seven genes used in this study. The values observed represent posterior probabilities. (B) Bayesian species tree from multilocus data. [file 12862_2022_1987_MOESM8_ESM.pdf]

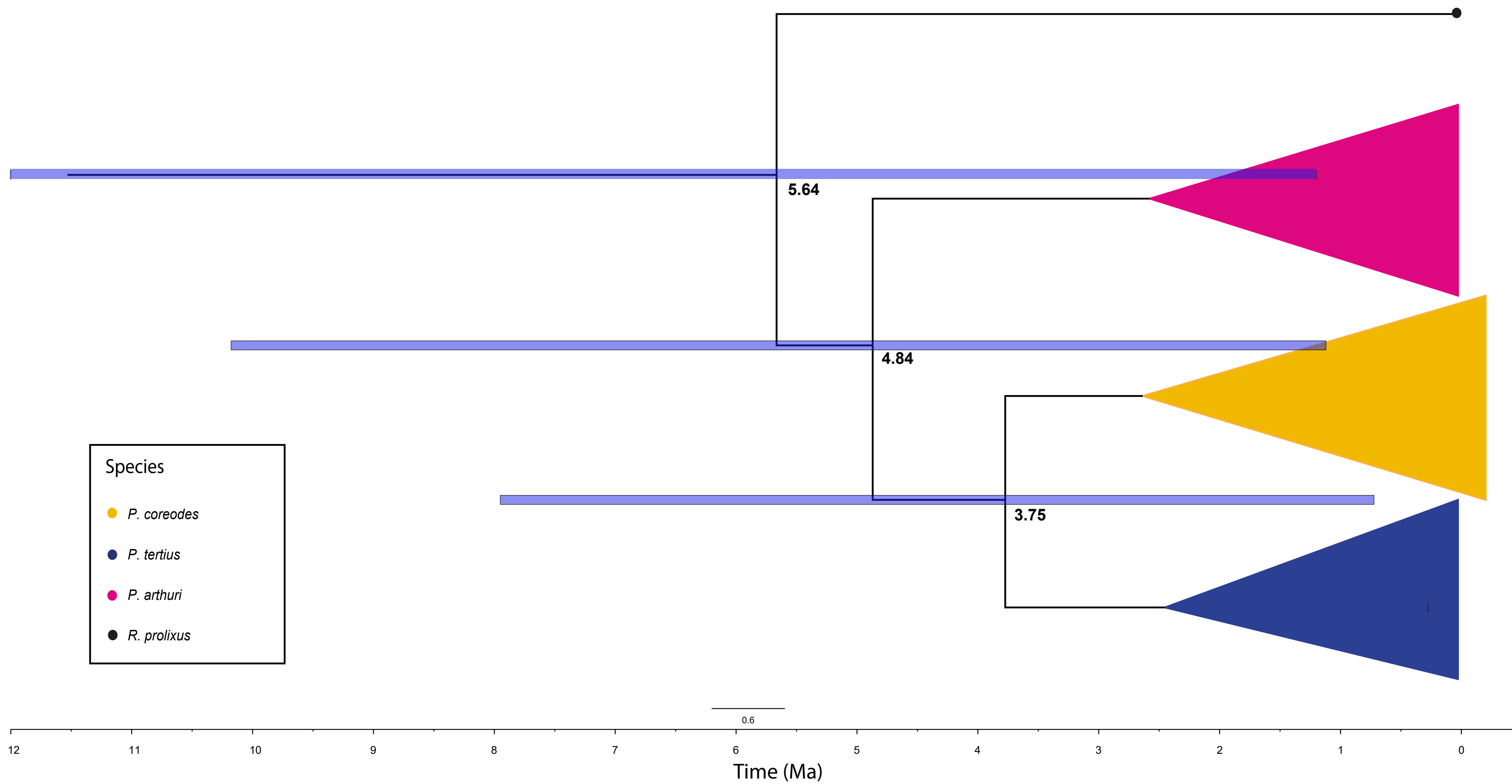

Supplement: Supplementary file 9 — Additional file 9. Bayesian inference phylogenetics tree for the locus CYTB obtained in *BEAST. Horizontal purple bars illustrate the 95% HPD for the nodes’ divergence time. Branch with a posterior probability above 0.95 are show [file 12862_2022_1987_MOESM9_ESM.pdf]

774\_Rpro  
775\_Rpro

0.0047682

1.00

1.00

1.00

Species

- *P. coreodes*
- *P. tertius*
- *P. arthuri*
- *R. prolixus*

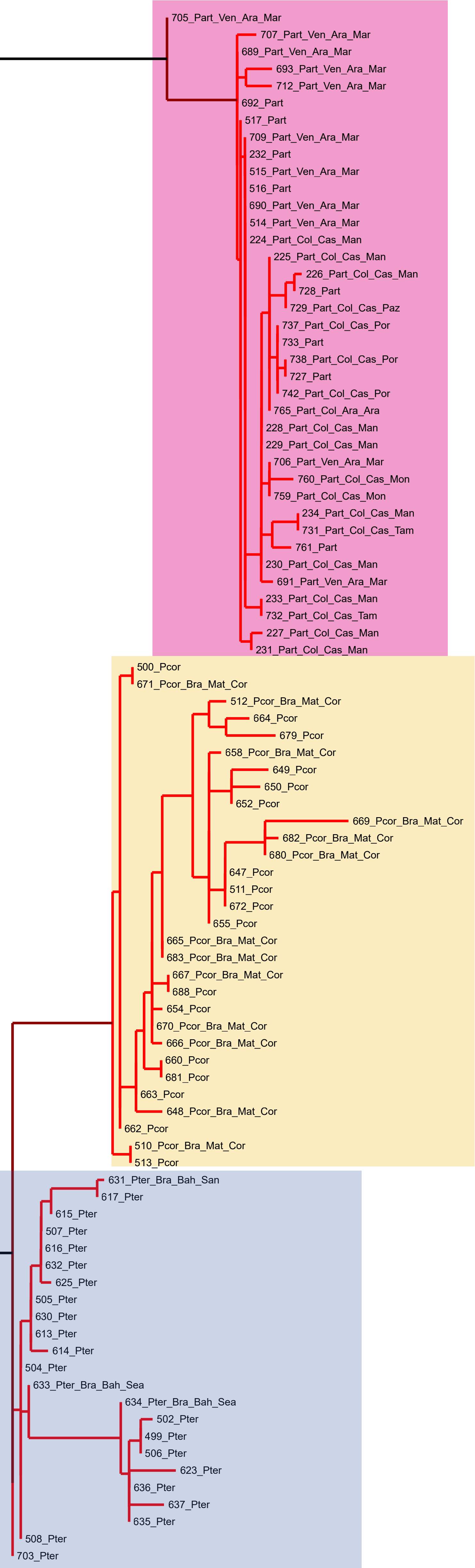

Supplement: Supplementary file 10 — Additional file 10. Posterior probabilities on nodes, calculated by the mPTP algorithm. [file 12862_2022_1987_MOESM10_ESM.pdf]

A

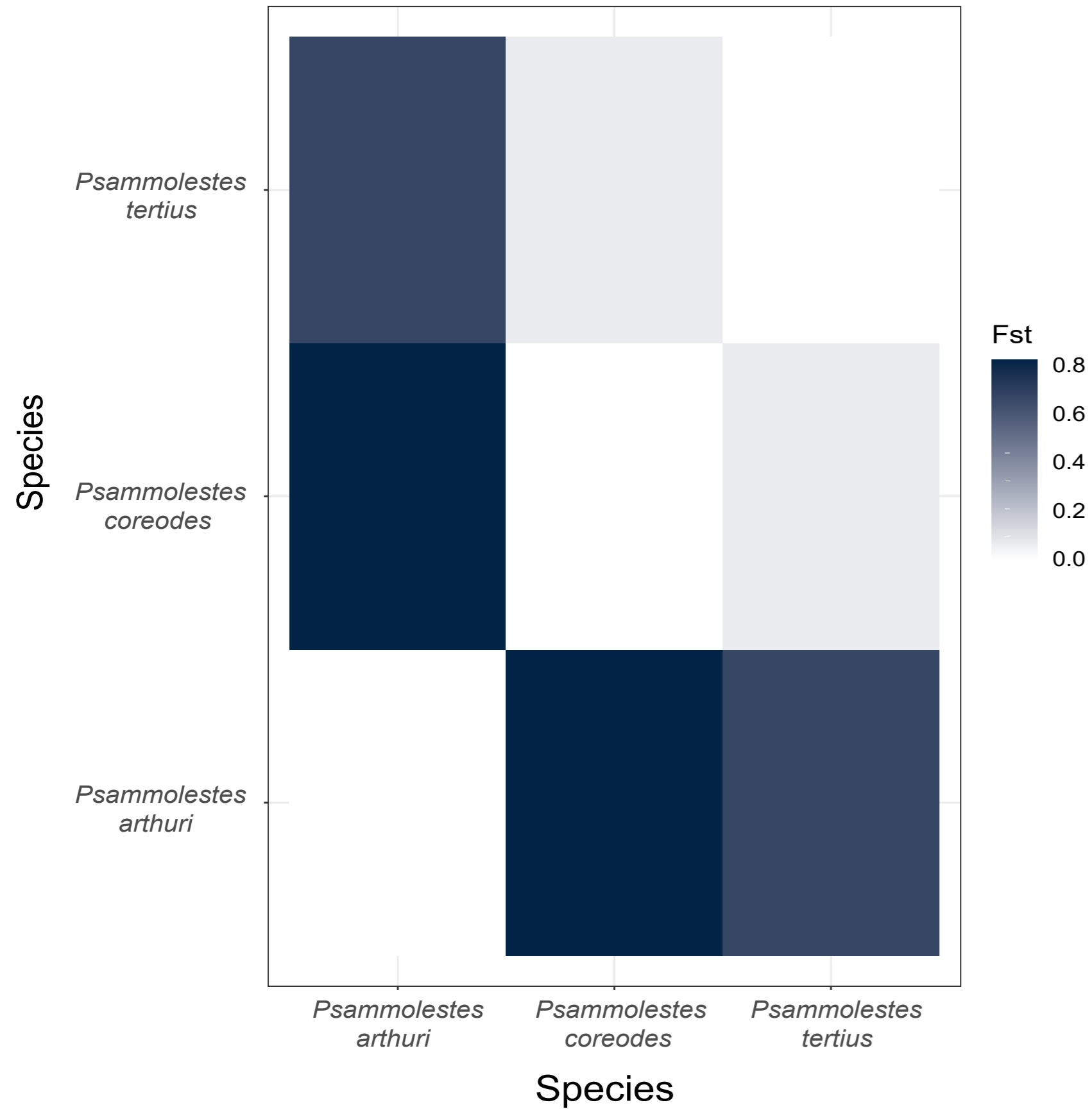

B

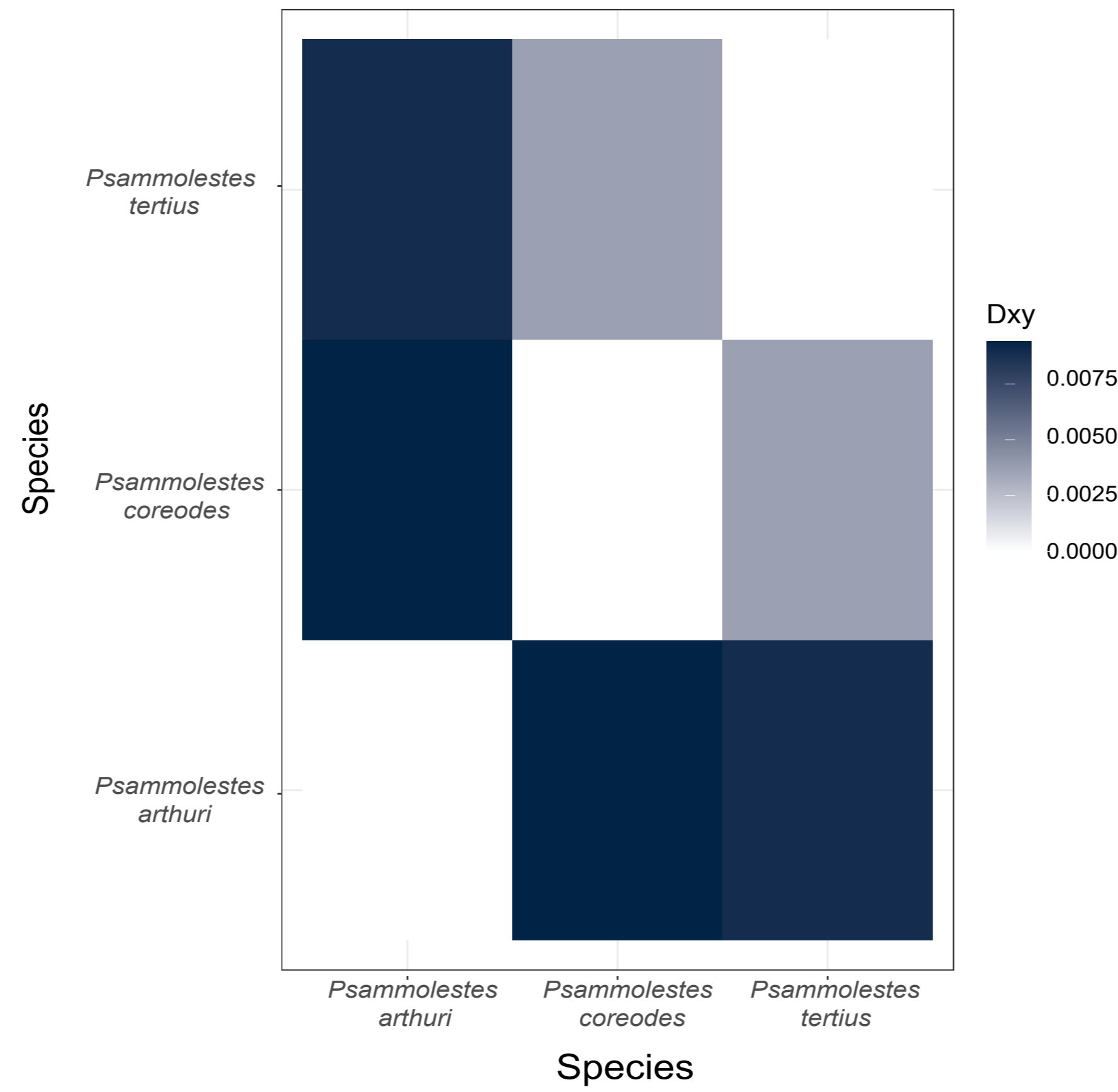

C

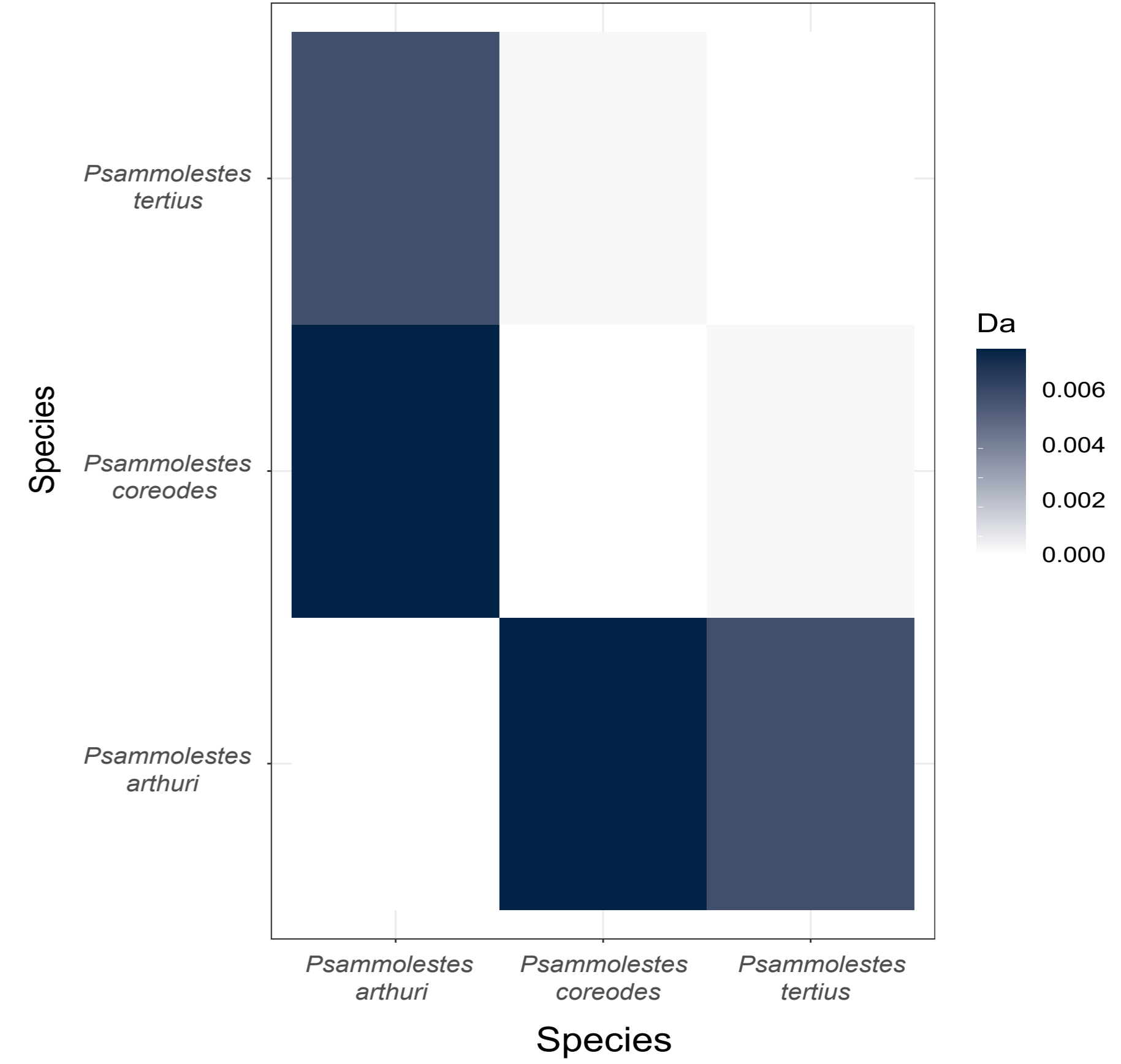

Supplement: Supplementary file 11 — Additional file 11. Heatmaps calculated for three different statistics: A) Fst, B) Dxy and C) Da for three species based on the molecular data obtained from the nuclear marker 28S. [file 12862_2022_1987_MOESM11_ESM.pdf]

A

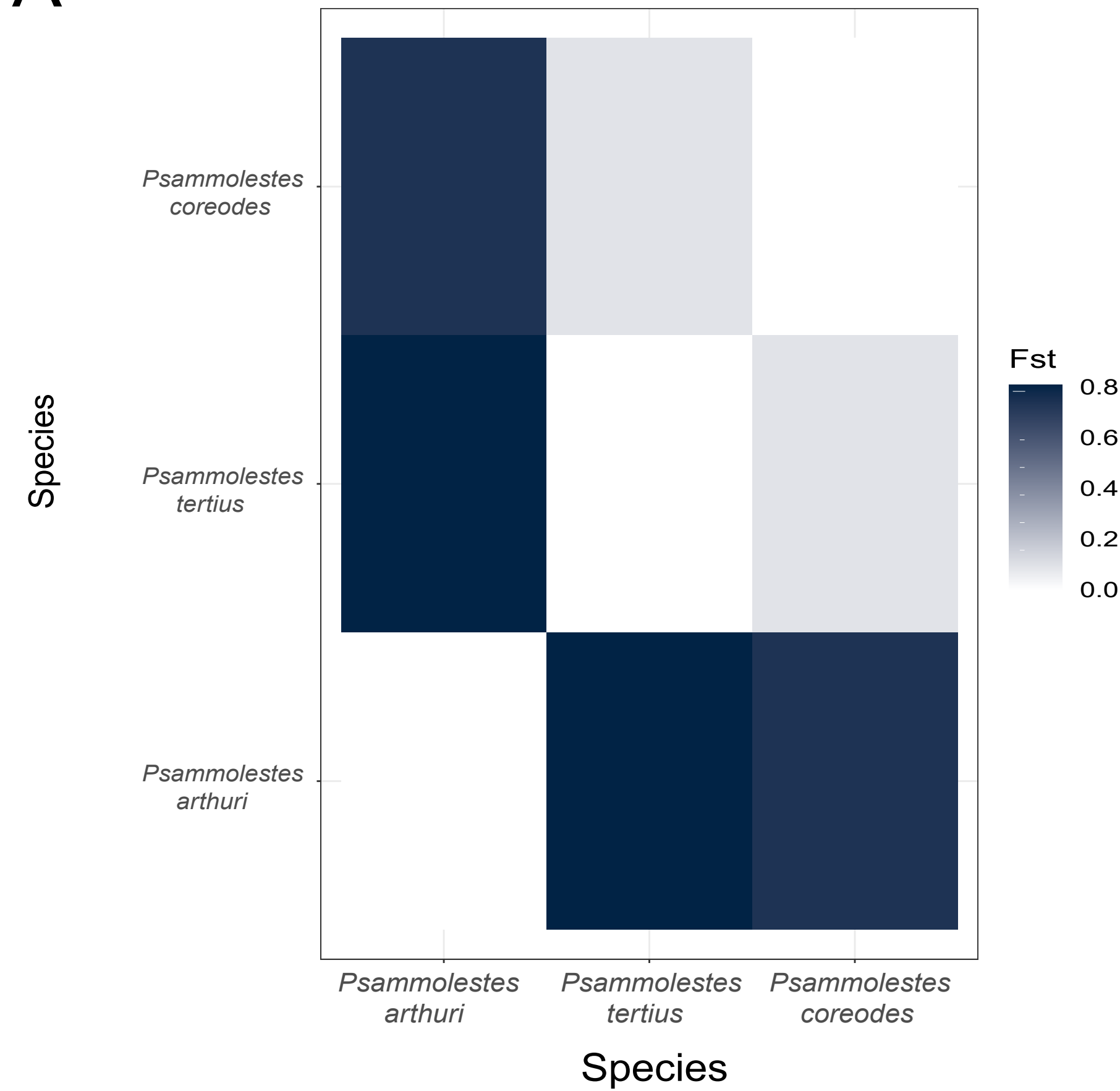

B

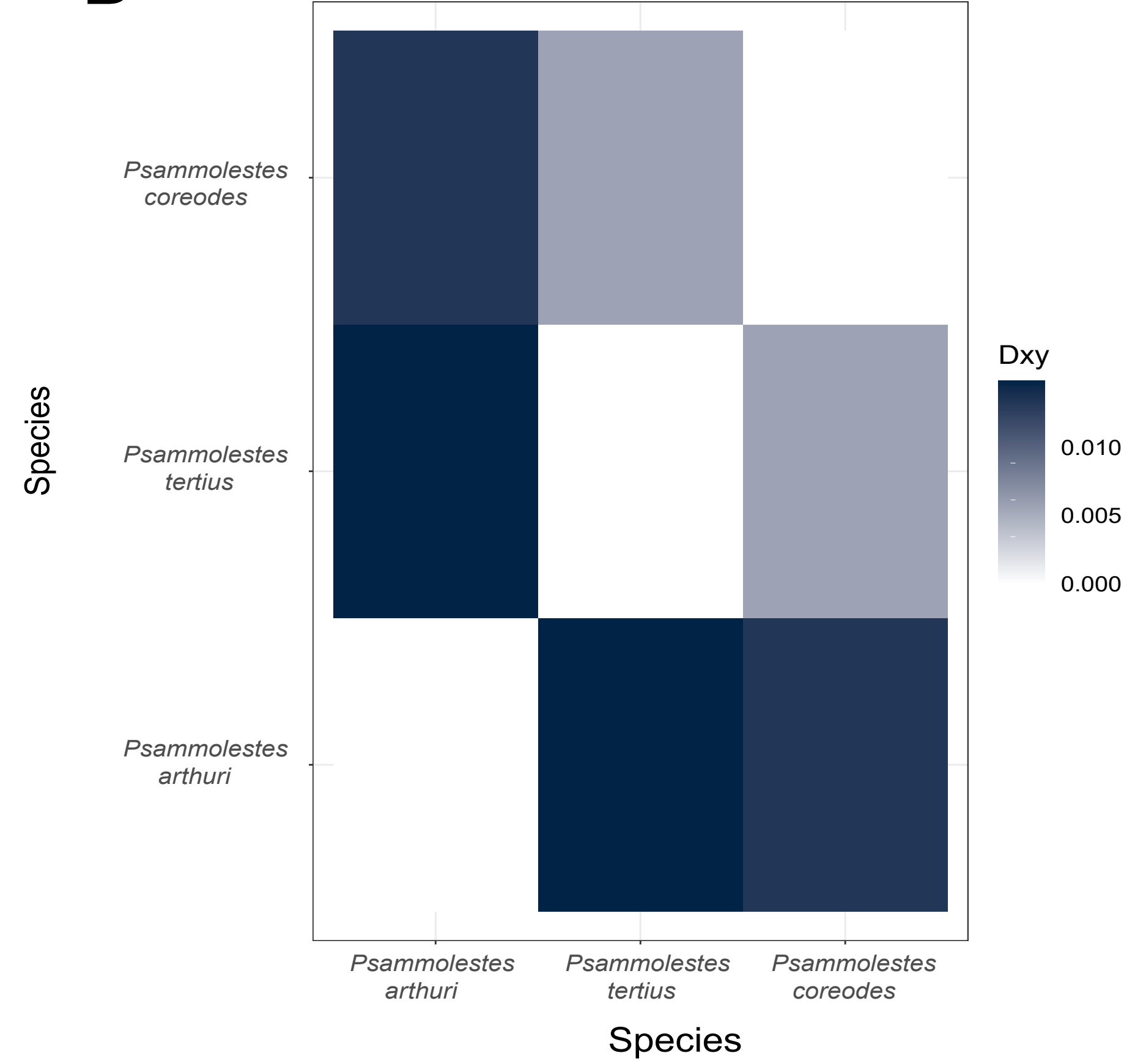

C

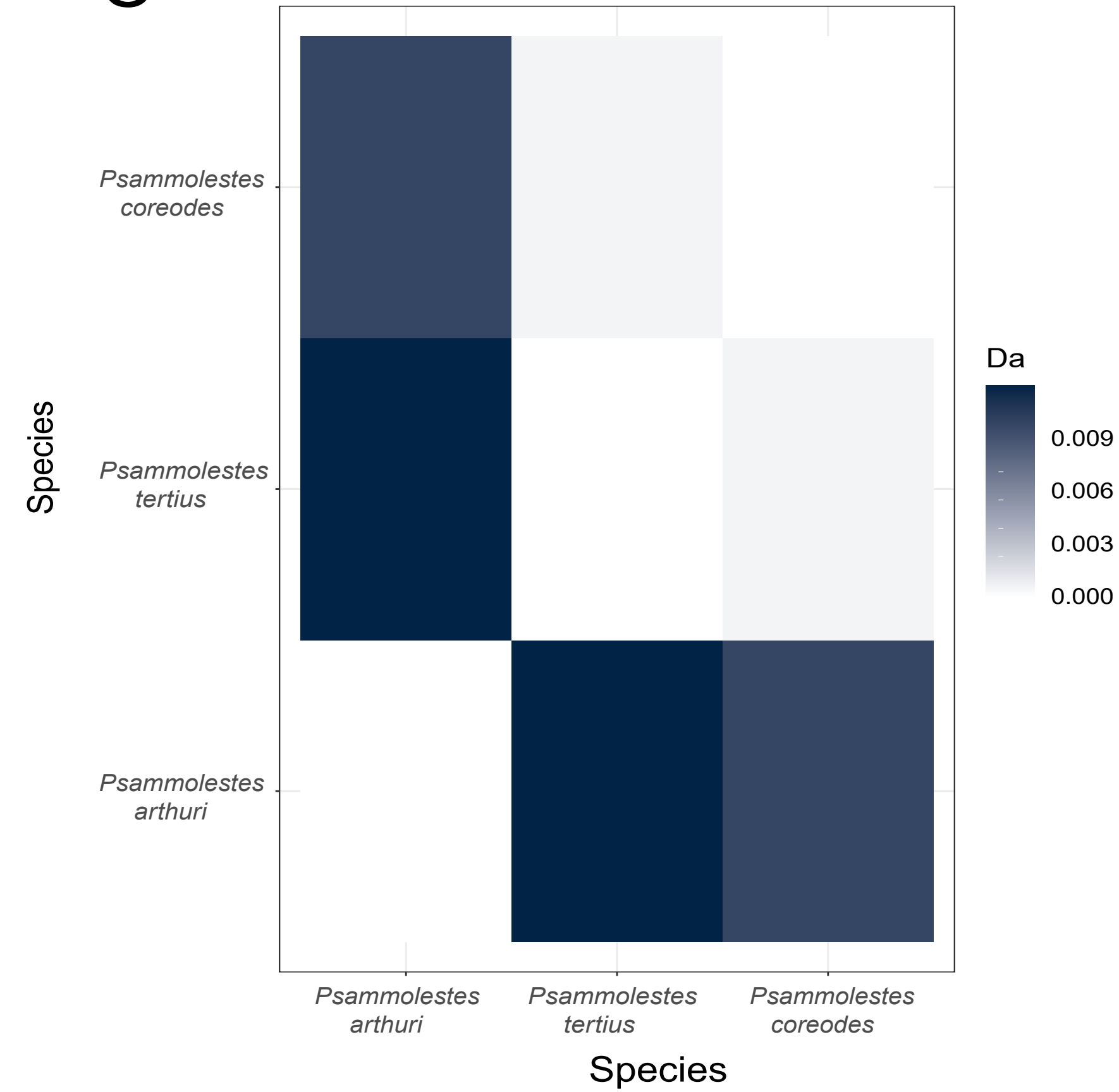

Supplement: Supplementary file 12 — Additional file 12. Heatmaps calculated for three different statistics: A) Fst, B) Dxy and C) Da for three species based on the molecular data obtained from the nuclear marker CISP. [file 12862_2022_1987_MOESM12_ESM.pdf]

A

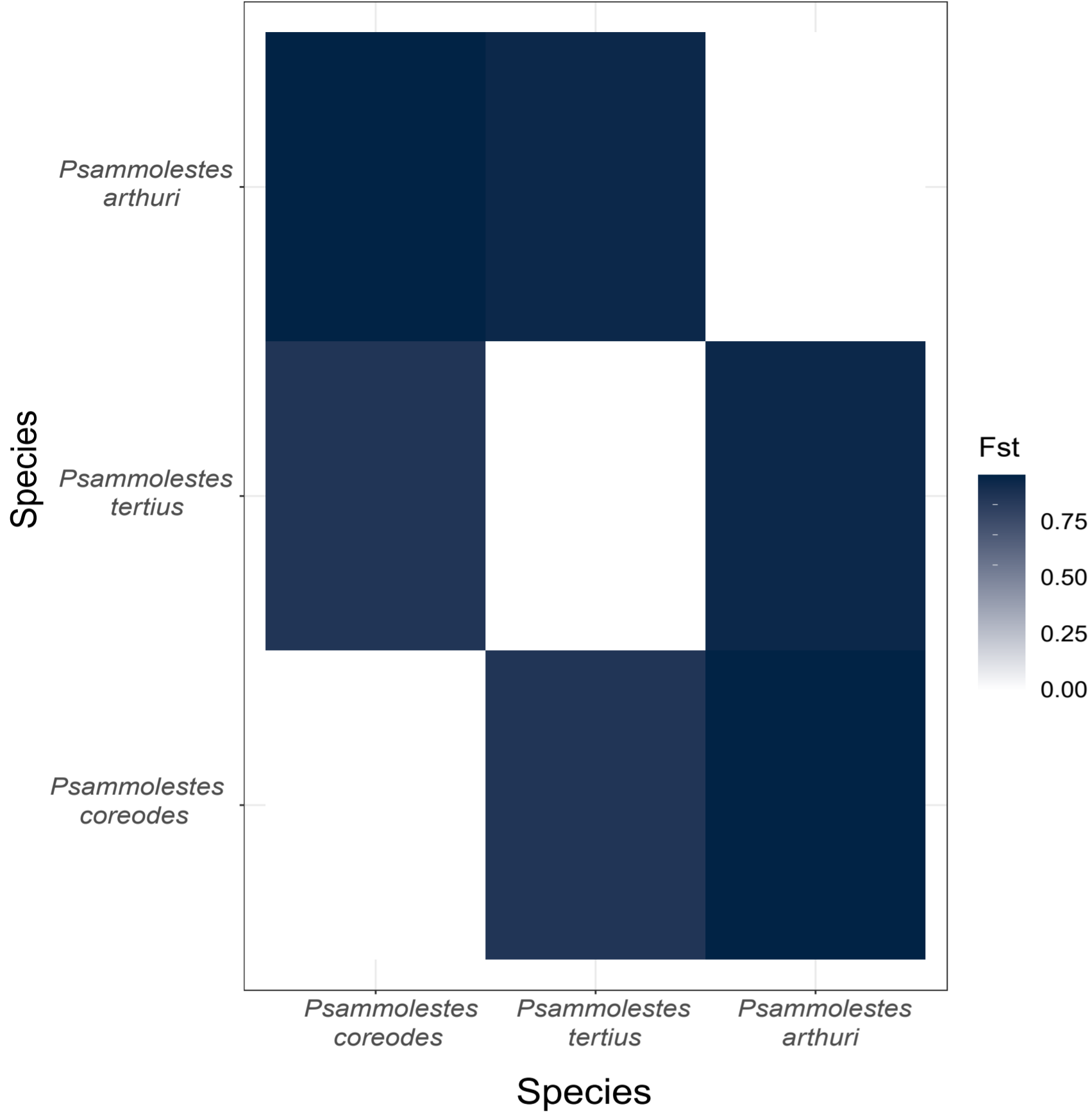

B

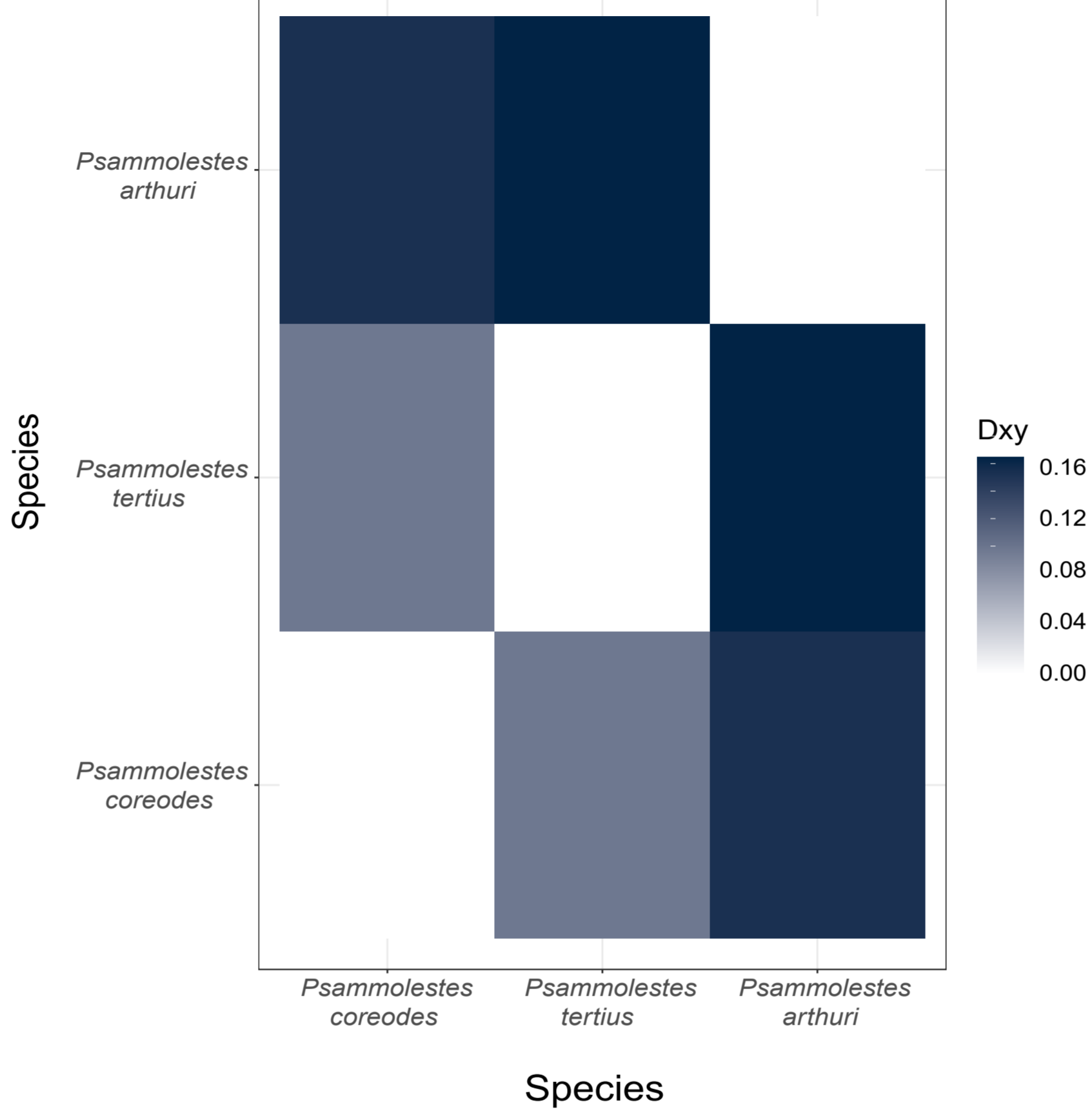

C

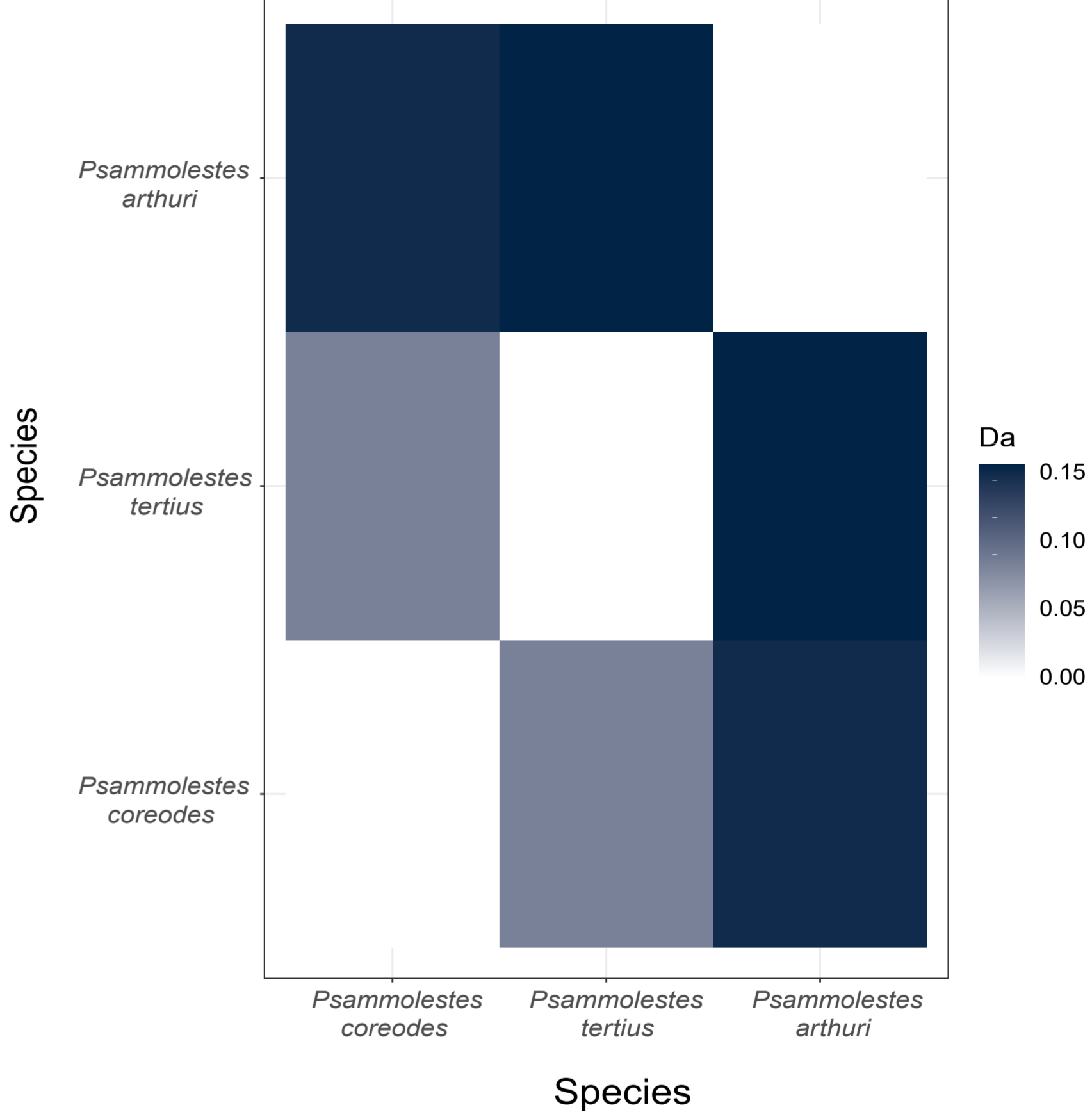

Supplement: Supplementary file 13 — Additional file 13. Heatmaps calculated for three different statistics: A) Fst, B) Dxy and C) Da for three species based on the molecular data obtained from the mitochondrial marker CYTB. [file 12862_2022_1987_MOESM13_ESM.pdf]

A

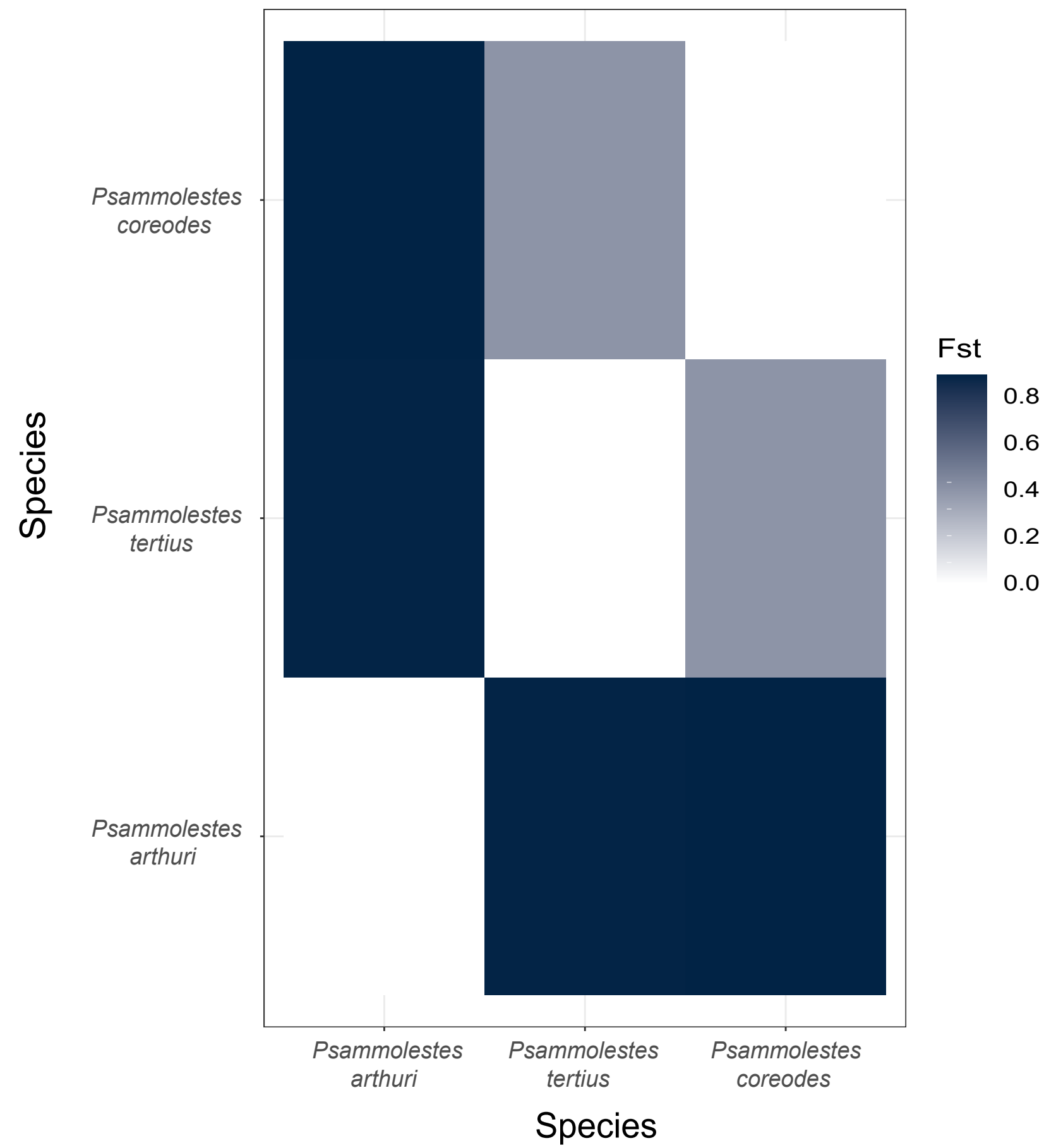

B

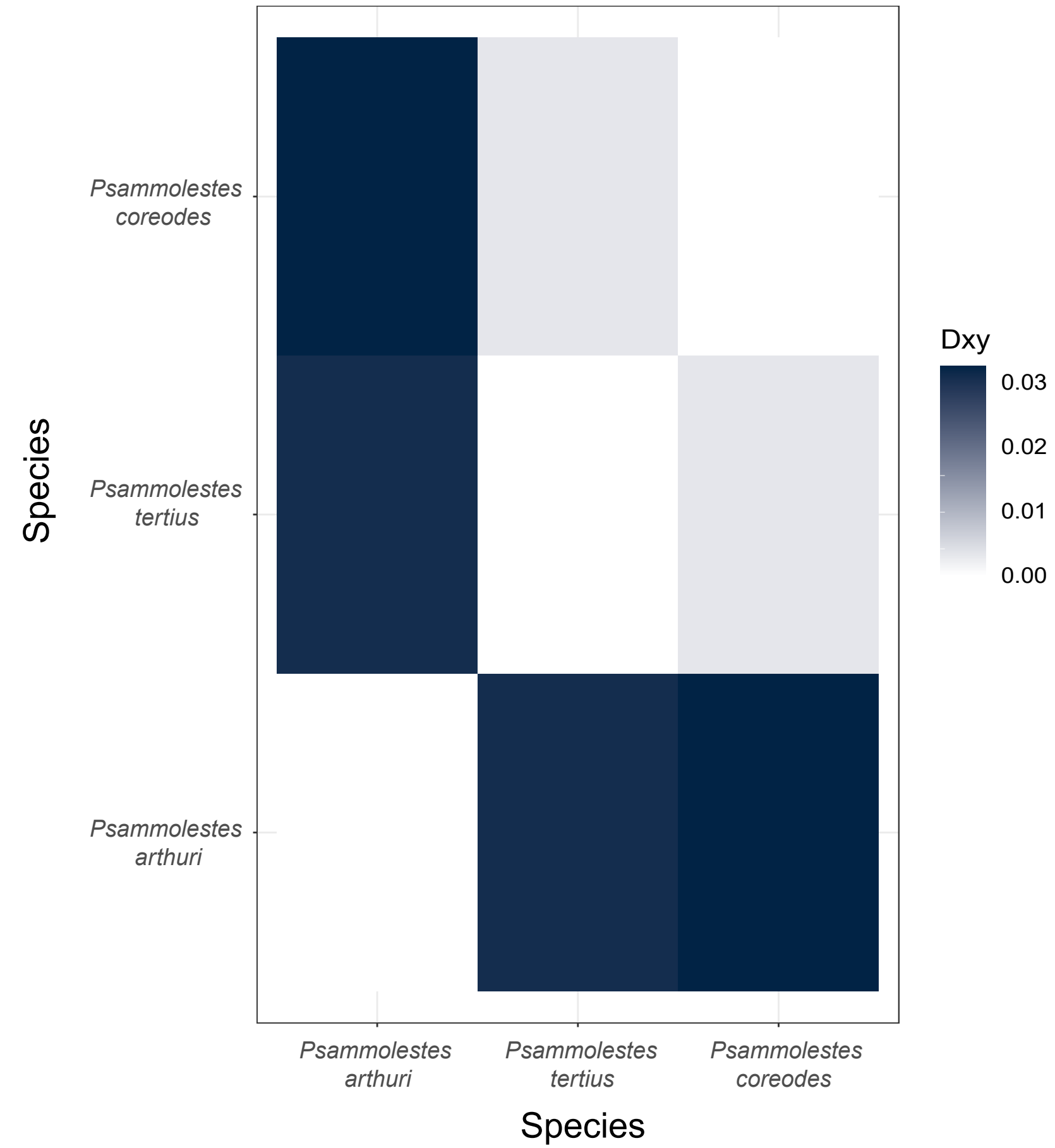

C

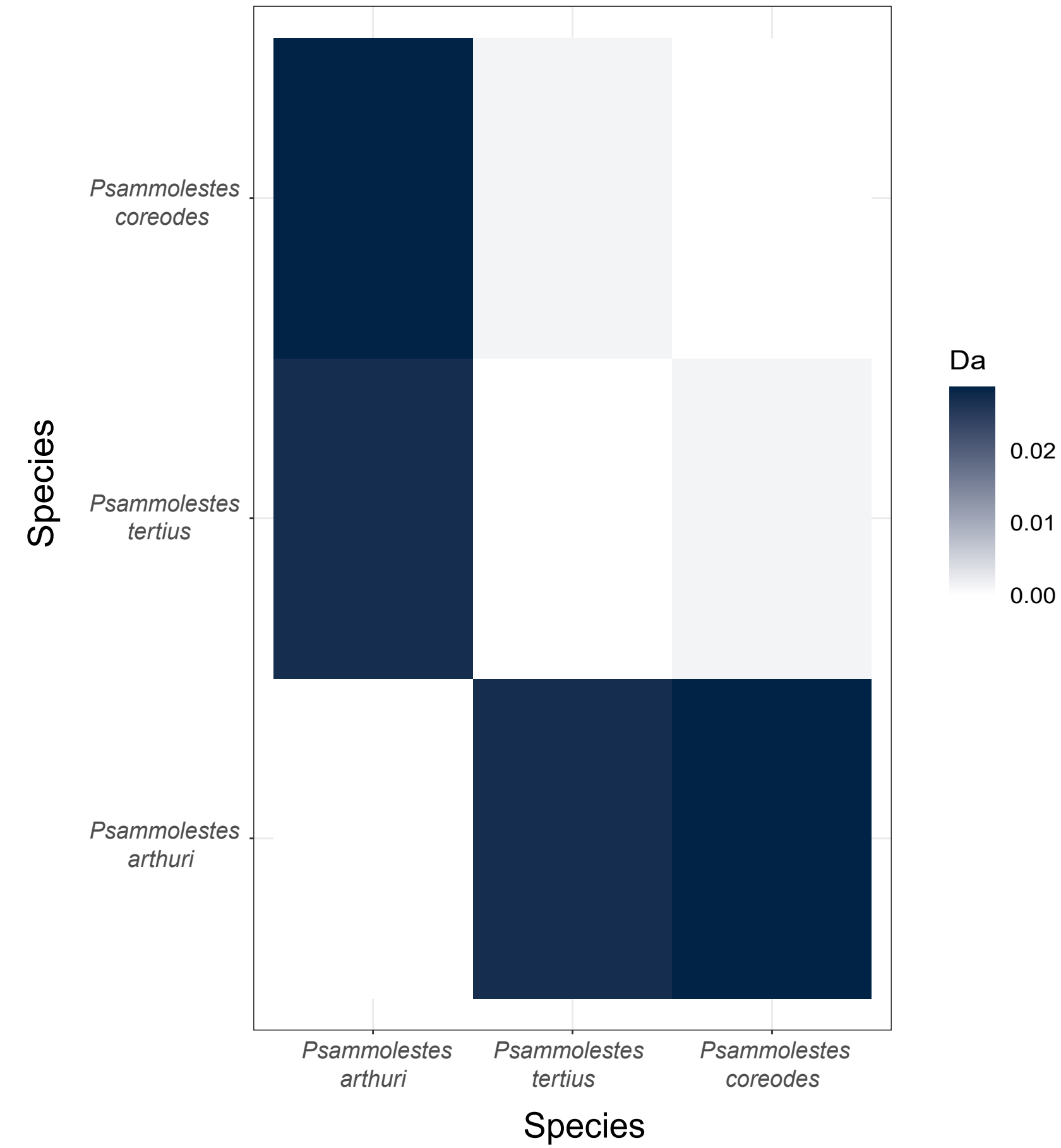

Supplement: Supplementary file 14 — Additional file 14. Heatmaps calculated for three different statistics: A) Fst, B) Dxy and C) Da for three species based on the molecular data obtained from the nuclear marker LSM. [file 12862_2022_1987_MOESM14_ESM.pdf]

A

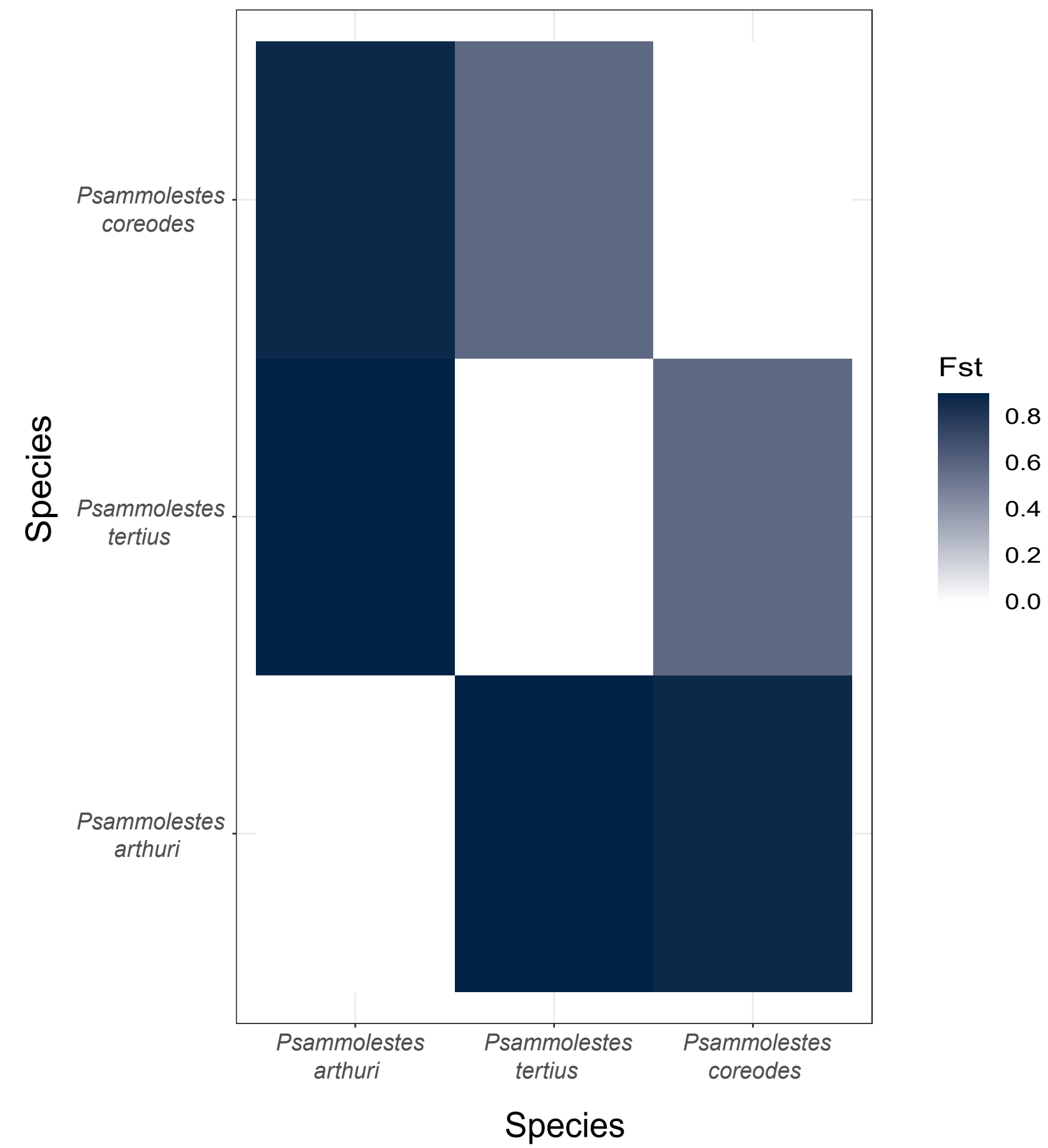

B

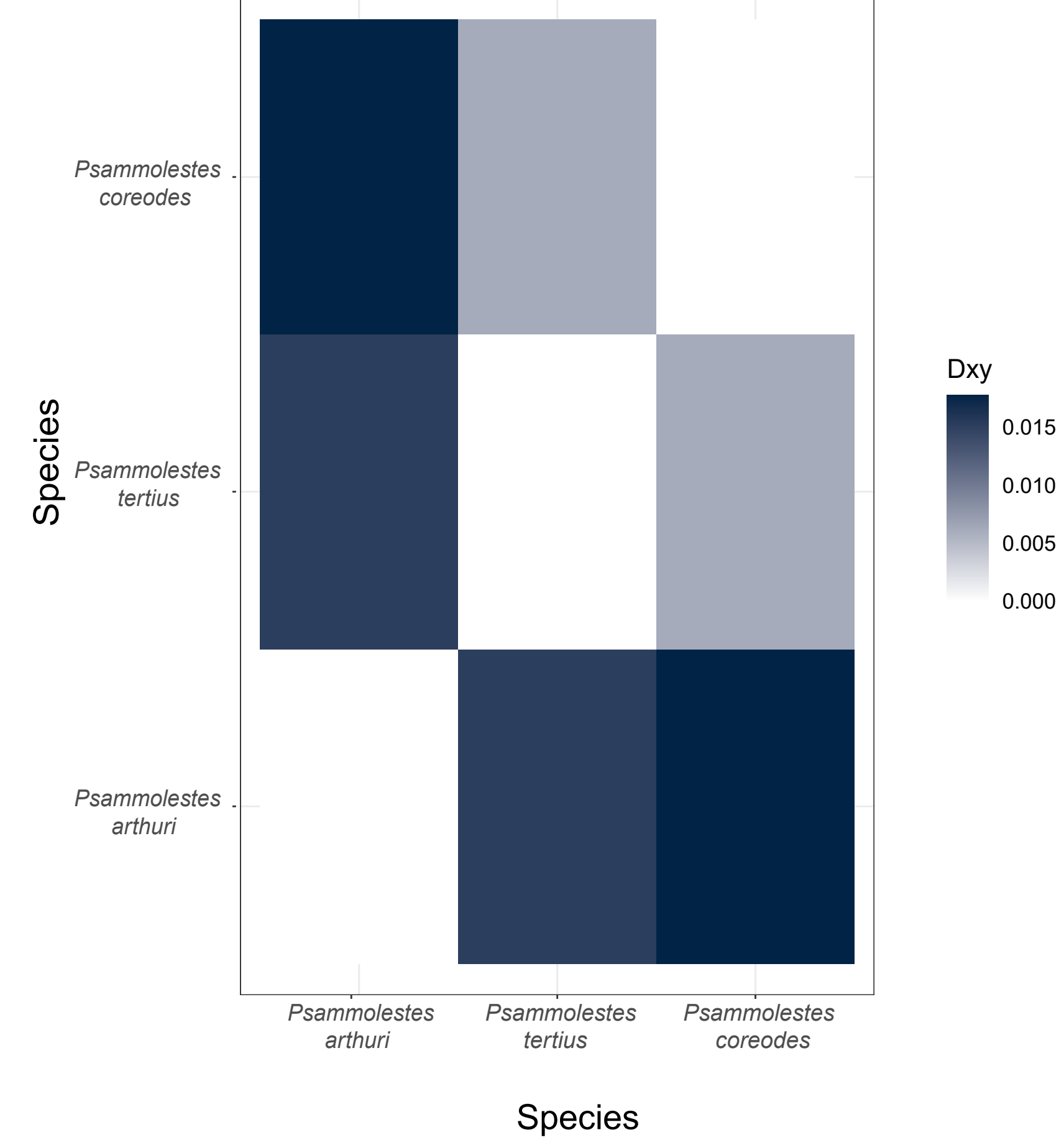

C

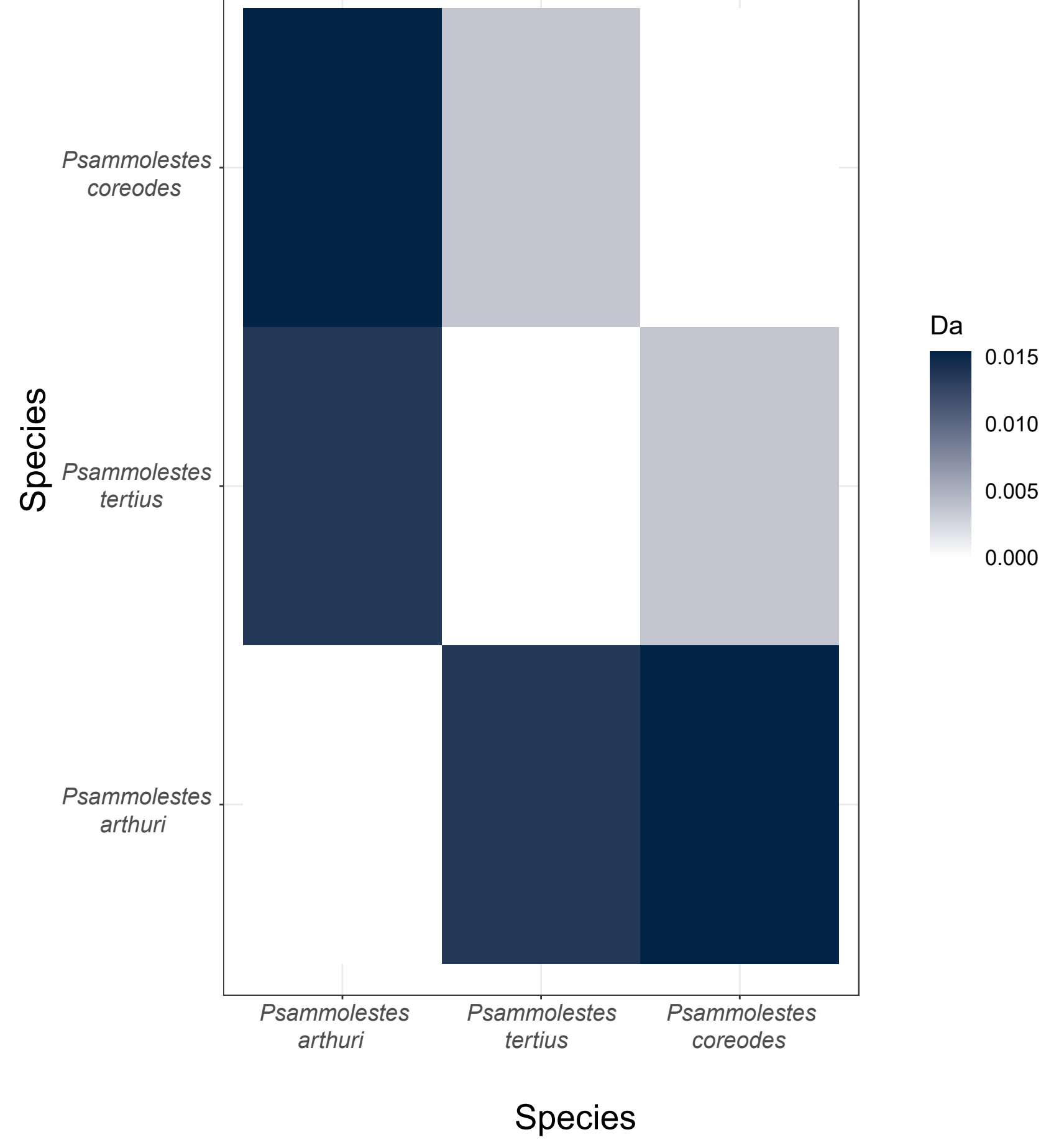

Supplement: Supplementary file 15 — Additional file 15. Heatmaps calculated for three different statistics: A) Fst, B) Dxy and C) Da for three species based on the molecular data obtained from the nuclear marker PJH. [file 12862_2022_1987_MOESM15_ESM.pdf]

A

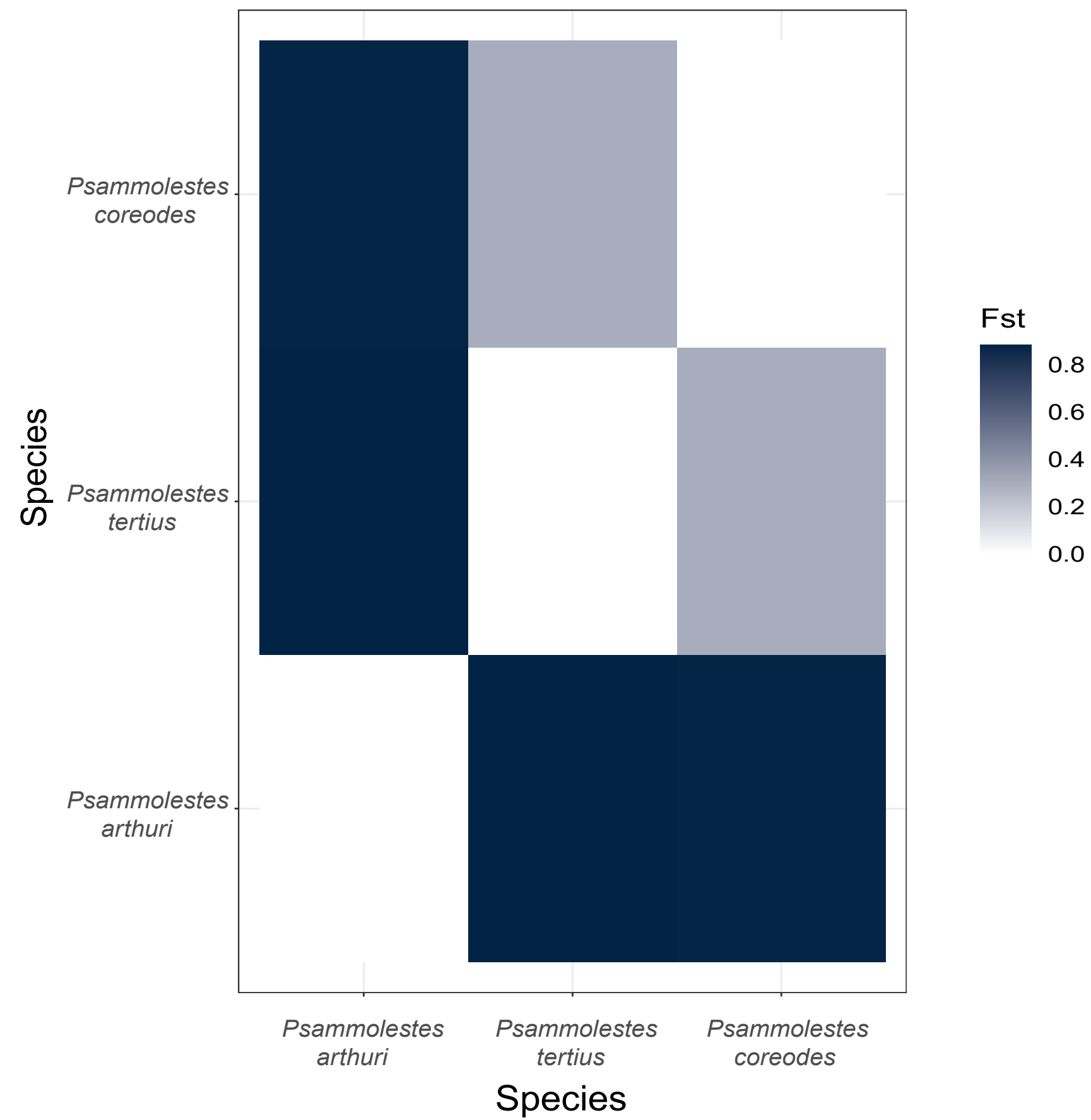

B

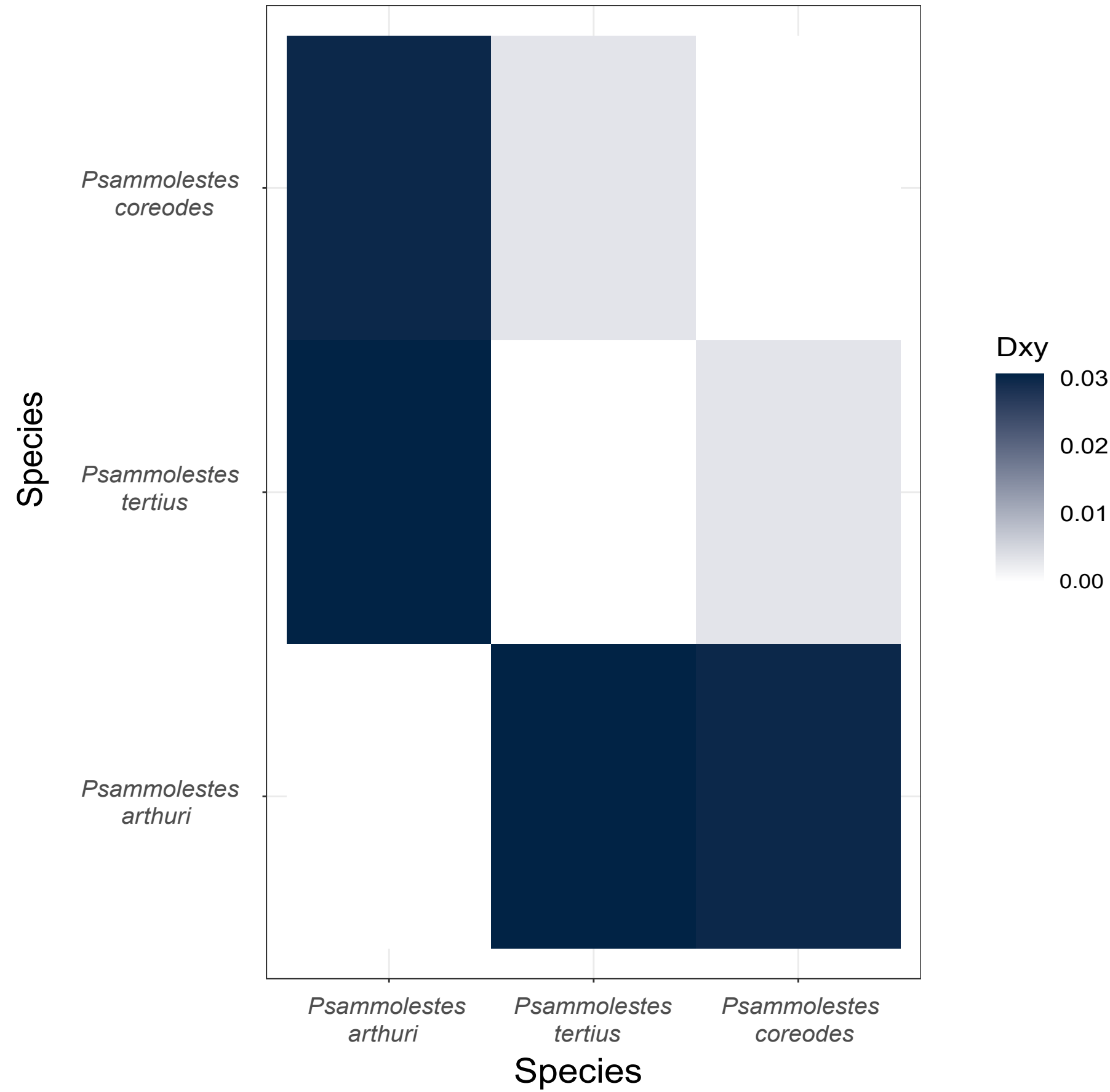

C

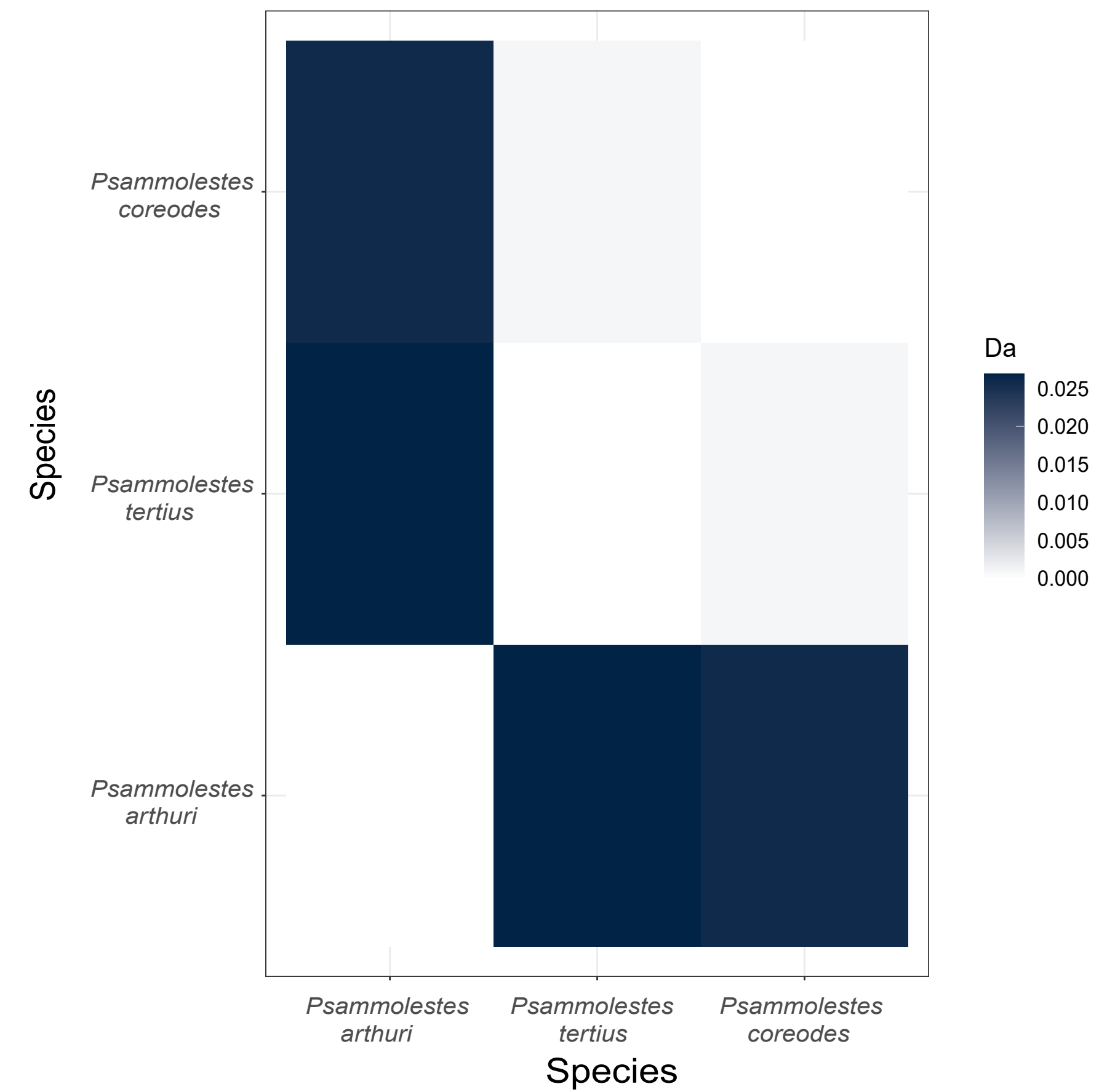

Supplement: Supplementary file 16 — Additional file 16. Heatmaps calculated for three different statistics: A) Fst, B) Dxy and C) Da for three species based on the molecular data obtained from the nuclear marker TRNA. [file 12862_2022_1987_MOESM16_ESM.pdf]

A

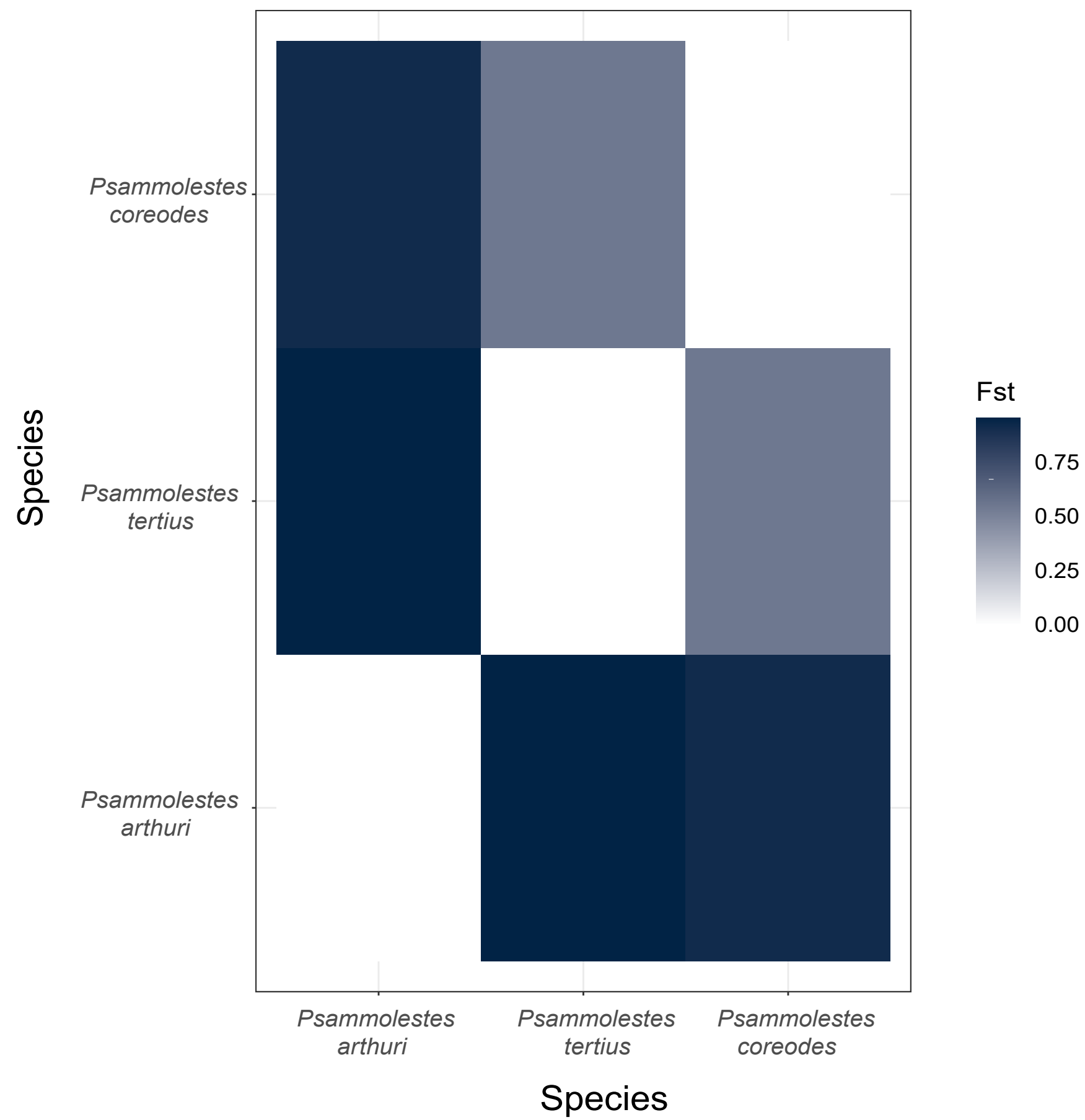

B

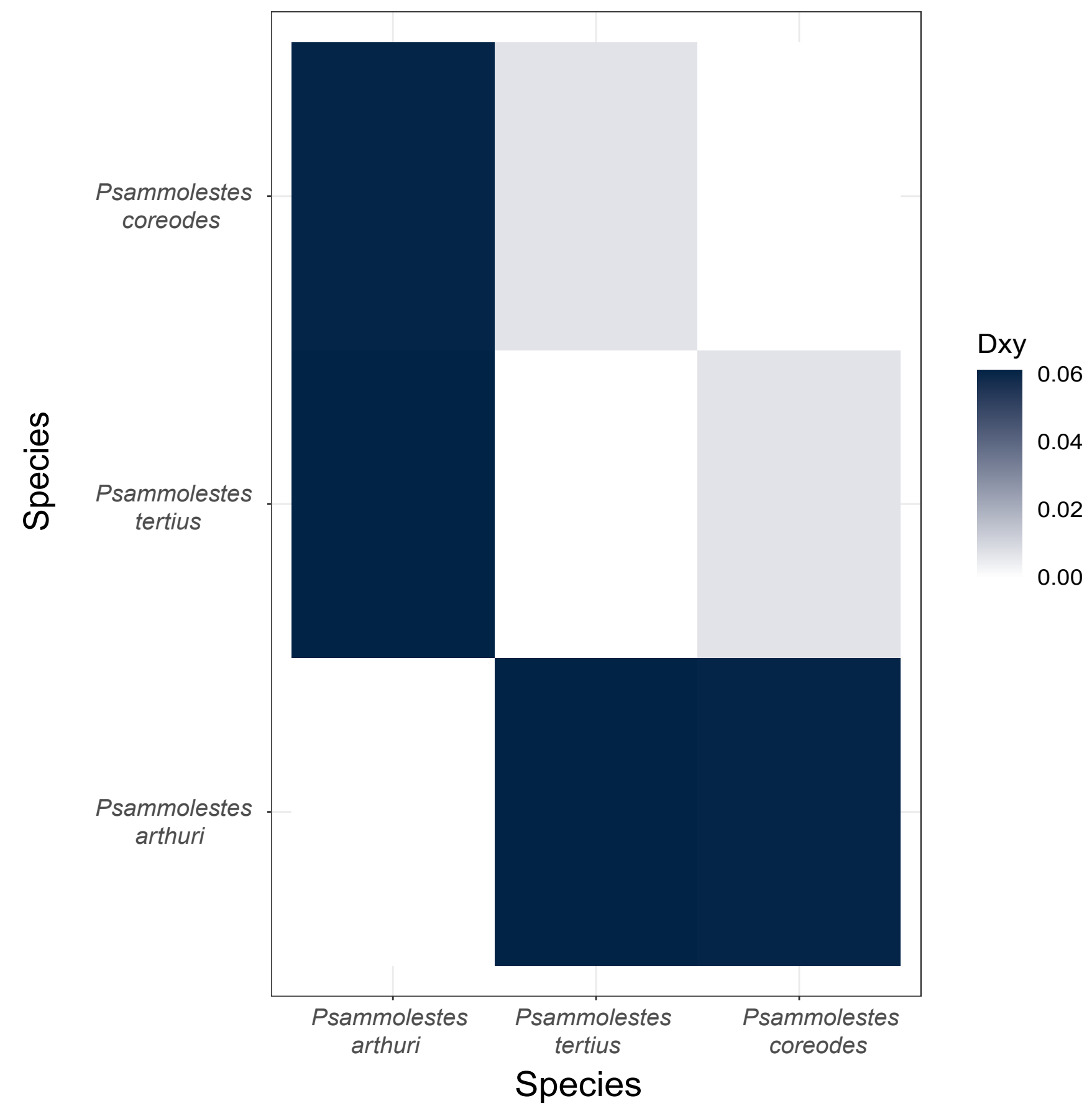

C

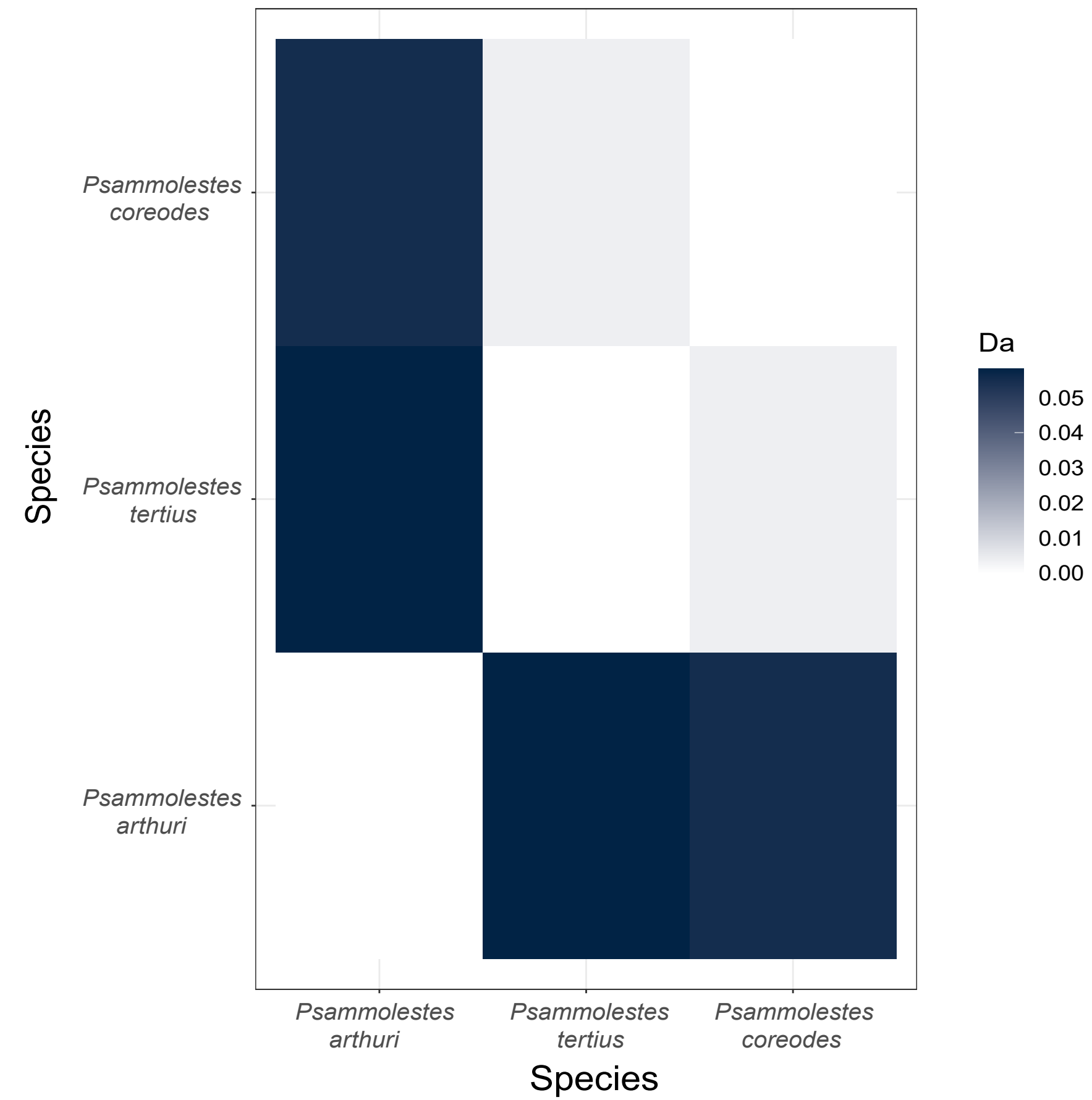

Supplement: Supplementary file 17 — Additional file 17. Heatmaps calculated for three different statistics: A) Fst, B) Dxy and C) Da for three species based on the molecular data obtained from the nuclear marker UPCA. [file 12862_2022_1987_MOESM17_ESM.pdf]

A

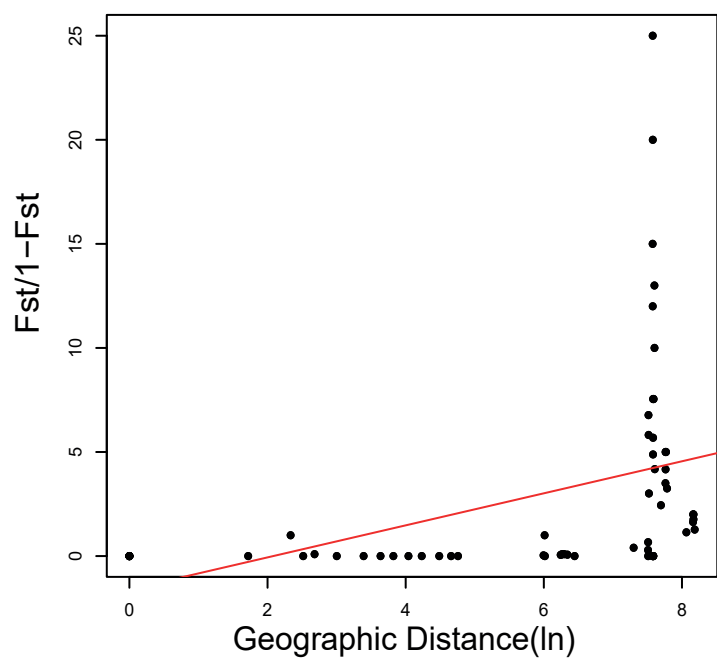

B

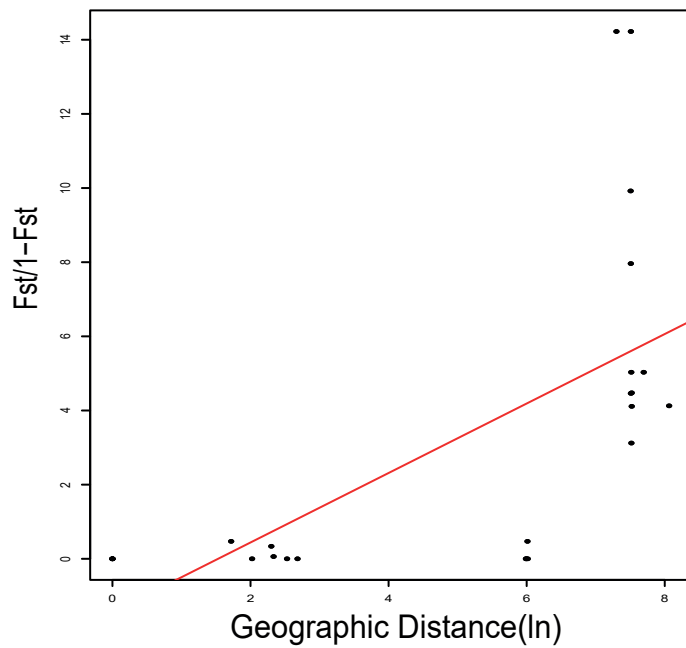

C

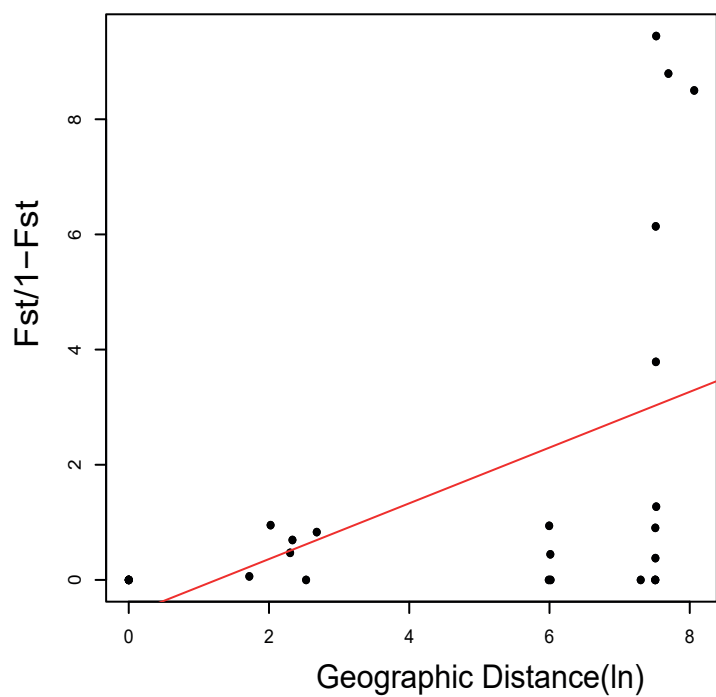

D

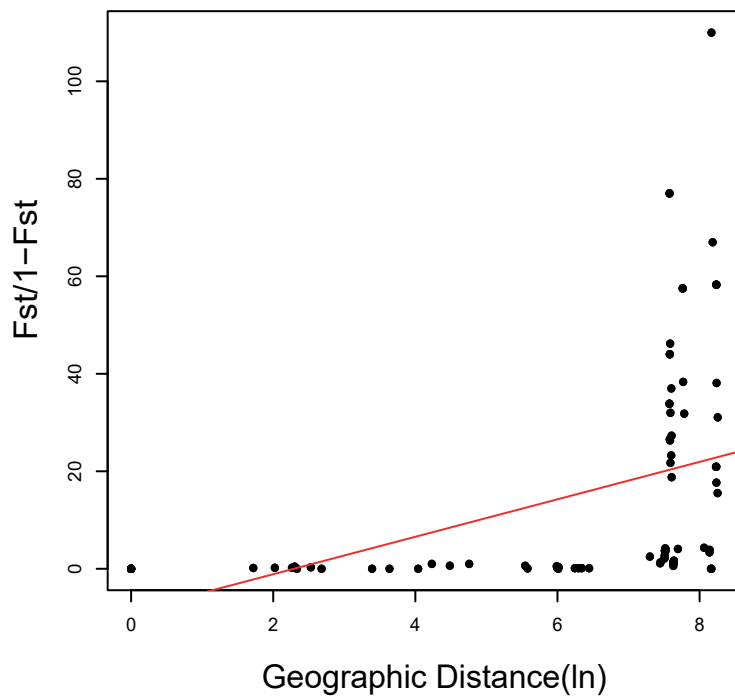

E

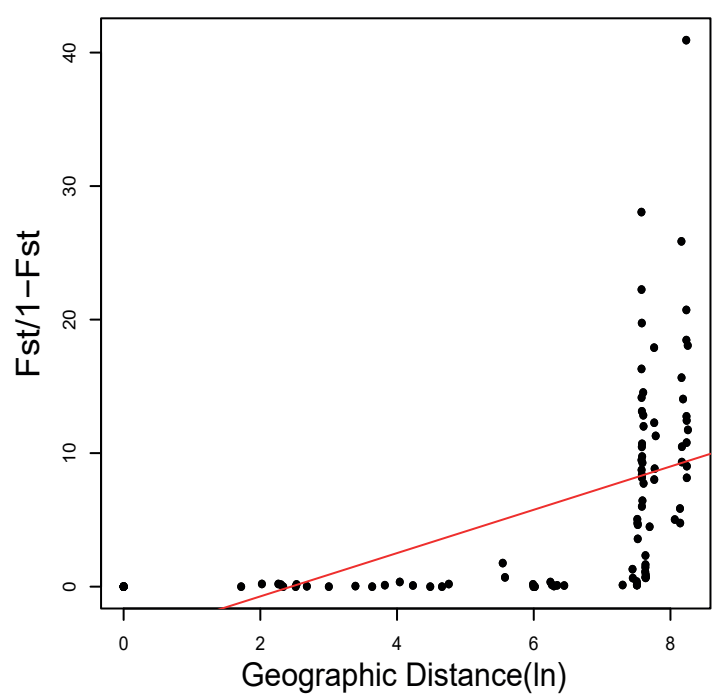

F

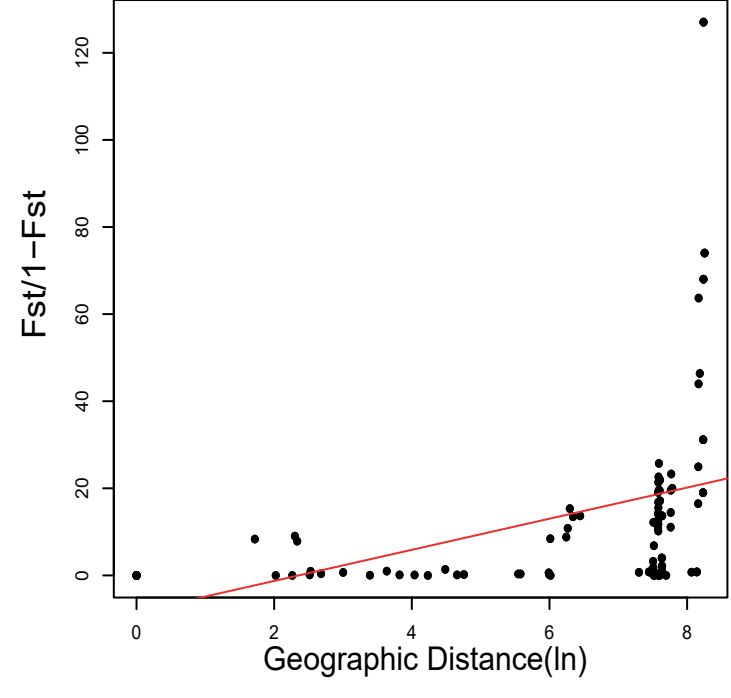

G

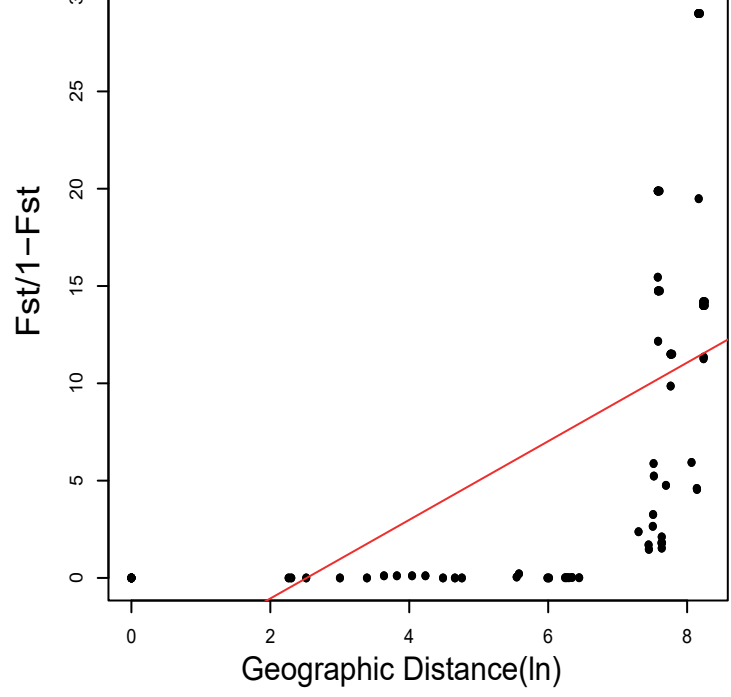

Supplement: Supplementary file 19 — Additional file 19. Linear correlations of Isolation by distance (IBD) test. (A) 28S (B) CISP (C) CYTB (D) LSM (E) TRNA (F) UPCA (G) PJH. [file 12862_2022_1987_MOESM19_ESM.pdf]
