# Supplementary material for: Phylogenetic relationships and evolutionary patterns of the genus Psammolestes Bergroth, 1911 (Hemiptera: Reduviidae: Triatominae)
Source: BMC Ecol Evol. 2022 Mar 12;22:30. doi: 10.1186/s12862-022-01987-x (PMC8918316; doi:10.1186/s12862-022-01987-x)
Supplement: Supplementary file 18 — Additional file 18. Demographic models created with Phylogeographic Inference Using Approximate Likelihoods (PHRAPL) to test the evolution of Psammolestes. (A) Divergence with no migration (B) Divergence with bidirectional migration between P. coreodes and P. tertius. (C) Divergence with bidirectional migration between P. tertius and P. arthuri. (D) divergence with bidirectional migration between P. tertius with P. coreodes, and P. tertius with P. arthuri. (E) Divergence with bidirectional migration between P. coreodes and P. arthuri. (F) Divergence with bidirectional migration between P. tertius with P. coreodes, and P. coreodes with P. arthuri. (G) Divergence with bidirectional migration between P. tertius with P. arthuri, and P. arthuri with P. coreodes (H) Divergence with bidirectional migration between the three Psammolestes species. Starting from this point, all of the demographic models include bidirectional migration between P. arthuri and the MRCA (most recent common ancestor) of P. tertius and P. coreodes. (I) Divergence with bidirectional migration between P. arthuri and the MRCA of P. tertius and P. coreodes. (J) Divergence with bidirectional migration between P. coreodes and P. tertius. (K) Divergence with bidirectional migration between P. tertius and P. arthuri. (L) divergence with bidirectional migration between P. tertius with P. coreodes and P. arthuri. (M) Divergence with bidirectional migration between P. coreodes and P. arthuri. (N) divergence with bidirectional migration between P. coreodes with P. tertius and P. arthuri. (O) Divergence with bidirectional migration between P. arthuri with P. coreodes and P. tertius. (P) Divergence with bidirectional migration between the three Psammolestes species. Support values for the demographic scenarios are shown under each figure. [file 12862_2022_1987_MOESM18_ESM.pdf]

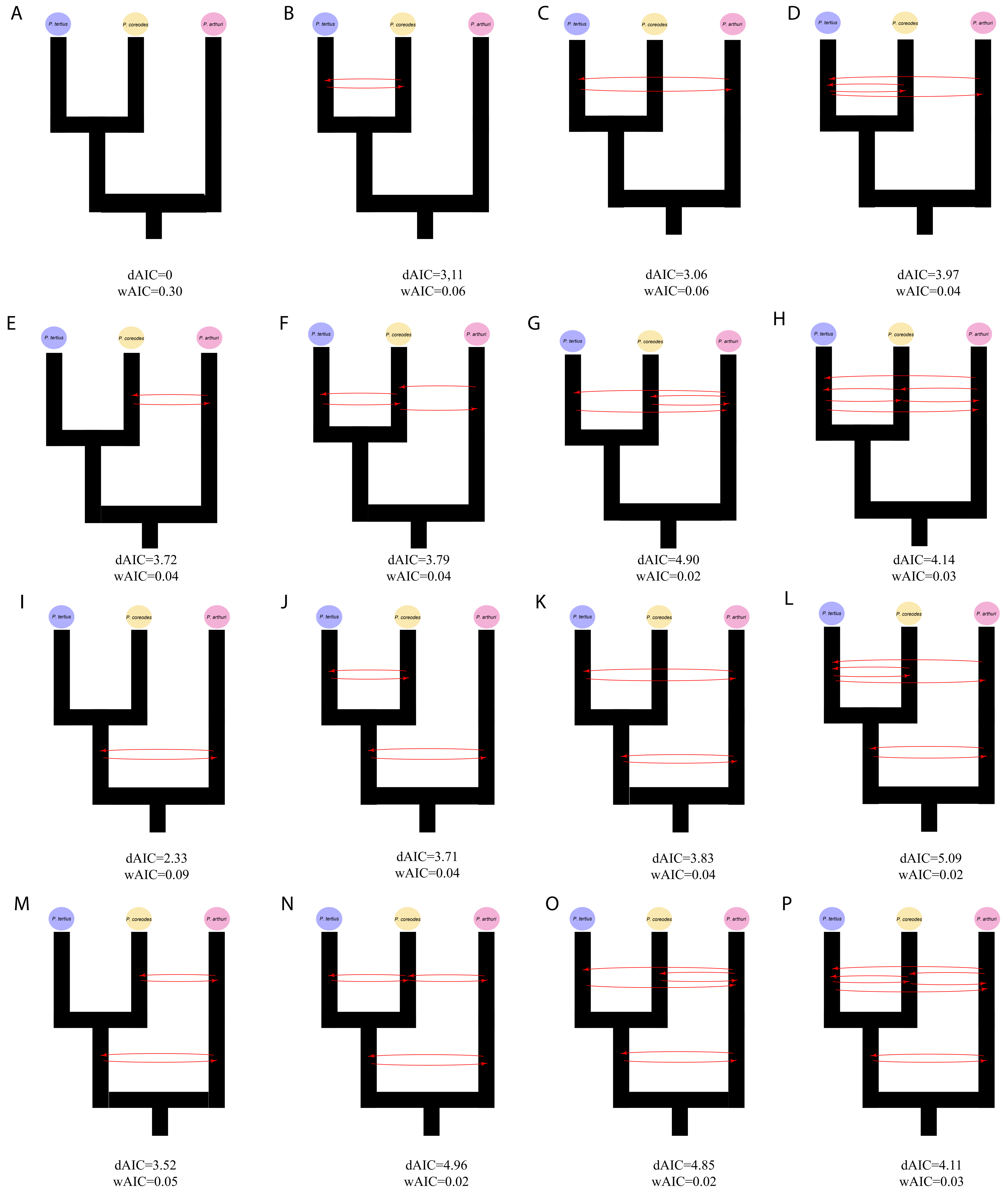
A
P. tertius
P. coreodes
P. arthuri
dAIC=0
wAIC=0.30
B
dAIC=3,11
wAIC=0.06
C
dAIC=3.06
wAIC=0.06
D
dAIC=3.97
wAIC=0.04
E
dAIC=3.72
wAIC=0.04
F
dAIC=3.79
wAIC=0.04
G
dAIC=4.90
wAIC=0.02
H
dAIC=4.14
wAIC=0.03
I
dAIC=2.33
wAIC=0.09
J
dAIC=3.71
wAIC=0.04
K
dAIC=3.83
wAIC=0.04
L
dAIC=5.09
wAIC=0.02
M
dAIC=3.52
wAIC=0.05
N
dAIC=4.96
wAIC=0.02
O
dAIC=4.85
wAIC=0.02
P
dAIC=4.11
wAIC=0.03
