# Supplementary material for: Phylogenetic relationships and evolutionary patterns of the genus Psammolestes Bergroth, 1911 (Hemiptera: Reduviidae: Triatominae)
Source: BMC Ecol Evol. 2022 Mar 12;22:30. doi: 10.1186/s12862-022-01987-x (PMC8918316; doi:10.1186/s12862-022-01987-x)
Supplement: Supplementary file 20 — Additional file 20. Mantel’s test for isolation by distance (IBD) and linear correlation results. The result of the Mantel’s test is shown in the two first columns of the table, and the results of the Pearson’s correlation test correspond to the third column. The last two columns show the results of the linear correlation tested between geographical and genetic distances. [file 12862_2022_1987_MOESM20_ESM.pdf]

Additional file 20. Mantel's test for isolation by distance (IBD) and linear correlation results. The result of the Mantel's test is shown in the two first columns of the table, and the results of the Pearson's correlation test correspond to the third column. The last two columns show the results of the linear correlation tested between geographical and genetic distances.

Locus	Mantel's R	Mantel's p-value	Pearson's R	R^2	Corr. p-value
**28S**	0.3791	0.003	0.3884	0.1437	1.07e-05
**CISP**	0.5112	0.002	0.5158	0.2623	1.01e-14
**CYTB**	0.6205	0.041	0.6372	0.3934	8.51e-07
**LSM**	0.4150	0.001	0.4205	0.1719	1.24e-08
**PJH**	0.6218	0.001	0.5947	0.3492	3.83e-15
**TRNA**	0.5281	0.001	0.5234	0.2702	3.50e-15
**UPCA**	0.3727	0.001	0.3776	0.1382	4.83e-08
