# Supplementary material for: Phylogenetic relationships and evolutionary patterns of the genus Psammolestes Bergroth, 1911 (Hemiptera: Reduviidae: Triatominae)
Source: BMC Ecol Evol. 2022 Mar 12;22:30. doi: 10.1186/s12862-022-01987-x (PMC8918316; doi:10.1186/s12862-022-01987-x)
Supplement: Supplementary file 21 — Additional file 21. Fit of the demographic models tested in. [file 12862_2022_1987_MOESM21_ESM.pdf]

Additional file 21 Fit of the demographic models tested in PHRAPL.

MODEL	AIC	DAIC	WAIC	LNL
**A**	185.4643	0	0.3081831	-90.73213
**B**	188.5775	3.113272	0.06497862	-91.28876
**C**	188.5265	3.062229	0.0666583	-91.26324
**D**	189.444	3.979795	0.04213153	-91.72202
**E**	189.1866	3.722395	0.04791825	-91.59332
**F**	189.2615	3.797275	0.04615736	-91.63076
**G**	190.3696	4.905344	0.02652326	-92.1848
**H**	189.6047	4.140475	0.03887908	-91.80236
**I**	187.8014	2.337145	0.09578649	-90.9007
**J**	189.8	3.715758	0.04807753	-91.59
**K**	189.2965	3.832203	0.04535827	-91.64823
**L**	190.5584	5.094104	0.02413449	-92.27918
**M**	188.9866	3.522318	0.0529599	-91.49328
**N**	190.4304	4.966184	0.02572857	-92.21522
**O**	190.3223	4.858053	0.02715789	-92.16115
**P**	189.5798	4.115515	0.03936734	-91.78988

A Divergence with no migration. **B**. Divergence with bidirectional migration between *P. coreodes* and *P. tertius*. **C.** Divergence with bidirectional migration between *P. tertius* and *P. arthuri.* **D.** Divergence with bidirectional migration between *P. tertius* with *P. coreodes*, and *P. tertius* with *P. arthuri*. **E.** Divergence with bidirectional migration between *P. coreodes* and *P. arthuri*. **F.** Divergence with bidirectional migration between *P. tertius* with *P. coreodes*, and *P. coreodes* with *P. arthuri*. **G.** Divergence with bidirectional migration between *P. tertius* with *P. arthuri*, and *P. arthuri* with *P. coreodes*. **H.** Divergence with bidirectional migration between the three *Psammolestes species*. Starting from this point, all the demographic models include bidirectional migration between *P. arthuri* and the MRCA (most recent common ancestor) of *P. tertius* and *P. coreodes*. **I.** Divergence with bidirectional migration between *P. arthuri* and the MRCA of *P. tertius* and *P. coreodes*. **J.** Divergence with bidirectional migration between *P. coreodes* and *P. tertius*. **K** Divergence with bidirectional migration between *P. tertius* and *P. arthuri*. **L**. Divergence with bidirectional migration between *P. tertius* with *P. coreodes* and *P. arthuri*. **M**. Divergence with bidirectional migration between *P. coreodes* and *P. arthuri*. **N.** Divergence with bidirectional migration between *P. coreodes* with *P. tertius* and *P. arthuri*. **O**. Divergence with bidirectional migration between *P. arthuri* with *P. coreodes* and *P. tertius*. **P.** Divergence with bidirectional migration between the three *Psammolestes* species.
