# Supplementary material for: Phylogenetic relationships and evolutionary patterns of the genus Psammolestes Bergroth, 1911 (Hemiptera: Reduviidae: Triatominae)
Source: BMC Ecol Evol. 2022 Mar 12;22:30. doi: 10.1186/s12862-022-01987-x (PMC8918316; doi:10.1186/s12862-022-01987-x)
Supplement: Supplementary file 22 — Additional file 22. Individuals of Psammolestes species collected in this study. [file 12862_2022_1987_MOESM22_ESM.pdf]

Additional file 22. Individuals of *Psammolestes* species collected in this study

Species	Country	n
P. arthuri	Colombia	24
	Venezuela	15
P. coreodes	Brasil	28
P. tertius	Brasil	25
