# Supplementary material for: Phylogenetic relationships and evolutionary patterns of the genus Psammolestes Bergroth, 1911 (Hemiptera: Reduviidae: Triatominae)
Source: BMC Ecol Evol. 2022 Mar 12;22:30. doi: 10.1186/s12862-022-01987-x (PMC8918316; doi:10.1186/s12862-022-01987-x)
Supplement: Supplementary file 23 — Additional file 23. Genes included in this study, the primers used to obtain their corresponding sequence, and the length of each one of them. “*” symbolizes a new marker used for the delimitation of the Psammolestes species. [file 12862_2022_1987_MOESM23_ESM.pdf]

Additional file 23. Genes included in this study, the primers used to obtain their corresponding sequence, and the length of each one of them. "*" symbolizes a new marker used for the delimitation of the *Psammolestes* species.

Gen name	Primer Sequence	Size (bp)
tRNA (Guanine (37) -N (1) methyltransferase *	Forward: GGGCCACGTTTCTAACAAAA Reverse: CAATTGGAATGCTGCTGAAA	842
Putative juvenile hormone inducible protein *	Forward: CCCTTTTAGCAAAATGTTCCA Reverse: TGCCATTATTGCAAGCAGAA	720
Probable cytosolic iron sulfur protein assembly protein Ciao 1*	Forward: TTATCTGCGCAAGCAGTAGC Reverse: TAAGACTTTGGGGGAAGCAA	706
Lipoyl synthase, mitocondrial *	Forward: AAAAAGCCCATTTCGTTTCC Reverse: AATGGGCCCACATTATTCAA	768
Uncharacterized protein (Cell adhesion) *	Forward: TGAAAGGGATCGTACCTTGG Reverse: CCTCCAGACTGATGGCTTGT	795
Cytochrome b	Forward: GGACG(AT)GG(AT)ATTTATTATGGATC Reverse: GC(AT)CCAATTCA(AG)GTTA(AG)TAA	840
**28S**	Iniciador Forward: GCGAGTCGTGTTGCTTGATAGTGCAG Iniciador Reverse: TTGGTCCGTGTTTCAAGACGGG	696
