# Supplementary material for: Phylogenetic relationships and evolutionary patterns of the genus Psammolestes Bergroth, 1911 (Hemiptera: Reduviidae: Triatominae)
Source: BMC Ecol Evol. 2022 Mar 12;22:30. doi: 10.1186/s12862-022-01987-x (PMC8918316; doi:10.1186/s12862-022-01987-x)
Supplement: Supplementary file 24 — Additional file 24. GenBank accession numbers of seven loci analyzed in this study and samples origin included in this study. [file 12862_2022_1987_MOESM24_ESM.pdf]

**Table 24. GenBank accession numbers of seven loci analyzed in this study and samples origin included in this study**

| Sequence_ID | *Species* | Country | City | Localities | 28s | CYTB | LSM | PJH | UPCA | TRNA | CISP |
|---|---|---|---|---|---|---|---|---|---|---|---|
| 224_Part_Col_Cas_Man | *P. arthuri* | Colombia | Casanare | Mani | OL584419 | * | * | OM256834 | OM256993 | * | * |
| 225_Part_Col_Cas_Man | *P. arthuri* | Colombia | Casanare | Mani | OL584420 | * | * | OM256835 | OM256994 | OM256912 | OM291459 |
| 226_Part_Col_Cas_Man | *P. arthuri* | Colombia | Casanare | Mani | OL584421 | * | * | OM256836 | * | OM256913 | OM291460 |
| 227_Part_Col_Cas_Man | *P. arthuri* | Colombia | Casanare | Mani | OL584422 | * | OM291374 | OM256837 | OM256995 | OM256914 | OM291461 |
| 228_Part_Col_Cas_Man | *P. arthuri* | Colombia | Casanare | Mani | OL584423 | * | * | OM256838 | OM256996 | * | * |
| 229_Part_Col_Cas_Man | *P. arthuri* | Colombia | Casanare | Mani | OL584424 | * | * | OM256839 | OM256997 | * | OM291462 |
| 230_Part_Col_Cas_Man | *P. arthuri* | Colombia | Casanare | Mani | OL584425 | * | * | OM256840 | OM256998 | OM256915 | OM291463 |
| 231_Part_Col_Cas_Man | *P. arthuri* | Colombia | Casanare | Mani | OL584426 | * | * | OM256841 | OM256999 | * | * |
| 232_Part | *P. arthuri* | Colombia | Casanare | Mani |  | * | OM291375 | OM256842 | OM257000 | OM256916 | OM291464 |
| 233_Part_Col_Cas_Man | *P. arthuri* | Colombia | Casanare | Mani | OL584427 | * | * | OM256843 | OM257001 | OM256917 | OM291465 |
| 234_Part_Col_Cas_Man | *P. arthuri* | Colombia | Casanare | Mani | OL584428 | * | * | OM256844 | OM257002 | OM256918 | OM291466 |
| 499_Pter | *P. tertius* | Brazil | Bahia | Castro Alves | * | * | * | OM256845 | OM257003 | OM256919 | OM291467 |
| 500_Pter_Bra_Bah_Cas | *P. tertius* | Brazil | Bahia | Castro Alves | * | * | OM291376 | * | * | * | * |
| 502_Pter | *P. tertius* | Brazil | Bahia | Castro Alves | * | * | OM291377 | OM256846 | OM257004 | OM256920 | OM291468 |
| 504_Pter | *P. tertius* | Brazil | Bahia | Castro Alves | * | * | OM291378 | OM256847 | OM257005 | * | OM291469 |
| 505_Pter | *P. tertius* | Brazil | Bahia | Castro Alves | * | * | * | * | OM257006 | * | * |
| 506_Pter | *P. tertius* | Brazil | Bahia | Castro Alves | * | * | OM291379 | OM256848 | * | * | OM291470 |
| 507_Pter | *P. tertius* | Brazil | Bahia | Castro Alves | * | * | * | OM256849 | OM257007 | OM256921 | * |
| 508_Pter | *P. tertius* | Brazil | Bahia | Castro Alves |  |  | * | * | * | * | OM291471 |
| 510_Pcor_Bra_Mat_Cor | *P.coreodes* | Brazil | Mato Grosso do Sul | Corumba | * | OM291422 | * | OM256850 | OM257008 | OM256922 | OM291472 |
| 511_Pcor | *P.coreodes* | Brazil | Mato Grosso do Sul | Corumba | * | OM291423 | * | OM256851 | OM257009 | OM256923 | OM291473 |
| 512_Pcor_Bra_Mat_Cor | *P.coreodes* | Brazil | Mato Grosso do Sul | Corumba | * | OM291424 | OM291380 | OM256852 | OM257010 | OM256924 | * |
| 513_Pcor | *P.coreodes* | Brazil | Mato Grosso do Sul | Corumba | * | * | * | OM256853 | * | * | OM291474 |
| 514_Part_Ven_Ara_Mar | *P. arthuri* | Venezuela | Aragua | Marbacella | OL584429 | OM291425 | * | OM256854 | OM257011 | OM256925 | OM291475 |
| 515_Part_Ven_Ara_Mar | *P. arthuri* | Venezuela | Aragua | Marbacella | OL584430 | OM291426 | OM291381 | OM256855 | OM257012 | OM256926 | OM291476 |
| 516_Part | *P. arthuri* | Venezuela | Aragua | Marbacella | * | OM291427 | * | OM256856 | OM257013 | OM256927 | OM291477 |
| 517_Part | *P. arthuri* | Venezuela | Aragua | Marbacella | * | OM291428 | * | * | OM257014 | * | OM291478 |
| 613_Pter | *P. tertius* | Brazil | Bahia | Castro Alves | * | * | OM291382 | OM256857 | * | OM256928 | OM291479 |
| 614_Pter | *P. tertius* | Brazil | Bahia | Castro Alves | * | OM291429 | OM291383 | OM256858 | OM257015 | OM256929 | OM291480 |
| 615_Pter | *P. tertius* | Brazil | Bahia | Castro Alves | * | * | OM291384 | OM256859 | * | OM256930 | OM291481 |
| 616_Pter | *P. tertius* | Brazil | Bahia | Castro Alves | * | * | OM291385 | OM256860 | OM257016 | OM256931 | OM291482 |
| 617_Pter | *P. tertius* | Brazil | Bahia | Castro Alves | * | * | * | OM256861 | OM257017 | OM256932 | * |
| 623_Pter | *P. tertius* | Brazil | Bahia | Santa Teresinha | * | OM291430 | OM291386 | OM256862 | OM257018 | OM256933 | OM291483 |
| 625_Pter | *P. tertius* | Brazil | Bahia | Santa Teresinha | * | * | OM291387 | OM256863 | OM257019 | OM256934 | * |
| 630_Pter | *P. tertius* | Brazil | Bahia | Santa Teresinha | * | * | OM291388 | OM256864 | * | OM256935 | OM291484 |
| 631_Pter_Bra_Bah_San | *P. tertius* | Brazil | Bahia | Santa Teresinha | * | * | * | OM256865 | * | OM256936 | OM291485 |
| 632_Pter | *P. tertius* | Brazil | Bahia | Santa Teresinha | * | * | OM291389 | OM256866 | * | OM256937 | OM291486 |
| 633_Pter_Bra_Bah_Sea | *P. tertius* | Brazil | Bahia | Seabra | * | OM291431 | OM291390 | OM256867 | * | OM256938 | OM291487 |
| 634_Pter_Bra_Bah_Sea | *P. tertius* | Brazil | Bahia | Seabra | * | OM291432 | * | OM256868 | * | OM256939 | OM291488 |
| 635_Pter | *P. tertius* | Brazil | Bahia | Seabra | * | * | OM291391 | OM256869 | OM257020 | OM256940 | OM291489 |
| 636_Pter | *P. tertius* | Brazil | Bahia | Seabra | * | OM291433 | OM291392 | OM256870 | OM257021 | OM256942 | OM291490 |
| 637_Pter | *P. tertius* | Brazil | Bahia | Seabra | * | * | * | OM256871 | OM257022 | OM256943 | * |
| 647_Pter | *P. tertius* | Brazil | Mato Grosso do Sul | Corumba | * | * | * | OM256872 | OM257023 | OM256944 | OM291491 |
| 648_Pcor_Bra_Mat_Cor | *P. coreodes* | Brazil | Mato Grosso do Sul | Corumba | * | OM291434 | OM291393 | OM256873 | OM257024 | OM256945 | OM291492 |
| 649_Pcor | *P. coreodes* | Brazil | Mato Grosso do Sul | Corumba | * | OM291435 | * | OM256874 | OM257025 | OM256946 | OM291493 |

| Table 24. GenBank accession numbers of seven loci analyzed in this study and samples origin included in this study | | | | | | | | | | | |
|---|---|---|---|---|---|---|---|---|---|---|---|
| **Sequence_ID** | ***Species*** | **Country** | **City** | **Localities** | **28s** | CYTB | **LSM** | **PJH** | **UPCA** | **TRNA** | **CISP** |
| **650_Pcor** | *P. coreodes* | Brazil | Mato Grosso do Sul | Corumba | * | OM291436 | * | OM256875 | OM257026 | OM256947 | * |
| **652_Pcor** | *P. coreodes* | Brazil | Mato Grosso do Sul | Corumba | * | OM291437 | * | OM256876 | OM257027 | OM256948 | * |
| **654_Pcor** | *P. coreodes* | Brazil | Mato Grosso do Sul | Corumba | * | OM291438 | OM291394 | OM256877 | OM257028 | OM256949 | OM291494 |
| **655_Pcor** | *P. coreodes* | Brazil | Mato Grosso do Sul | Corumba | * | OM291439 | OM291395 | OM256878 | * | OM256950 | OM291495 |
| **658_Pcor_Bra_Mat_Cor** | *P. coreodes* | Brazil | Mato Grosso do Sul | Corumba | * | * | * | OM256879 | OM257029 | OM256951 | OM291496 |
| **660_Pcor** | *P. coreodes* | Brazil | Mato Grosso do Sul | Corumba | * | OM291440 | OM291396 | OM256880 | OM257030 | OM256952 | OM291497 |
| **662_Pcor** | *P. coreodes* | Brazil | Mato Grosso do Sul | Corumba | * | OM291441 | * | OM256881 | OM257031 | OM256953 | OM291498 |
| **663_Pcor** | *P. coreodes* | Brazil | Mato Grosso do Sul | Corumba | * | OM291442 | OM291397 | * | OM257032 | * | OM291499 |
| **664_Pcor** | *P. coreodes* | Brazil | Mato Grosso do Sul | Corumba | * | * | OM291398 | * | OM257033 | OM256954 | OM291500 |
| **665_Pcor_Bra_Mat_Cor** | *P. coreodes* | Brazil | Mato Grosso do Sul | Corumba | * | * | * | * | OM257034 | OM256955 | * |
| **666_Pcor_Bra_Mat_Cor** | *P. coreodes* | Brazil | Mato Grosso do Sul | Corumba | * | OM291443 | * | * | OM257035 | OM256956 | OM291501 |
| **667_Pcor_Bra_Mat_Cor** | *P. coreodes* | Brazil | Mato Grosso do Sul | Corumba | * | OM291444 | OM291399 | OM256882 | * | OM256957 | OM291502 |
| **669_Pcor_Bra_Mat_Cor** | *P. coreodes* | Brazil | Mato Grosso do Sul | Corumba | * | OM291445 | OM291400 | OM256883 | OM257036 | OM256958 | OM291503 |
| **670_Pcor_Bra_Mat_Cor** | *P. coreodes* | Brazil | Mato Grosso do Sul | Corumba | * | OM291446 | * | OM256884 | OM257037 | OM256959 | * |
| **671_Pcor_Bra_Mat_Cor** | *P. coreodes* | Brazil | Mato Grosso do Sul | Corumba | * | OM291447 | OM291401 | OM256885 | * | OM256960 | OM291504 |
| **672_Pcor** | *P. coreodes* | Brazil | Mato Grosso do Sul | Corumba | * | OM291448 | OM291402 | OM256886 | OM257038 | OM256961 | OM291505 |
| **679_Pcor** | *P. coreodes* | Brazil | Mato Grosso do Sul | Corumba | * | OM291449 | OM291403 | * | OM257039 | OM256962 | OM291506 |
| **680_Pcor_Bra_Mat_Cor** | *P. coreodes* | Brazil | Mato Grosso do Sul | Corumba | * | OM291450 | OM291404 | * | OM257040 | OM256963 | OM291507 |
| **681_Pcor** | *P. coreodes* | Brazil | Mato Grosso do Sul | Corumba | * | * | * | * | * | OM256964 | * |
| **682_Pcor_Bra_Mat_Cor** | *P. coreodes* | Brazil | Mato Grosso do Sul | Corumba | * | * | OM291405 | * | OM257041 | OM256965 | OM291508 |
| **683_Pcor_Bra_Mat_Cor** | *P. coreodes* | Brazil | Mato Grosso do Sul | Corumba | * | * | * | * | OM257042 | OM256966 | * |
| **688_Part_Ven_Ara_Mar** | *P. coreodes* | Brazil | Mato Grosso do Sul | Corumba | * | OM291451 | * | OM256887 | OM257043 | OM256967 | OM291509 |
| **689_Part_Ven_Ara_Mar** | *P. arthuri* | Venezuela | Aragua | Marbacella | OL584431 | * | * | OM256888 | * | OM256968 | OM291510 |
| **690_Part_Ven_Ara_Mar** | *P. arthuri* | Venezuela | Aragua | Marbacella | OL584432 | OM291452 | * | OM256889 | OM257044 | OM256969 | OM291511 |
| **691_Part_Ven_Ara_Mar** | *P. arthuri* | Venezuela | Aragua | Marbacella | OL584433 | OM291453 | * | OM256890 | OM257045 | OM256970 | OM291512 |
| **692_Part** | *P. arthuri* | Venezuela | Aragua | Marbacella | * | * | * | * | OM257046 | OM256971 | * |
| **693_Part_Ven_Ara_Mar** | *P. arthuri* | Venezuela | Aragua | Marbacella | OL584434 | * | * | OM256891 | OM257047 | OM256972 | OM291513 |
| **703_Pter** | *P. arthuri* | Venezuela | Aragua | Marbacella | * | * | OM291406 | OM256892 | OM257048 | OM256973 | OM291514 |
| **705_Part_Ven_Ara_Mar** | *P. arthuri* | Venezuela | Aragua | Marbacella | OL584435 | OM291454 | OM291407 | OM256893 | OM257049 | OM256974 | OM291515 |
| **706_Part_Ven_Ara_Mar** | *P. arthuri* | Venezuela | Aragua | Marbacella | OL584436 | OM291455 | * | * | * | OM256975 | OM291516 |
| **707_Part_Ven_Ara_Mar** | *P. arthuri* | Venezuela | Aragua | Marbacella | * | OM291456 | OM291408 | OM256894 | OM257050 | OM256976 | * |
| **709_Part_Ven_Ara_Mar** | *P. arthuri* | Venezuela | Aragua | Marbacella | * | * | OM291409 | OM256895 | OM257051 | OM256977 | OM291517 |
| **712_Part_Ven_Ara_Mar** | *P. arthuri* | Venezuela | Aragua | Marbacella | OL584437 | * | * | OM256896 | OM257052 | OM256978 | OM291518 |
| **727_Part** | *P. arthuri* | Colombia | Casanare | Paz de Ariporo | * | * | OM291410 | OM256897 | * | OM256979 | OM291519 |
| **728_Part** | *P. arthuri* | Colombia | Casanare | Paz de Ariporo | * | * | OM291411 | OM256898 | * | OM256980 | OM291520 |
| **729_Part_Col_Cas_Paz** | *P. arthuri* | Colombia | Casanare | Paz de Ariporo | OL584438 | * | * | OM256899 | * | OM256981 | * |
| **731_Part_Col_Cas_Tam** | *P. arthuri* | Colombia | Casanare | Tamara | OL584439 | * | * | OM256900 | * | OM256982 | OM291521 |
| **732_Part_Col_Cas_Tam** | *P. arthuri* | Colombia | Casanare | Tamara | OL584440 | * | OM291412 | OM256901 | * | OM256983 | OM291522 |
| **733_Part** | *P. arthuri* | Colombia | Casanare | Tamara | * | * | OM291413 | OM256902 | OM257053 | OM256984 | OM291523 |
| **737_Part_Col_Cas_Por** | *P. arthuri* | Colombia | Casanare | Pore | OL584441 | * | OM291414 | OM256903 | OM257054 | OM256985 | OM291524 |
| **738_Part_Col_Cas_Por** | *P. arthuri* | Colombia | Casanare | Pore | OL584442 | * | OM291415 | OM256904 | OM257055 | OM256986 | OM291525 |
| **742_Part_Col_Cas_Por** | *P. arthuri* | Colombia | Casanare | Pore | OL584443 | * | * | OM256905 | OM257056 | * | * |
| **759_Part_Col_Cas_Mon** | *P. arthuri* | Colombia | Casanare | Monterrey | OL584444 | * | OM291416 | OM256906 | OM257057 | OM256987 | OM291526 |
| **760_Part_Col_Cas_Mon** | *P. arthuri* | Colombia | Casanare | Monterrey | * | * | OM291417 | OM256907 | OM257058 | OM256988 | OM291527 |
| **761_Part** | *P. arthuri* | Colombia | Casanare | Monterrey | * | * | OM291418 | OM256908 | OM257059 | OM256989 | OM291528 |

| Table 24. GenBank accession numbers of seven loci analyzed in this study and samples origin included in this study | | | | | | | | | | | |
|---|---|---|---|---|---|---|---|---|---|---|---|
| **Sequence_ID** | ***Species*** | **Country** | **City** | **Localities** | **28s** | CYTB | **LSM** | **PJH** | **UPCA** | **TRNA** | **CISP** |
| **765_Part_Col_Ara_Ara** | *P. arthuri* | Colombia | Arauca | Arauca | OL584445 | * | OM291419 | OM256909 | OM257060 | OM256990 | OM291529 |
| **774_Rpro** | *R. prolixus* | Colombia | Arauca | Arauca | * | OM291457 | OM291420 | OM256910 | OM257061 | OM256991 | OM291530 |
| **775_Rpro** | *R. prolixus* | Colombia | Arauca | Arauca | * | OM291458 | OM291421 | OM256911 | OM257062 | OM256992 | OM291531 |

*These samples does not have sequences in this molecular marker
